# Triangulating Molecular Evidence to Prioritize Candidate Causal Genes at Established Atopic Dermatitis Loci

**DOI:** 10.1016/j.jid.2021.03.027

**Published:** 2021-11

**Authors:** Maria K. Sobczyk, Tom G. Richardson, Verena Zuber, Josine L. Min, Tom R. Gaunt, Lavinia Paternoster, eQTLGen Consortium, BIOS Consortium, GoDMC

**Affiliations:** 1 MRC Integrative Epidemiology Unit, Department of Population Health Sciences, Bristol Medical School, University of Bristol, Bristol, United Kingdom; 2 Department of Epidemiology and Biostatistics, School of Public Health, Imperial College London, London, United Kingdom; 3 MRC Biostatistics Unit, School of Clinical Medicine, University of Cambridge, Cambridge, United Kingdom

**Keywords:** AD, atopic dermatitis, bp, base pair, eQTL, expression quantitative trait locus, GTEx, Genotype-Tissue Expression, QTL, quantitative trait locus, STAT, signal transducer and activator of transcription, Th, T helper, TWAS, transcriptome-wide association study

## Abstract

GWASs for atopic dermatitis have identified 25 reproducible loci. We attempt to prioritize the candidate causal genes at these loci using extensive molecular resources compiled into a bioinformatics pipeline. We identified a list of 103 molecular resources for atopic dermatitis etiology, including expression, protein, and DNA methylation quantitative trait loci datasets in the skin or immune-relevant tissues, which were tested for overlap with GWAS signals. This was combined with functional annotation using regulatory variant prediction and features such as promoter‒enhancer interactions, expression studies, and variant fine mapping. For each gene at each locus, we condensed the evidence into a prioritization score. Across the investigated loci, we detected significant enrichment of genes with adaptive immune regulatory function and epidermal barrier formation among the top-prioritized genes. At eight loci, we were able to prioritize a single candidate gene (*IL6R*, *ADO*, *PRR5L*, *IL7R*, *ETS1*, *INPP5D*, *MDM1*, *TRAF3*). In addition, at 6 of the 25 loci, our analysis prioritizes less familiar candidates (*SLC22A5, IL2RA, MDM1, DEXI, ADO, STMN3*). Our analysis provides support for previously implicated genes at several atopic dermatitis GWAS loci as well as evidence for plausible additional candidates at others, which may represent potential targets for drug discovery.

## Introduction

Defined by inflamed dry, hyperplastic eczematous skin and pruritus, atopic dermatitis (AD) is among the world’s top 50 common diseases, with prevalence in 2010 estimated at close to 230 million cases and increasing (Hay et al., 2014). AD is highly heritable, with estimates of up to 75% in twin studies (Elmose and Thomsen, 2015). The largest and most recent GWAS of AD undertaken by the EAGLE (EArly Genetics and Lifecourse Epidemiology) consortium in 2015 identified 25 loci associated with AD in individuals of European descent (Paternoster et al., 2015). Majority of the disease-associated variants are located in noncoding regions, implying that they have a regulatory role rather than affecting protein function. Thus, integrating various biological data resources can provide complementary evidence about GWAS causal genes (Hormozdiari et al., 2018).

Since the publication of the AD EAGLE GWAS, there has been an explosion of new datasets from many cell types and new methods that offer an opportunity to refine the prioritization of genes at the GWAS loci. In this paper, we aim to comprehensively dissect AD GWAS loci by prioritizing candidate causal genes and illuminating biological mechanisms through which candidate genes can impact AD risk. We integrate several established fine-mapping and gene prioritization methods in a unique AD-focused gene prioritization pipeline to comprehensively evaluate the causal genetic evidence at each locus and utilize an exhaustive set of 103 molecular datasets in AD-relevant tissues to best support these methods. We explicitly model our assumptions about the importance of different types of evidence as well as the strength of the associations relating the features to genes and variants. In combining these methods, our pipeline generates a score for each gene used to assess the magnitude of evidence of each tested gene at a locus of being causal. Such a score can serve as a metric that allows rapid gene prioritization by molecular biologists and other interested parties, such as pharmaceutical companies.

## Results

### Identification of key tissues and cell types in AD GWAS loci

To determine which tissues and cell types should be part of the pipeline, we tested for enrichment of expression at our GWAS loci across a wide range of tissues and cell types (53 tissues from Genotype-Tissue Expression [GTEx], version 7, and 79,249,533 cell types from the Gene Atlas, Immunological Genomics, and FANTOM CAGE [Functional Annotation of the Mouse/Mammalian Genome Cap Analysis of Gene Expression]) and determined that all immune cell, skin (including fibroblast), spleen, and whole-blood datasets should be included (Supplementary Results). We reviewed the literature to identify 103 separate datasets from these tissue types with relevant data (Supplementary Figures S1 and S2).

### Prioritization of candidate genes

Gene prioritization scores ranged from 0 to 1,405 (SNP scores ranged from 0.5 to 968) (Dataset S1). For eight loci, the top-prioritized SNP was not the index SNP, and for 10 loci, the closest gene did not score best (Table 1). In detailing the results, we focus on genes ranked in the top 3 and SNPs ranked in the top 10 at each locus because this limit agrees with the sharp score decay observed in the scores (Supplementary Figures S3 and S4; Dataset S2).

**Table 1:** Genes Prioritized at Atopic Dermatitis GWAS Loci

Locus	GWAS Index Variant	Nearest Genes	Top-Ranked Gene	Second-Ranked Gene	Third-Ranked Gene
1q21.3 - a	rs61813875	*CRCT1*/*LCE3E*	*HRNR* (464, 28%)	*RPTN* (285, 17%)	*CRNN* (249, 15%)
1q21.3 - b	rs12730935	*IL-6R*	*IL-6R* (743, 62%)^1^	*UBE2Q1* (93, 8%)	*ADAR* (61, 5%)
2p13.3	rs112111458	*CD207*/*VAX2*	*CD207* (272, 45%)^1^	*CLEC4F* (62, 10%)	*VAX2* (56, 9%)
2q12.1	rs6419573/rs3917265^2^	*IL-18R1*/*IL-18RAP*	*IL-18R1* (1,384, 39%)^1^	*IL-18RAP* (1,341, 38%)^1^	*IL1RL1* (224, 6%)
2q37.1	rs1057258	*INPP5D*	*INPP5D* (296, 57%)^1^	*ATG16L1* (106, 20%)	*RN7SL32P* (29, 6%)
4q27	rs6827756/rs13152362^2^	*KIAA1109*	*KIAA1109* (220, 35%)^1^	*BBS12* (112, 18%)	*TRPC3* (100, 16%)
5p13.2	rs10214237	*IL-7R*/*CAPSL*	*IL-7R* (965, 65%)1	*SPEF2* (203, 14%)	*UGT3A2* (89, 6%)
5q31.1 - a	rs12188917	*TH2LCRR*	*SLC22A5* (461, 35%)	*IRF1* (303, 23%)	*RAD50* (122, 9%)
5q31.1 - b	rs4705962^2^	*KIF3A*	*KIF3A* (249, 23%)^1^	*SLC22A5* (247, 23%)	*PDLIM4* (142, 13%)
6p21.32	rs4713555	*STAT3*	*HLA-DRA* (1,405, 30%)	*HLA-DQB1* (689, 15%)	*HLA-DRB1* (566, 12%)^1^
6p21.33	rs41293864	*MICB*	*HSPA1B* (173, 15%)	*HCG27* (165, 14%)	*CSNK2B* (152, 13%)
8q21.13	rs6473227	*MIR5708*/*ZBTB10*	*ZBTB10* (192, 41%)^1^	*TPD52* (70, 15%)	*PAG1* (69, 15%)
10p15.1	rs6602364	*IL2RA*/*IL15RA*	*IL-2RA* (333, 45%)^1^	*RBM17* (111, 15%)	*PFKFB3* (51, 7%)
10q21.2	rs2944542	*ZNF365*	*ADO* (615, 61%)	*ZNF365* (101, 10%)^1^	*EGR2* (90, 9%)
11p13	rs2592555/rs12295535^2^	*PRR5L*	*PRR5L* (598, 79%)^1^	*TRAF6* (65, 9%)	*COMMD9* (34, 5%)
11q13.1	rs10791824	*OVOL1*	*CTSW* (336, 23%)	*OVOL1* (236, 16%)^1^	*EFEMP2* (168, 11%)
11q13.5	rs2212434	*C11orf30*/*LRRC32*	*LRRC32* (545, 43%)^1^	*EMSY* (521, 41%)^1^	*THAP12* (47, 4%)
11q24.3	rs7127307	–/*ETS1*	*ETS1* (298, 75%)^1^	*FLII* (35, 9%)	*APLP2* (18, 5%)
12q15	rs2227483	*IL22*	*MDM1* (728, 70%)	*IL-22* (99, 10%)^1^	*IFNG* (57, 5%)
14q13.2	rs2038255	*PPP2R3C*	*PPP2R3C* (996, 31%)^1^	*KIAA0391* (814, 25%)	*SRP54* (433, 13%)
14q32.32	rs7146581	*TRAF3*	*TRAF3* (848, 55%)^1^	*AMN* (281, 18%)	*CDC42BPB* (186, 12%)
16p13.13	rs2041733	*CLEC16A*	*DEXI* (376, 34%)	*CLEC16A* (364, 33%)^1^	*RMI2* (108, 10%)
17q21.2	rs12951971	*STAT3*	*DHX58* (254, 32%)	*STAT3* (101, 13%)^1^	*RAB5C* (100, 13%)
17q25.3	rs11657987	*PGS1*	*PGS1* (205, 46%)^1^	*DNAH17* (73, 16%)	*SOCS3* (52, 12%)
19p13.2	rs2918307	*ADAMTS10*/*ACTL9*	*ACTL9* (115, 41%)^1^	*ADAMTS10* (57, 20%)^1^	*MAP2K7* (34, 12%)
20q13.33	rs4809219	*RTEL1*/*TNFRSF6B*	*STMN3* (608, 27%)	*LIME1* (473, 21%)	*ARFRP1* (257, 12%)

Abbreviation: STAT, signal transducer and activator of transcription.

The two values given in parentheses in the top three ranked gene columns correspond to the gene prioritization score and the percentage of the total score for locus top 10 genes.

1 The closest genes to the index variant (in either direction).

2 Index SNP for secondary signal, where the pipeline did not give different gene prioritizations for the two signals; these are presented on one row.

**Figure 1:**
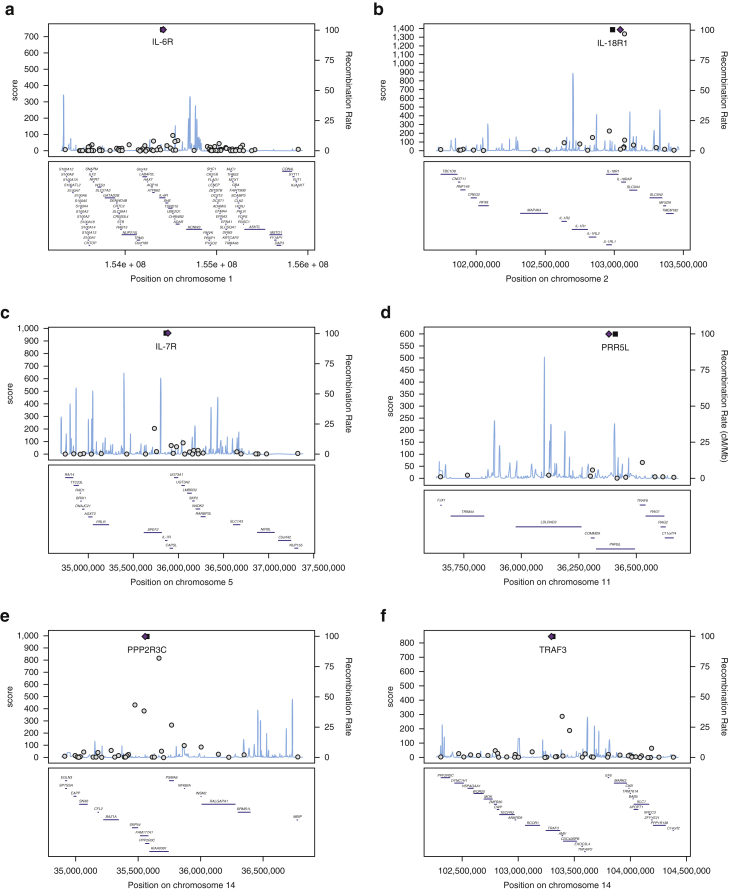
**Gene scores within the 3 Mbp interval of lead SNP in the six highest-scoring loci.** Top-prioritized gene marked with a black square and lead SNP marked with a purple diamond. (**a**) locus 1q21.3 – b; (**b**) locus 2q12.1; (**c**) locus 5p13.2; (**d**) locus 11p13; (**e**) locus 14q13.2; (**f**) locus 14q32.32. cM/Mb, centimorgan/mega base; Mbp, mega base pair.

Excluding the complex major histocompatibility complex locus, the highest gene scores were seen for genes at five loci: *IL18R1* (score = 1,384) and *IL18RAP* (score = 1,341) at 2q12.1 locus, *PPP2R3C* (score = 996) at 14q13.2 locus, *IL7R* (score = 965) at 5p13.2 locus, *TRAF3* (score = 848) at 14q32.32 locus, and *IL6R* (score = 743) at 1q21.3 locus (Table 1 and Figure 1; Dataset S3 for all loci). Assuming that the true model is one of a single causal gene at each locus, prioritization can also be evaluated by comparing the score of the top-prioritized gene at a locus with all other genes at that locus. Eight loci (1q21.3-*IL6R*, 10q21.2-*ADO*, 11p13-*PRR5L*, 5p13.2-*IL7R*, 11q24.3-*ETS1*, 2q37.1-*INPP5D*, 12q15-*MDM1*, 14q32.32-*TRAF3*) (Table 1) have a single stand-out candidate causal gene, with the top gene contributing >50% of the total score of the top 10 ranked genes. The top candidate by that metric is *PRR5L* (79% of top 10 genes at 11p13 locus), with a score of 598 compared with a score of 65 for the second-ranked gene at this locus. Most top-prioritized genes by the total score are also prioritized by this metric. Two further loci show good evidence (>75% cumulative score) shared across two candidate genes (*IL18R1* and *IL18RAP* at 2q12.1 and *EMSY* and *LRRC32* at 11q13.5, which share 77% and 84% of the cumulative score, respectively). At 2q12.1 (where *IL18R1* and *IL18RAP* reside), there is evidence for two independent genetic signals, and these may affect each of the prioritized genes.

For five loci, the pipeline prioritizes the genes in the top position (and with a score >300) that were not considered in the original GWAS annotation (Paternoster et al., 2015): *MDM1* at 12q15 (score = 728), *ADO* at 10q21.2 (score = 615), *STMN3* at 20q13.33 (score = 608), *SLC22A5* at 5q31.1 (score = 461), and *DEXI* at 16p13.13 (score = 376). Some in this list (such as *SLC22A5*) represent promising candidates.

**Figure 2:**
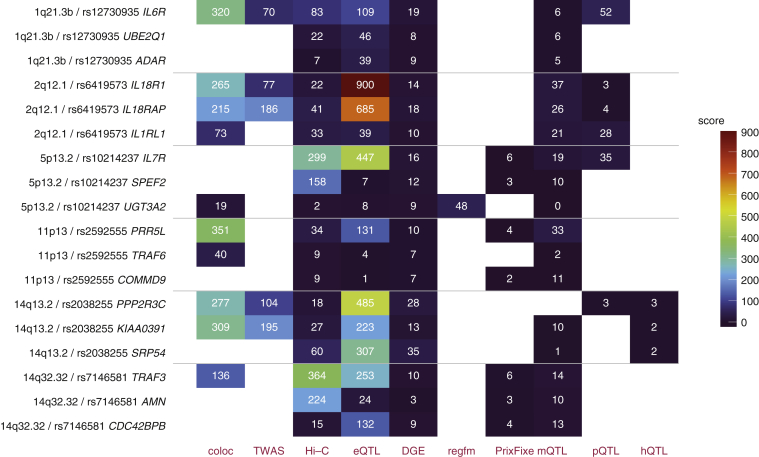
**Scores by type of evidence for the top three ranked genes in the six highest-scoring loci.** Scores for the top three ranked genes at each locus are shown, partitioned by the category of evidence—including in this figure the top 10 categories contributing the highest proportion of total score at the top 10 ranked genes for all the loci. The order of loci corresponds to the order in Table 1. DGE, differential gene expression; eQTL, expression quantitative trait loci; hQTL, histone quantitative trait loci; mQTL, DNA methylation quantitative trait locus; pQTL, protein quantitative trait loci; TWAS, transcriptome-wide association study.

For each locus as well as evaluating the overall prioritization scores of each gene, we present a summary figure that shows how different evidence sources have contributed to the overall score (Supplementary Figure S5); the loci with the most compelling evidence are displayed in Figure 2. In addition, the individual results from each source are also available for deeper evaluation (Dataset S4). A full discussion of each locus in Table 1 integrating evidence from the pipeline with knowledge from literature is available in Supplementary Results.

### Validation of gene prioritization

**Figure 3:**
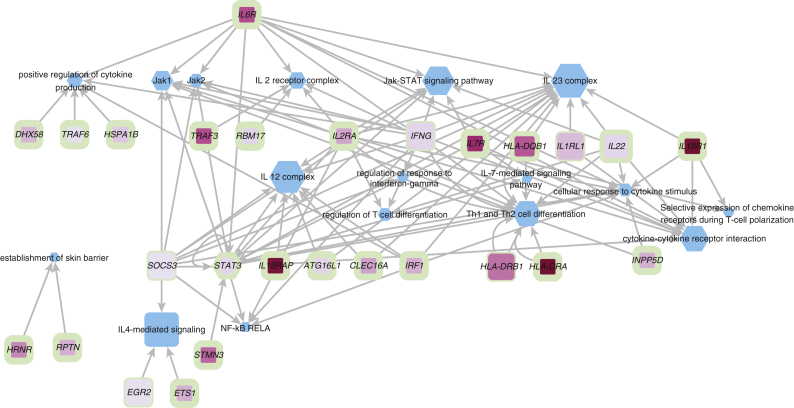
**Network visualization of the functional terms enriched among locus top three prioritized genes.** The ontology categories are depicted as blue hexagons, with their size linearly proportional to ‒log_10_ of adjusted enrichment *P*-value. AD genes are depicted as pink rectangles, with the intensity of the color fill proportional to the gene score and the thickness of the green border marking the gene rank at the locus, with rank 1 the thickest. AD, atopic dermatitis; STAT, signal transducer and activator of transcription; Th, T helper.

In the absence of gold-standard true positive genes with which we could compare our prioritization of candidate genes at GWAS loci, we evaluated our results in two indirect ways. First, we tested whether our top three prioritized genes across all loci are enriched in any gene sets using enrichr (Kuleshov et al., 2016) and compared those with the categories enriched among previously implicated AD genes (Supplementary Table S1). We found that both lists are significantly enriched for immune system‒related genes (Figure 3) but often with stronger evidence in our prioritized gene sets. In particular, cytokine categories were over-represented, for example, Gene Ontology cytokine‒mediated signaling pathway (adjusted *P*-value for our prioritized genes = 1 × 10^−9^ vs. 0.004 for other previously implicated AD genes). The genes in the cytokine pathways identified by the pipeline include *IL6R*, *IL22, INPP5D, IL2RA*, *IFNG*, *IL18R1*, *IL18RAP*, *IL1RL1,* and *IL7R*. Signaling involving the regulation of response to IFN-γ (Gene Ontology, *P* = 0.039 vs. 0.043), Jak1/ Jak2/signal transducer and activator of transcription (STAT) 3‒interacting genes, and Jak‒STAT signaling pathway in general (Kyoto Encyclopedia of Genes and Genomes, *P* = 4 × 10^−5^ vs. 2 × 10^−4^), also overlapped between the two gene sets, as did terms relating to T-cell differentiation. We did not find enrichment of genes in any specific type of immunity, including in all of T helper (Th)1, Th2, Th17, and Th22 represented and previously shown to play a role in certain subsets of patients with AD, despite the overall particular importance of Th2 and Th22 (Esaki et al., 2016; Leung and Guttman-Yassky, 2014; Suárez-Fariñas et al., 2013). Genes concerned with the establishment of the skin barrier were marginally enriched for in the pipeline (owing to the prioritization of cornified envelope genes *HRNR* and *RPTN*) but less than the previously reported AD genes (Gene Ontology, *P* = 0.045 vs. 8 × 10^−8^) (Supplementary Table S2).

The second way we validated our results was to test whether our candidates interacted with each other and with the genes with established roles in AD pathogenesis using STRING (Search Tool for the Retrieval of Interacting Genes/Proteins) (Szklarczyk et al., 2019) to visualize the highest-confidence interactions. The analysis revealed an extensive network that included 25 prioritized genes, centered on key immune regulators (Supplementary Results and Supplementary Figure S6).

## Discussion

Previous annotations of AD GWAS loci have been limited in their ability to identify likely causal genes (Paternoster et al., 2015). In this paper, we provide a thorough investigation of the 25 European AD loci by integrating all relevant available data that can be used to provide evidence for hypothesizing causal genes and combine these data in such a way as to produce a ranking for every gene at each locus.

Because there are a vast number of methods that can be employed to attempt to establish the causal genes for GWAS signals, we integrate several of these, which represent the most useful and robust approaches that span experimentally generated functional annotations, predictions for regulatory impact generated by machine learning models, as well as linking back to AD physiology through the evaluation of differential gene expression and DNA methylation studies and proteome comparisons involving patients with eczema.

We employed the most robust methods where possible; for example, statistical methods (coloc and transcriptome-wide association study [TWAS]) were used to formally compare the association patterns in quantitative trait loci (QTL) studies with those in GWAS when full summary statistics were available because ∼50% of common variants are associated with one expression QTL (eQTL) or more across 53 tissues in GTEx (Liu et al., 2019), so simple lookups for variant overlap alone will result in many false positives. Where full summary statistics were not available, we still included such lookups but gave such evidence much lower weight in the overall score (weight adjustment of 2 compared with that of 20 for colocalization).

For 10 loci, the top-ranked gene is not the gene closest to the index GWAS SNP. Eight loci have a single stand-out candidate causal gene (score >50% of the top 10 gene cumulative scores), and seven genes score particularly high (>700) and/or have a particular stand-out score (>75%). Although our analysis strengthens the evidence for existing candidate causal genes at these loci in many cases, at six loci, our score ranks alternative candidates as the most likely causal gene.

One of these six loci can be considered an interesting validation of our approach. *IL15RA* was previously considered the most plausible candidate gene at the 10p15.1 locus owing to the limited eQTL evidence that was available at the time. Our approach however prioritized *IL2RA* over *IL15RA*. Since the publication of the GWAS in 2015, this locus has been followed up with CRISPR experiments, which reported that the T-allele at rs61839660 downregulates *IL2RA* expression (Simeonov et al., 2017), suggesting that our prioritization at this locus is correct.

At another locus—11q13.5—experimental evidence has emerged, supporting the candidate role of the top two prioritized genes—*LRRC32* (encoding the GARP receptor) and *EMSY*. Rare missense mutations found in *LRRC32* in patients with eczema decrease GARP expression on the activated T-regulatory cell surface and reduce the conversion of naive T cells into T-regulatory cells (Manz et al., 2016). In contrast, *EMSY* has been characterized as a potent regulator of skin barrier formation (Elias et al., 2019). Another top-prioritized gene with recent evidence for a role in skin barrier formation is *KIF3A* (locus 5q31.1b) (Stevens et al., 2020); further details are provided in Supplementary Results.

Other validations of our approach are provided by tests of enrichment of ontology terms and evidence of protein‒protein interactions among the top-ranked genes across all loci. Enrichment was found for ontology terms associated with skin barrier integrity, Th cell polarization, cytokine signaling, and Jak‒STAT signaling. The importance of Jak‒STAT signaling has recently been highlighted by its enrichment among genes prioritized for inflammatory skin diseases (including AD) with Hi-chromatin immunoprecipitation‒derived T-cell enhancer connectome (Jeng et al., 2019) and over-representation of rare coding variants in Jak1 and/or Jak2 in a new AD study (Mucha et al., 2020). In investigating protein‒protein interactions with the STRING database among our prioritized candidate genes and other established candidates, interactions between genes with immune regulatory (but not with skin barrier) functions were found among the established AD players: *TSLP* and its receptor, *TLR2*, *STAT6*, *IL4,* and *IFNGR*. STRING data are not entirely comprehensive and omits other functional relationships between prioritized genes, described in Supplementary Results.

In general, the results of our GWAS prioritization analysis remind us that interpretation of a GWAS locus is complicated owing to varying regulation between cell types and widespread coregulation that makes identification of the true causal gene difficult. Indeed, recent GWAS research reveals that on top of each locus being able to contain multiple signals (Mahajan et al., 2018), each signal can influence multiple coregulated genes (Cannon and Mohlke, 2018). Associations with molecular phenotypes follow the same pattern, with at least 9% of human eQTLs quantified to contain secondary signals (Wood et al., 2011) and multiple genes implicated for 50% of human eQTLs (Gamazon et al., 2018). According to the multiple enhancer variant hypothesis, several variants in linkage disequilibrium can influence multiple enhancers and cooperatively affect the expression of target gene(s). Corradin et al. (2014) provide evidence for it in six autoimmune diseases, including rheumatoid arthritis, Crohn's disease, and systemic lupus erythematosus. Therefore, it is not surprising that many of our loci showed multiple colocalizations for different genes and tissues, especially in gene-dense regions, with the caveat that not all may be causal. A recent analysis of the TWAS colocalization method claims that around 75% of hits will be noncausal in the instance of correlated gene expression at the locus (Wainberg et al., 2019), and we hypothesize that that may be the case at loci 11q13.1, 14q13.2, and 20q13.33, where the expression of as many as 4‒6 genes colocalizes with AD GWAS signal in the TWAS results. Still, owing to a distinct possibility of detection of multiple target genes and variants at a locus, we do not focus only on the top-rated hits in our gene and variant ranking. AD GWAS loci that we believe should be further experimentally investigated in that regard include 2q12.1 (*IL18R1*, *IL18RAP*, *IL1R1*), 5q31.1 (*KIF3A*, *PDLIM4*, *SLC22A4*, *IRF1*), and 20q13.33 (*STMN3*, *LIME1*, *ARFRP1*), especially the first two because they contain at least two independent signals in the GWAS analysis.

Most of the genes with eQTL colocalization across tissues exhibit the same direction of effect, for example, *PRR5L* (at 11p13), where the protective allele is associated with increased expression in the skin, whole-blood, and immune cell subsets. However, at three loci (2q12.1, 14q13.2, and 20q13.33), there may be tissue-dependent effects on expression, with opposite directions of effect on *STMN3, LIME1, APFRP1, IL18RAP,* and *PP2R3C*. This indicates that causal variants potentially reside in tissue type‒specific regulatory regions and that the context-dependent effect of these genes could impact AD phenotype.

Although focused on the integration of AD-relevant resources in this use case, our pipeline for follow-up of GWAS signals can be adapted for other diseases or traits after the identification of the most relevant molecular datasets. The best evidence would come from consistent and clear prioritization of a single gene from multiple sources (e.g., variants of interest at a locus showing physical interaction with enhancers and promoters of the same genes implicated by eQTL and protein QTL data and validation of such genes in differential expression analyses, all in consistent cell and/or tissue types). However, for several reasons, this situation is uncommon. Available datasets include evidence from limited tissues and cell states, reflecting transcriptional dynamics, which are often transient, and low base pair (bp) resolution offered by high-throughput Hi-C, which results in large, nonspecific overlap regions (Mora et al., 2016). Ideally, data on specific blood and skin cell types would be available rather than those on bulk tissue, which will average out any cell-specific signals (Cano-Gamez and Trynka, 2020). Furthermore, available sources do not cover the full spectrum of variants or genes and/or proteins, and so the absence of evidence cannot be equated to evidence of absence. Predictions will improve as evidence from across more tissue types, especially at a single-cell resolution, become available. Such rich datasets are already being generated for related disorders, such as asthma (Vieira Braga et al., 2019), considering trans- and isoform-level mechanisms of action and explicitly modeling network connectivity through protein‒protein interactions and coexpression. It is also important to note that all the methods described in the pipeline are purely correlational and so will require experimental manipulation for establishing causality of target genes through, for example, CRISPR screening.

Our gene prioritization score method assigns weight to different evidence sources, effectively upweighting evidence with expected lower false discovery rate (such as TWAS and coloc), which are also rarer, and downweighting weaker evidence such as single eQTL lookups, which have been shown to often be purely coincidental and are numerous, and so could easily overwhelm the overall score. There is currently no consensus on the best way to quantitatively integrate such evidence. Previous efforts for single-trait GWAS annotation have taken other approaches: assigning equal weights (Schlosser et al., 2020), which has obvious downsides, or attempting automatic weight assignment (Schwartzentruber et al., 2021), which is essentially optimized for closest genes. A promising approach uses gold-standard gene assignments at select GWAS loci for training (Ghoussaini et al., 2021); however, this type of method requires a number of GWAS as input, with evidence sources limited to those relevant to all traits and selection bias inherent to the choice of gold-standard genes used for training. It is of note that many different approaches all upweight the colocalization evidence, in agreement with our pipeline. Although there is some arbitrariness in our weighting assumptions, we believe our score calculation procedure has clear assumptions and justifiably balances some of the tradeoffs.

Although there are limitations in our approach, as outlined in earlier sections, we find it useful as an approach to easily flag the genes where we find most evidence, which can then be carefully evaluated and potentially characterized as future drug targets. Loci where we are more confident in prioritization of single genes especially lend themselves to direct experimental investigation, such as *TRAF3* at the 14q32.32 locus and *PRR5L* at the 11p13 locus. In addition, investigating the loci with clear candidate genes and association with multiple inflammatory diseases showing a consistent direction of effects, such as 11p13 (*PRR5L*—multiple sclerosis, asthma), 11q24 (*ETS1*—psoriasis, celiac disease), and 16p13.13 (*DEXI* and *CLEC16A—*type-I diabetes, multiple sclerosis, alopecia areata, systemic lupus erythematosus, asthma), may reveal promising targets with potential drug repurposing future. Others with opposing direction of effect may reveal the potential adverse side effects for consideration in therapeutic development (e.g., with anti–IL-6 biologics for rheumatoid arthritis).

## Materials and Methods

The materials and methods discussed in this section are an abridged version. For additional technical details, see Supplementary Materials and Methods.

### Source GWAS

We investigate 25 loci, which either show a genome-wide significance and are for novel loci replicated in independent European ancestry sample (21 loci) or are significant loci prioritized by the gene set enrichment analysis presented in the original paper (Paternoster et al., 2015).

### Bayesian fine mapping

To identify the likely causal genetic variants in the regions harboring AD GWAS signals, we used three different Bayesian fine-mapping methods: Finemap (Benner et al., 2016), fastPaintor (Kichaev et al., 2017), and JAM (Newcombe et al., 2016). Each method relies on different previous assumptions and model formulation leading to divergent results (Cannon et al., 2017). The aim of our fine mapping was not necessarily to identify the causal variants per se but to prioritize SNPs, which in turn provide evidence for what genes in the region are likely to be causal (further details are provided in Supplementary Materials and Methods).

### Variant filtering

In subsequent gene analyses (described later), we limited ourselves to the SNPs within the region in significant linkage disequilibrium with the index SNP in 1000 Genomes European population, which is referred to as the GWAS locus interval in the remaining part of this paper. The region in each case was defined by the positions of the furthest-away 5′ and 3′ SNPs with r^2^ ≥ 0.2 relative to those of the index SNP (limited to a maximum of 500 kilobases in either direction). All the SNPs within that boundary were then considered (further details are provided in Supplementary Materials and Methods).

### Identification of key tissues and cell types

To focus on the key tissues and/or cell types associated with eczema variants, first, we used gene set enrichment in SNPSea (Slowikowski et al., 2014) with the supplied gene expression datasets: Gene Atlas Affymetrix expression in 79 human tissues (Su et al., 2004), Immunological Genome Project (Heng et al., 2008) Affymetrix expression in 249 murine blood cell types, and FANTOM CAGE (Kawaji et al., 2014) in 533 human cell types.

Second, we used MAGMA (de Leeuw et al., 2015) gene enrichment analysis on GTEx 7.0 (GTEx Consortium et al., 2017) data as carried out by FUMA (Watanabe et al., 2017) (further details are provided in Supplementary Materials and Methods).

### eQTL identification

We used genotype array data and RPKM (reads per kilobase of transcripts per million mapped reads), normalized expression in lymphoblastoid cell line, and skin tissue from the TwinsUK cohort (Buil et al., 2015). *cis*-eQTLs 1.5 mega bp upstream and downstream of transcriptional start site were identified using linear mixed model implemented in GEMMA (Genome-wide Efficient Mixed Model Association) (Zhou and Stephens, 2012). eQTL associations were identified using the Wald test.

In the analysis involving the CEDAR (Center for Diet and Activity Research) cohort (Momozawa et al., 2018), we used the publicly available data: imputed genotypes and normalized gene expression values from blood and intestinal cell types (CD4^+^ T lymphocytes, CD8^+^ T lymphocytes, CD19^+^ B lymphocytes, CD14^+^ monocytes, CD15^+^ granulocytes, platelets, ileum, colon, rectum). We used GEMMA's linear mixed model and Wald test to reidentify *cis*-eQTLs within 1.5 mega bp upstream and downstream of transcriptional start site (further details are provided in Supplementary Materials and Methods).

### Colocalization with coloc and TWAS

We obtained full summary statistic results for *cis*-eQTLs detected in whole blood in the eQTLGen dataset (Võsa et al., 2018^1^) (accessed on 8 August 2018); eQTLs from GTEx, version 7, dataset identified in the following tissues: whole blood, spleen, sun-exposed and -unexposed skin, transformed fibroblasts, and Epstein‒Barr virus‒transformed lymphocytes; eQTLs published from the study investigating monocyte response to microbe-associated molecular patterns (Kim-Hellmuth et al., 2017); eQTLs in the monocytes, neutrophils, and CD4^+^ T cells from the BLUEPRINT project (Chen et al., 2016); and protein QTLs from whole blood in the Sun et al. (2018) dataset as well as TwinsUK and The Center for Diet and Activity Research eQTLs identified earlier (Dataset S5). Subsequently, the colocalization signal between betas from GWAS and eQTLs and/or protein QTLs for genes within 1.5 mega bp upstream and downstream of index SNP was evaluated with the coloc (Giambartolomei et al., 2014) R package. In coloc analysis, we considered the loci with a posterior probability of hypothesis 4 (H4) > 0.5 as informative enough to be included (Supplementary Table S3), as done previously (Kim-Hellmuth et al., 2020); with H4 stating the hypothesis of both traits being associated and sharing a single causal variant.

We also carried out a TWAS (Gusev et al., 2016) analysis, where reference datasets with gene expression and genotype data (GTEx, version7.0; CEDAR; and TwinsUK) were used to predict the gene expression in our target GWAS. The analysis pipeline for the Summary-based Mendelian Randomization analysis has been described previously (Richardson et al., 2020) (further details are provided in Supplementary Materials and Methods).

### Complementary gene prioritization methods

To further prioritize the GWAS gene targets, we used two gene prioritization methods: regfm (Shooshtari et al., 2017) and PrixFixe (Taşan et al., 2015). PrixFixe strategy relies on the prioritization of groups of candidate genes from multiple GWAS loci on the basis of cofunction networks. Regfm’s workflow involves the intersection of fine-mapped credible interval SNPs with consensus DNase 1 hypersensitive sites and genes whose expression they control predicted on the basis of ROADMAP (Roadmap Epigenomics Consortium et al., 2015) chromatin accessibility and gene expression data to prioritize target genes.

### Variant functional prediction

KGGSeq (Li et al., 2016) was used to measure noncoding variant regulatory potential and coding variant deleteriousness using functional scores derived by combining the scores from seven algorithms. fathmm-XF (Rogers et al., 2018), GWAS4D (Huang et al., 2018), and fitCons (Gulko et al., 2015) were also used independently. Overlap with chromatin immunoprecipitation sequencing‒defined binding sites of transcriptional regulators was cross referenced in the ReMap2018 database (Chèneby et al., 2018). Splicing regulatory potential of variants was evaluated with SPIDEX (Xiong et al., 2015).

We also looked at variant overlap within different regulatory regions: insulator (Wang et al., 2015), promoter‒enhancer interactions (nine studies), regulatory noncoding RNAs (five studies), topologically associating domains (six studies), and CTCF binding sites (Ziebarth et al., 2013) using giggle (Layer et al., 2018) search engine (further details are provided in Supplementary Materials and Methods).

### Independent lookups

We have also performed gene and variant lookups among published significant results (see Dataset S5 for references) from 29 eQTL studies, three methylation quantitative trait locus (including GoMDC [Genetics of DNA Methylation Consortium] results [Min et al. 2020^2^]), two protein QTL studies, two histone QTL studies, and a chromatin accessibility QTL study where full GWAS results were not available as well as differential expression (five studies), DNA methylation (two studies), and two proteome comparisons in the skin between patients with AD and that in healthy controls. We also interrogated the GWAS catalog (MacArthur et al., 2016) (accessed on 11 January 11 2019) for any variants that have been identified as genome-wide significant in previous GWASs on related inflammatory conditions (further details are provided in Supplementary Materials and Methods).

### Generation of candidate gene and SNP rankings

The results of analyses and lookups listed earlier were then integrated to provide two rankings of (i) all the SNPs within each GWAS locus interval and (ii) all the genes within a 3-mega-bp window centered around index SNP. This was achieved by assigning a score to each piece of evidence and summing across these sources to generate a causal prioritization score for every SNP and every gene tested. These scores represent the strength of evidence for a causal role of the SNP or gene in AD. The detailed method of calculation of basic score per gene or variant in a given experiment and/or analysis is presented in Supplementary Materials and Methods and is visualized in Supplementary Figure S1. Briefly, each source of evidence was assigned a weight on the basis of subjective strength of evidence: highest (20) for results from statistical tests using a full set of summary statistics, such as molecular QTL colocalization methods; lowest (1) for prediction results from machine learning models, such as variant functional prediction software; and intermediate (2) for positional overlap with significant experimental results, such as identified promoter‒enhancer loops. In calculating the final score, we also considered the magnitude of the result significance or effect, the specificity (overall number of SNPs and/or genes significant in a given experiment), and the independence of the evidence (the number of experiments conducted in the same study, such as measuring both expression and DNA methylation levels). The final score was adjusted by the heterogeneity of the evidence (i.e., genes or variants consistently supported by a range of evidence sources—alternative functional assays and statistical methods—were upweighted in proportion to the square root of the mean number of unique study types and unique study identifications) as well as by the absolute number of studies providing supportive evidence.

### Data availability statement

All the code written to carry out the analysis is archived under https://doi.org/10.5281/zenodo.3775865. Datasets related to this article can be found in the following Figshare repositories:

1. Dataset S1. Full gene and variant loci rankings, which can be found in https://doi.org/10.6084/m9.figshare.12130824 for the genes and in https://doi.org/10.6084/m9.figshare.12130857 for the variants;
2. Dataset S2. Data evidence for the top 10 ranked gene and variants at each locus, which can be found in https://doi.org/10.6084/m9.figshare.12130863 for the genes and in https://doi.org/10.6084/m9.figshare.12130878 for the variants;
3. Dataset S3. Locus Zoom‒style gene and variant score plots for each locus, which can be can found in https://doi.org/10.6084/m9.figshare.12221006 for the genes and in https://doi.org/10.6084/m9.figshare.12221033 for the variants;
4. Dataset S4. The final evidence dataset used in ranking the genes and the variants, which can be found in https://doi.org/10.6084/m9.figshare.12130701; and
5. Dataset S5. Reference table detailing the individual datasets used, which can be found in https://doi.org/10.6084/m9.figshare.12222731.

## ORCIDs

Maria K. Sobczyk: http://orcid.org/0000-0003-0000-4100

Tom G. Richardson: http://orcid.org/0000-0002-7918-2040

Verena Zuber: http://orcid.org/0000-0001-9827-1877

Josine L. Min: http://orcid.org/0000-0003-4456-9824

Tom R. Gaunt: http://orcid.org/0000-0003-0924-3247

Lavinia Paternoster: http://orcid.org/0000-0003-2514-0889

## Conflict of Interest

TRG receives funding from GlaxoSmithKline and Biogen for unrelated research. The remaining authors state no conflict of interest.

## Footnotes

1 Võsa U, Claringbould A, Westra H, Bonder MJ, Zeng B, Kirsten H, et al. Unraveling the polygenic architecture of complex traits using blood eQTL meta- analysis. bioRxiv 2018.

2 Min JL, Hemani G, Hannon E, Dekkers KF, Castillo-Fernandez J, Luijk R, et al. Genomic and phenomic insights from an atlas of genetic effects on DNA methylation. medRxiv 2020.
