# Supplementary material for: Triangulating Molecular Evidence to Prioritize Candidate Causal Genes at Established Atopic Dermatitis Loci
Source: J Invest Dermatol. 2021 Nov;141(11):2620–9. doi: 10.1016/j.jid.2021.03.027 (PMC8592116; doi:10.1016/j.jid.2021.03.027)
Supplement: Supplementary Data Supplementary Materials [file mmc1.pdf]

SUPPLEMENTARY RESULTS

Identification of key tissues and cell types in AD GWAS loci

Skin, as the primary affected organ, and blood cell types, given the established immune component, are of relevance for AD (Boguniewicz and Leung 2011). We also identified additional tissues by using gene-set enrichment on gene expression datasets across available tissue/cell types to identify any tissue/cell-specificity enriched in genes linked to our GWAS loci.

MAGMA gene-property analysis on 53 tissue types from GTEx ver. 7 identified significant enrichment (at $p < 0.001$) for genes with tissue-specific expression in EBV-transformed lymphocytes, whole blood, spleen, sun-exposed and non-exposed skin at our GWAS loci. Despite not reaching statistical significance, we also included transformed fibroblasts ($p = 0.1$), due to the role of dermal fibroblasts in skin maintenance and repair (Driskell et al. 2013).

SNPSea enrichment analysis in the Gene Atlas dataset prioritized (at permuted $p < 0.05$,) whole blood and blood cell types: CD4+ T cells, CD14+ monocytes, CD8+ T cells, and dendritic cells. In addition, Immunological Genomics prioritized Natural Killer T cells, while FANTOM CAGE dataset prioritized stimulated monocytes, basophils, Mast cells, eosinophils, neutrophils, Langerhans cells, CD34+ progenitor cells, hair follicle outer root sheath cells as well as spleen. For that reason, we included datasets involving all possible types of immune cell datasets in our analysis.

In order to independently establish if restriction of the tissues to those known to be mechanistically linked to eczema and enriched in our GWAS signal in MAGMA and SNPSea analysis was likely to have influenced the final gene ranking, we compared our results to those run on the full set of 53 GTEx tissues, Blueprint and eQTLGen datasets using Summary-based Mendelian Randomization-based colocalization method, SMR. Twelve out of 20 (Table S4) genes showing strong evidence ($p < 6.5x10-6$) for colocalization using SMR were among the top 3 hits for a given locus from our analysis. Of the remaining 8, 3 were prioritized by SMR in tissues absent in the pipeline: the established eczema filaggrin gene in aorta artery (ranked 6 at 1q21.3 - a locus), its antisense transcript *FLG-AS1* in esophagus muscularis (ranked 21 at the same locus), and *RTEL1* helicase in tibial and aorta artery (ranked 15 at the 20q13.33 locus). However, further investigation of these eQTL signals suggest some may not be biologically meaningful due to very low expression levels of these genes in the respective tissues (*FLG* TPM 0.59 in aorta artery and *FLG-AS1* TPM 0.62 in esophagus muscularis in GTEx) Therefore, restriction to six GTEx tissues is unlikely to have had significant impact on the final gene ranking.

Prioritization summary for AD loci

Locus 1q21.3

At least two signals are known to be present in this epidermal differentiation complex region, rich in ~45 genes with roles in late cornified envelope (LCE) formation (Hoffjan and Stemmler 2007). One signal, represented by GWAS index SNP rs61812715 (or rs12144049 in Schaarschmidt et al. 2015), likely represents the known filaggrin (*FLG*) mutations, as the signal disappears after conditioning on the four most common substitutions (Paternoster et al. 2015). We hereby refer to this signal as locus 1q21.3 - a. The second independent signal is represented by rs12730935 (Paternoster et al. 2015). We refer to this signal as locus 1q21.3 – b.

Locus 1q21.3 - a

We considered 151 genes within 3 Mbp of index SNP rs61812715 in 1q21.3 - a to be potential candidate genes (Dataset S1). Whilst the causal gene at this locus has been shown to be *FLG* (Paternoster et al. 2015), our analysis was fairly inconclusive (with several genes with similar moderate scores) and the results gave three alternative filaggrin-like genes as the top prioritized loci. Top was hornerin (*HRNR*, score 464), which accounted for 28% of the cumulative score of the top 10 genes (Table 1), followed by repetin (*RPTN*, score=285) and cornulin (*CRNN*, score=249), which respectively account for 17% and 15% of the top 10 score. *FLG* was ranked 6 (score=108). Our gene colocalization analysis (Table S3) was not conclusive. It showed some evidence for *FLG* (AD effect signal colocalized with reduced expression in skin, TWAS $p = 1x10^{-11}$), but also for other genes, *CRNN*, *FLG-AS1*, *IVL*, *LCE1E*, *SPRR1B* and *SPRR2D*. The strongest evidence being for CRNN - a filaggrin-like protein (effect signal colocalized with increased expression, TWAS $p = 1x10^{-20}$). Our inconclusive findings in this region are likely due to the complex and highly duplicate nature of the differentiation complex, with many homologous genes, probably under coordinated regulation. Although conditional analysis in the EAGLE GWAS suggested that this signal is explained by the known functional *FLG* mutations (Paternoster et al. 2015), a previous study has suggested that there may be an independent signal in markers surrounding the *HRNR* gene, and so this should be considered in future work on this locus (Esparza-Gordillo et al. 2009).

Locus 1q21.3 - b

At this locus we tested 104 genes within the 3 Mb interval (Figure 1 & Dataset S1). Interleukin 6 receptor (*IL6R)* had the highest prioritization score – 743 (62% of top 10 cumulative score; Table 1 & Figure 1A), with the next best gene being *UBE2Q1*- score of 93 (only additional 8% of top 10 cumulative score). The high prioritization of *IL6R* by the pipeline is mostly driven by colocalization (Figure 2) in whole blood in eQTLGen and GTex (coloc posterior probability of colocalization (PPH4) = 63%-88% and TWAS *p*-value = 5 x 10^{-6}), in lipopolysaccharide-/muramyl dipeptide- primed monocytes in Kim-Hellmuth et al. (PPH4 = 75%-77%) and sun-unexposed skin in GTEx (PPH4 = 64%) (Table S3). Possible placement of many locus interval SNPs within an enhancer of the gene is highlighted by HiC interaction data in human embryonic stem cells, whole blood, CD34+ hematopoietic cells and lymphoblastoid cell lines and epidermal stem cells along with keratinocytes (Rinaldi et al. 2016). Previously, *IL6R* was also implicated as the potential causal gene at this locus in the EAGLE GWAS (Paternoster et al. 2015) as one of the variants in the MANTRA-based credible set of 23 associated variants, rs2228145, represents a missense (Asp358Ala) mutation in the gene (Esparza-Gordillo et al. 2013). This variant is ranked 3rd and is associated with blood serum levels of IL6R protein as well as transcript (Emilsson et al. 2018). However, the actual action of rs2228145 is to shift IL6R protein from membrane-bound state to soluble cell fraction, or in other words, from the anti-inflammatory classical signalling pathway to pro-inflammatory trans-signalling (Garbers et al. 2014). Mendelian randomization analyses indicated increased soluble IL6R levels as causal for higher AD (and asthma) risk and decreased soluble IL6R levels indicative of increased rheumatoid arthritis (RA) risk (Rosa et al. 2019). This is in agreement with the Asp358Ala mutation being protective towards RA and increasing risk in AD (Inshaw et al. 2018). However, in our colocalization analyses the AD risk allele is linked to overall reduction in *IL6R* transcript levels, likely due to the complex balance of IL6 classical and trans-signalling. A range of anti-IL6 biologics are in clinical use and development for inflammatory diseases, including tocilizumab, which inhibits both soluble and membrane-bound IL6R, and is approved for treatment of RA and juvenile idiopathic arthritis (Kalamaha et al. 2019). However, AD adverse events have been reported for in tocilizumab trials (Lin et al. 2019), consistent with the opposing effects of this GWAS locus on RA and AD.

Locus 2p13.3

Altogether, 35 potential candidate genes were tested at this locus within 3 Mbp centred around index SNP rs112111458 (Dataset S1). Among the top 3 ranked genes, *CD207* clearly stood out with the score of 272 (45% of the top 10 total, Table 1) compared to 2nd and 3rd ranked *CLEC4F* and *VAX2* (scores of 62 (10%) and 56 (9%), respectively). Our analysis showed strong colocalization with the protective allele leading to reduction of gene expression in the skin in TwinsUK RNA-Seq data, Sun-exposed and unexposed skin in GTEx, as well as transformed fibroblasts according to TWAS analysis (Table S3). No colocalization was detected for the other genes in the locus, which makes *CD207* a particularly promising candidate.

The original GWAS analysis in Paternoster et al. (2015) proposed that *CD207* is the likely candidate gene behind the locus due to colocalization of its microarray-derived eQTLs in TwinsUK (MuTHER) skin samples, CD207's specific expression in the skin Langerhans cells and the cells' abnormal phenotype presenting in the mouse knockout model. *CD207's* protein product langerin marks Langerhans cells resident in the stratified squamous epithelium of the skin and mucosal tissues (Zhang and Luo 2015)

Locus 2q12.1

This locus consists of two independent signals represented by rs6419573 and rs3917265 lead SNPs (Paternoster et al. 2015). Altogether, we considered 46 genes located within the 3 Mbp interval of each SNP (Figure 1B & Dataset S1). As mentioned earlier, the highest scores were obtained by *Interleukin-18 receptor 1 - IL18R1* (score=1384, Table 1) and *Interleukin-18 receptor accessory protein 1 - IL18RAP* (score=1341) which together account for 77% of the top 10 cumulative score at the locus, and so are likely to represent the causal genes behind the two signals. *IL18R1* and *IL18RAP*'s roles are supported by abundant expression colocalization evidence in the whole blood in GTEx (*IL18R1*: PPH4 = 51%-80% and p-value = 7 x 10^{-6}; *IL18RAP*: PPH4 = 96%-99% and p-value = 4 x 10^{-12}) and immune cell types in CEDAR, TwinsUK and Kim-Hellmuth *et al.* (*IL18R1*: PPH4 = 55%-86% and p-value = 2 x 10^{-5}, *IL18RAP*: PPH4 = 55%-97% and p-values = 5 x 10^{-5} to 6 x 10^{-8}), as well as skin in TwinsUK for *IL18R1* only (p-value = 1 x 10^{-4}; Figure 2 & Table S3). The direction of effect indicates increased expression of both genes for AD risk alleles, with one exception in GTEx whole blood for *IL18RAP* (Table S3). Interestingly, opposing expression effects of the same allele in a variant has previously been reported in monocytes and whole blood for *IL18RAP* (Kim et al. 2014). SNPs in LD with the lead SNP at the three top-ranked genes overlapped whole blood mQTL SNPs in the GoDMC study (Min et al. 2020), likely influencing the methylation of promoters of those genes. Differential gene expression (DGE) consistently shows significant upregulation of *IL18RAP* (Ewald et al. 2015) and *IL18R1* (Winge et al. 2011; Wittmann et al. 2005) in the skin of AD patients (Dataset S2). Both genes were put forward as candidate genes in the EAGLE GWAS annotation, as the credible set variants span both genes and individual variants linked to *IL18R1* eQTLs in TwinsUK skin microarray data, but previously there was little evidence for colocalization.

In general, cytokine IL-18, in the formation of whose receptor complex both IL18RAP and IL18R1 participate, has been shown to elicit significant immune gene expression change in keratinocytes from AD lesions (Wittmann et al. 2005). *IL18R1* and *IL18RAP* are both involved in T-cell, especially T helper cell signalling and activation of the NFkB-pathway. IL18R1 and IL18RAP mediate IL-18-dependent signal transduction, which is of special importance in Th1 response. Thus, it would not be surprising if one variant affected the expression of the whole gene cluster, and that has indeed been shown to be the case with SNP rs917997 associated with IBD and coeliac disease (Hedl et al. 2014); and ranked 8th among the two signals. A variant situated 15 kbp away from rs917997 - rs990171, again associated with celiac disease and lymphocyte counts (Milano et al. 2016), is ranked as second-best among the two signals in the locus (Dataset S1). There is strong pQTL support for association of rs990171 with IL18R and IL1RL1 resulting in increased protein availability (Suhre et al. 2017) (Dataset S2). In addition, rs990171 overlaps allele-specific eQTLs for IL18RAP and acetylation hQTLs in neutrophils (Chen et al. 2016) appearing during Th1 polarization (Soskic et al. 2019).

Locus 2q37.1

This locus was only discovered in the original GWAS after MAGENTA gene set enrichment analysis and was later revealed as a shared locus for asthma, allergy and eczema (Ferreira et al. 2017). Twenty candidate genes within 3 Mbp window of lead SNP rs1057258 were analysed (Dataset S1). The pipeline strongly prioritized the proximal gene of inositol polyphosphate-5-phosphatase D (*INPP5D*) with the score of 296 (57% of total top 10 score, Table 1), with the second and third best genes - *ATG16L1*, *RN7SL32P* - scoring only 106 (20%) and 29 (6%) respectively.

Colocalization evidence for *INPP5D* is tenuous, with posterior probability of shared causal GWAS and eQTL variant (PPH4) in monocytes in Blueprint estimated at 52% (Table S3) with the protective allele conferring reduced expression. The gene is upregulated in *FLG* mutation-harbouring eczema patients compared to negative controls (Winge et al. 2011), while downregulated in IBD, correlating there with reduced T CD4+ cell numbers (Fernandes et al. 2018). PrixFixe network prioritization algorithm selected *INPP5D* as the top candidate for the locus (Dataset S2). In addition, multiple exon-level eQTLs overlap SNPs at the locus, in agreement with existence of gene isoforms with internal promoters and displaying altered functionalities (Nguyen et al. 2011). *INPP5D* was also proposed as the candidate gene at the locus in the original EAGLE GWAS (Paternoster et al. 2015) due to the location of the lead SNP, involvement of the gene in cytokine signalling and knockout mouse phenotype.

rs1057258, the lead and top ranked SNP at the locus (score=101, Dataset S1) was also identified as lead SNP in the joint atopy GWAS by Ferreira et al. (2017) and associated with Alzheimer's disease progression (Wang et al. 2015a). The variant is located in the 3' UTR region of the *INPP5D* gene and may contribute to its regulation. Another top *INPP5D* SNP with potential causal role is 2nd ranked (score=93) intronic rs6740918 and 3rd ranked (score=69) predicted missense SNP rs9247 (His>Asn/Tyr). rs9247 has been picked up as the top SNP for the locus in a new integrative GWAS method – FINDOR, which incorporates variant functional enrichment information into association detection (Kichaev et al. 2019). The three variants: rs6740918, rs1057258 and rs9247 show histone modifications indicative of transcription in a variety of blood cell types (Roadmap Epigenomics Consortium et al. 2015) and are strong *INPP5D* eQTLs in naïve and induced monocytes (Fairfax et al. 2014; Ishigaki et al. 2017), and additionally neutrophils for the two last SNPs (Chen et al. 2016) (Dataset S2).

INPP5D, also known as SHIP-1, is a general inhibitor of activation of the signalling cascades leading to immune cell survival and division, including lymphocyte activation (Lorenz 2009; Zhang et al. 2000). The gene's signalling is also implicated in regulating neutrophil chemotaxis, macrophage activation, CD16-induced cytotoxicity in NK cells, among others.

Locus 4q27

The locus contains two independent signals, but we did not detect any differences in top candidate genes. Out of 132 genes contained within the 3 Mbp interval around index SNPs (Dataset S1), in each case we prioritize *KIAA1109* (score=220, 35% top 10 score), followed by *BBS12* (score=112), *TRPC3* (score=100), contributing 18% and 16% of the top 10 score, respectively.

Individual SNPs in the locus associate with KIAA1109 expression in the whole blood (Zhernakova et al. 2017) and T cells (Ishigaki et al. 2018), but no shared causal signal between GWAS and eQTL emerged in colocalization analyses (Dataset S2). While *KIAA1109*, in whose intron the lead variant in the GWAS study is localized, was ranked as first, only *BBS12* (Bardet-Biedl syndrome 12) gene had any colocalization evidence - in lymphoblastoid cell lines (LCL) in TwinsUK, with the risk allele conferring reduced gene expression (PPH4 = 91%, Table S3). Despite the gene being closest to an enhancer active in epidermal stem cells and keratinocytes (Rinaldi et al. 2016), no skin eQTLs were observed for *BBS12*.

KIAA1109's function has not been well characterized but missense mutations in the gene cause abnormal brain development and arthogryposis (curving of joints) in humans (Gueneau et al. 2018). *BBS12* encodes a chaperone, part of the chaperonin-containing T-complex (TRiC) assisting with protein folding and through that role mediating BBSome complex assembly, whose role is export of small vesicles to the cilia (Seo et al. 2010)

Locus 5p13.2

We considered 41 potential candidate genes situated within the 3 Mbp window centred on index SNP for this locus (Figure 1C & Dataset S1). Most of the cumulative score was assigned to *interleukin-7 receptor subunit alpha - IL7R* (score=965, which contributes to 65% of top 10 cumulative score, Table 1), followed by *SPEF2* (score=203 and a further 14%). While our GWAS results do not directly colocalize with any eQTLs for the gene (in datasets where this could be tested), there are multiple eQTL associations for SNPs in LD with the index SNP: in whole blood (Bonder et al. 2017; Zhernakova et al. 2017), CD4+ T cells (Chen et al. 2016; Ishigaki et al. 2017; Kasela et al. 2017), macrophages (Nedelec et al. 2016) and monocytes (Fairfax et al. 2014) and pQTLs in whole blood (Emilsson et al. 2018; Suhre et al. 2017) (Figure 2, Dataset S2) as well as promoter-enhancer interactions in human embryonic stem cells (Freire-Pritchett et al. 2017), CD34+ hematopoietic cells (Mifsud et al. 2015), naïve T regulatory cells and T helper 17 cells (Mumbach et al. 2017) (Figure 2, Dataset S2). *IL7R* is among the genes found to be upregulated in the skin in eczema patients in a meta-analysis(Ewald et al. 2015). Our results confirm initial prioritization of *IL7R* in the GWAS (Paternoster et al. 2015), which was supported by 18 credible set variants spanning the gene, including one non-synonymous (Thr>Ile) variant, rs6897932. Rs6897932 was ranked as the 8th most likely causal variant. The variant affects splicing of the *IL7R* transcript, with the minor C allele, the risk allele for multiple sclerosis (MS), favouring secreted over surface isoform of the protein. Elevated levels of secreted isoform exacerbate symptoms of MS in animal model (Al-mossawi et al. 2018; Galarza-Muñoz et al. 2017). In common with opposite effects seen in AD compared to autoimmune diseases, the minor allele is protective in eczema (Inshaw et al. 2018). *IL7R* is part of the thymic stromal lymphopoietin (TSLP) receptor/IL-7/IL7R axis required for correct lymphocyte maturation, especially of Th2 lymphocytes of interest in AD, with overexpression associated with acute lymphoblastic leukaemia (Gianfelici et al. 2018), whereas recessive mutations in the gene resulting in reduction of gene expression is seen in severe combined immunodeficiency (SCID) patients (Puel et al. 1998).

Locus 5q31.1

Our analysis lends support to two independent signals being present at this locus, which has been a GWAS hit in multiple immune-mediated diseases (Bowes et al. 2015) (psoriatic arthritis, juvenile idiopathic arthritis, IBD and asthma) and similarly hosts two independent signals in psoriatic arthritis. The first one, represented by the primary lead SNP rs12188917 and labelled "5q31.1 - a" here, is located in the intron of *TH2LCRR*, whereas the secondary signal (labelled 5q31.1 - b) lead SNP rs4705962 is an intronic variant of *KIF3A*. Original analysis presented in Paternoster et al. (2015)(Paternoster et al. 2015) suggests *RAD50* and *IL5* as the top likely targets due to overlap with eQTLs in both and cytokine function of the latter, which is at odds with our ranking.

Locus 5q31.1 - a

In total, 65 genes were found within the 3 Mbp window around index SNP rs12188917 (Dataset S1). Best ranked gene was *SLC22A5* with the score of 461 (Table 1), however that only constituted 35% of top 10 total score at the locus. Second-ranked gene *IRF1* scored 303 (23%) and 3rd ranked gene *RAD50* scored 122 (9%).

The gene ranked highest at the locus, *SLC22A5* (solute carrier family 22 member 5) displayed the strongest colocalization to be in Sun exposed-skin in GTEx (PPH4 = 94%; Table S3), but less supported colocalization was also observed in CD8+ T cells (CEDAR, 75%), monocytes (Blueprint, 77%) and whole blood (GTEx, 58.6%), with the risk allele resulting in reduction in gene expression, which agrees with the reduction of expression seen in eczema patients(Cole et al. 2014) (Dataset S2). *SLC22A5* was also the strongest eQTL candidate in CD8+ T cells and CD4+ T cells for the locus in psoriatic arthritis (Bowes et al. 2015) and in CD4+ T cells, CD15+ T cells in IBD. For 2nd best gene *IRF1* (interferon regulatory factor 1), colocalization evidence in the skin and activated monocytes is only moderate (PPH4 = 63%-64%, with increased expression in the skin associated with the AD risk allele – no direction reported for monocytes), and individual variant lookups in the interval around index SNP reveal IRF1 eQTLs only in the immune cells(Fairfax et al. 2014; Ishigaki et al. 2017; Nedelec et al. 2016; Zhernakova et al. 2017) (Dataset S2). Lastly, *RAD50* (RAD50 double strand break repair protein) is the only gene among the top 3 with colocalization evidence both from coloc and TWAS, and specific to the skin (GTEx Sun-exposed and unexposed, PPH4 = 58%-81%, *p*-value = 1×10^{-10}-7×10^{-10}; Table S3). The eczema risk allele leads to the reduction in the expression of this gene.

SLC22A5 represents a novel candidate gene at this locus (not considered in the previous EAGLE GWAS locus annotation). It is an organic cation transporter, specialized in carnitine uptake. Especially of interest in the case of AD, inflammation increases epithelial SLC22A5 expression, presumably as a downstream effect of action of pro-inflammatory cytokines such as IFN-γ(Console et al. 2018). *SLC22A5's* neighbouring paralog *SLC22A4*, also with carnitine transport function, is likely co-regulated with very similar blood cell type eQTL profile across the investigated studies (Dataset S2) and colocalization detected also in whole blood (eQTLGen, PPH4 = 99%) and CEDAR monocytes (*p*-value of 1 x 10^{-5}; Table S3), and likewise with risk allele leading to decreased expression. The gene's polymorphisms have similarly been implicated in RA and IBD (Fisher et al. 2006; Tokuhiro et al. 2003), but extensive LD in the region prevented further fine-mapping so-far.

IRF1 is worth mentioning due to its role as a master regulator of innate and acquired immune response, including activation and repression of interferon-inducible genes. This transcription factor binds to *IL4* promoter, thus promoting Th1 immunity, which is dampened in most of AD patients who displayreduced IRF1 levels (Gros et al. 2011). Furthermore, mouse knockout mutants do not mount Th1 response on infection by Leishmania, but instead undergo Th2 differentiation, in line with Th2 response dominance in AD patients.

Locus 5q31.1 - b

At the 5q31.1 - b locus we tested 48 genes within the 3 Mbp interval of the index SNP (Dataset S1). Ranking at this locus showed comparable score for the two top genes: *KIF3A* (score=249, Table 1) and *SLC22A5* (score=247) – i.e. the top ranked gene at the other signal in the 5q31.1 locus; each contributing 23% of the total top 10 score. A further 13% was contributed by the third ranked gene *PDLIM4* (score=142). We observed particularly strong *KIF3A* colocalization evidence in GTEx in the skin (sun-exposed and unexposed PPH4 = 96% and 95%; Table S3) and fibroblasts (*p*-value=2 x 10^{-11}), while *PDLIM4* colocalized well in LCL using both coloc (PPH4 = 91%) and TWAS (*p*-value=2 x 10^{-5}). The protective allele for AD was associated with increase in *KIF3A* and reduction in *PDLIM4* expression.

As the KIF3A protein provides the motor subunit to kinesin-2, it plays an important role in cilia production and motility, and its differential expression in nasal epithelial cells suggests roles in allergen clearance in asthma (Kim et al. 2011). This hypothesis was supported by asthma-like phenotype of *kif3a* mouse knockouts showing Th2-mediated pulmonary inflammation (Giridhar et al. 2016). Analogously to knockouts in the airway epithelial cells and consistent with direction of effect for risk allele seen in expression colocalization, mouse knockouts of the gene in the skin suffer from epidermal barrier dysfunction involving increased epidermal thickness and abnormal expression of filaggrin and claudin-1 (Stevens et al. 2020). They are also more prone to acquire AD following cutaneous allergen exposure. In humans, an intronic variant (rs12186803) in *KIF3A* has been shown to interact with allergens to increase risk for asthma in high-risk paediatric groups with and without AD (Johansson et al. 2019). Finally, *PDLIM4* (PDZ and LIM domain 4) is a gene promoting actin bundling and re-organization of the actin cytoskeleton (Guryanova et al. 2011).

Locus 6p21.32 and 6p21.33

The two loci positioned in the major histocompatibility region (MHC) represent difficult prediction targets due to notoriously intractable LD patterns in the region, which is why variant prioritization results are not presented. That fact along with high gene density - 106 and 117 genes in the 3 Mbp intervals around index SNP in the 6p21.32 and 6p21.33 loci, respectively (Dataset S1) - makes it also less likely that our top 3 prioritized genes will contain the true genes associated with eczema. Looking at colocalization results alone (Table S3), eQTLs for 6 and 9 genes at 6p21.32 and 6p21.33, accordingly, are found to show colocalization with the eczema GWAS.

At the 6p21.32 locus, we prioritize various human leukocyte antigen (HLA) genes in the top 8 ranked genes (Dataset S1); they all encode HLA class II alpha or beta chain paralogues. The top hit *HLA-DRA* has a score of 1405 which corresponds to 30% of the top 10 score, the next two best ranked genes each contribute less than half of that score: HLA-DQB1 (689 - 14%) and HLA-DRB1 (566 - 13%). Interestingly, the third ranked HLA-DRB1 has been previously hypothesized as the causal gene at the locus due to the strong association of the classical HLA allele HLA-DRB1*0701 (Weidinger et al. 2013).

The top 3 hits at the 6p21.33 locus are poorly distinguished, with scores ranging from 173 for the first ranked *HSPA1B*, through 165 for the second ranked *HCG27* to 152 for *CSNK2B.* Heat shock protein family A (Hsp70) member 1B (*HSPA1B*) colocalizes in the following immune cell types in the CEDAR dataset: CD19+ B cells (PPH4 = 93%), CD14+ monocytes (PPH4 = 99%), CD15+ T cells (PPH4 = 85%) and CD4+ T cells (PPH4 = 75%) with the protective allele correlated with reduction in gene expression. *HSPA1B* is a negative regulator of endoplasmic reticulum stress-induced apoptosis(Gupta et al. 2010). The third hit at the 6p21.33 locus, *CSNK2B* (casein kinase 2 beta) also strongly colocalizes in the CEDAR cohort: in platelets, CD14+ monocytes (PPH4 = 99.7%), CD8+ T cells (PPH4 = 99.4%), CD19+ B cells (PPH4= 99.8%) with the AD protective allele associated with reduced expression. This ER- and Golgi-targeted protein kinase has previously been associated with

schizophrenia(Yang et al. 2018) and SLE in GWAS studies (Milano et al. 2016) (Dataset S2). While the index SNP for the 6p21.33 locus is located in the intron of *MICB* (MHC class I polypeptide-related sequence B) gene, we found it to be linked to the locus only through strong colocalization in the granulocytes in the CEDAR dataset (PPH4 = 98%), where the protective AD allele corresponds to increased expression. The original Paternoster et al. (2015) analysis prioritized MICB because of location of credible interval SNPs in the gene and differential expression in eczema patients.

Locus 8q21.13

No gene is highly prioritized at this locus out of the 31 tested within the 3 Mbp window of index SNP (Dataset S1), with the top spot occupied by *ZBTB10* with a low score of 192 (41% of total top 10 score, Table 1), followed by *TPD52* and *PAG1* (scores of 70 and 69, respectively, each contributing 15% of total top 10 score). *ZBTB10* (zinc finger and BTB domain-containing protein 10) features a suggestive and sole colocalization hit at the locus, in TwinsUK LCL (PPH4 = 82%; Table S3) with the risk allele associated with reduced expression. Additionally, ZBTB10 is prioritized by regfm in CD8+ T cells, and the PrixFixe network enrichment method (Dataset S2). The second gene worth considering at the locus is third-ranked *PAG1* (phosphoprotein membrane anchor with glycosphingolipid microdomains 1). The gene is upregulated in *FLG* mutation-harbouring eczema patients' skin (Winge et al. 2011), is prioritized in the regfm pipeline in the thymus, and it neighbours enhancers active in epidermal stem cells and keratinocytes (Rinaldi et al. 2016) (Dataset S2).

Paternoster et al. (2015) also tentatively prioritize *ZBTB10* as the top candidate gene due to its function as a repressor of Sp1, a transcription factor regulating many immune-related genes(Ferreira et al. 2014). However, ZBTB10 transcripts were not available in the eQTL analysis of that study and so no eQTL evidence was previously presented. More recently, it has been shown that ZBTB10 is involved in alternative telomere lengthening mechanism characteristic of tumour cells(Bluhm et al. 2019). We believe PAG1 is also a promising candidate as the gene's overexpression has previously been linked to allergic disease. Vicente et al. 2015 demonstrated that rs7009110, a lead variant at the GWAS locus predisposing to allergy as well as lead variant in the previous AD GWAS (Paternoster et al. 2011), and ranked 9th at the current locus, is associated with *PAG1* expression in LCL in the GEUVADIS dataset. Regulatory regions were shown to act as *PAG1* transcriptional enhancers in an allele-specific fashion, with variant rs2370615's C risk allele (ranked 59th) determined as a substitution likely increasing *PAG1* transcriptional activation, and at the same time disrupting binding of Foxo3a, a negative regulator of lymphocyte activation in the NF-kB pathway. However, conflicting evidence, reviewed in Vicente et al. (2015), exists regarding PAG1's pro-inflammatory or anti-inflammatory role in T, B and mast cell activation.

Locus 10p15.1

We prioritize the *IL2RA* gene, neighbouring the lead SNP with moderate confidence at this locus. Out of 64 tested genes in the 3 Mbp neighbourhood (Dataset S1), *IL2RA* scored 333 which comprises 45% of the top 10 cumulative score (Table 1). Second-best ranked gene, *RBM17*, had 3x lower score (111 - 15%) and third best *PFKFB3* score of only 51 (7%). The locus contains GWAS-eQTL colocalizations with the top two prioritized genes in separate tissues. *IL2RA* (interleukin 2 receptor subunit alpha) shows strong evidence for colocalization (PPH4 = 98%) in eQTLGen's whole blood samples (Table S3), with the risk allele associated with increased expression. On the other hand, *RBM17* (RNA binding motif protein 17) colocalizes using TWAS (*p*-value = 4.2×10^{-6}) in the sun-exposed skin in GTEx, with the risk allele associated with decreased expression.

The original annotation in Paternoster et al. (2015) supported *IL15RA* over *IL2RA* as the candidate gene for this locus due to weak eQTL overlap evidence in whole blood (Bonder et al. 2017; Ishigaki et al. 2017; Pala et al. 2017; Zhernakova et al. 2017), monocytes (Fairfax et al. 2014) and CD4+ T cells (Schmiedel et al. 2018). There was no evidence of any overlap with IL2RA eQTL evidence previously presented. *IL15RA* only scores 46 (6% of the top 10 cumulative score at this locus) in our prioritization analysis (Dataset S1).

Regulation of IL2RA expression is complex, with at least 6 positive regulatory regions (PRRs) identified containing enhancers bound by ~8 transcription factors (Liao et al. 2013; Rosa et al. 2018). The top variant in the ranking, rs61839660 (score=141, Dataset S1), localizes to one such conserved intronic enhancer (Freire-Pritchett et al. 2017; Rainbow et al. 2017; Roadmap Epigenomics Consortium et al. 2015), contains a strong IL2RA eQTL (Bonder et al. 2017) predicted to be the causal variant linking AD GWAS and IL2RA expression (Võsa et al. 2018) in the blood, as well as likely to have a functional effect according to fathmm-XF (Rogers et al. 2018) (Dataset S2). This variant has also previously been convincingly fine-mapped as the causal variant behind the associations with T1D at this locus (Rainbow et al. 2017). It is of note that that the minor allele of rs61839660 is associated with protection from T1D, but risk of chronic inflammatory diseases, such as Crohn's (Simeonov et al. 2017), SLE, allergic disease and AD (Milano et al. 2016). The causality of rs61839660 has lately been supported with a new multinomial fine-mapping approach that borrows information from GWAS on 6 different

inflammatory diseases (Asimit et al. 2019). A recent landmark CRISPR activation study from Simeonov et al. (2017) present experimental evidence that rs61839660 regulates IL2RA through altering the function of an intronic enhancer, providing further evidence that this is the causal SNP behind the autoimmune signals at this locus. *Ho*wever, while the AD risk allele was shown to increase IL2RA expression in whole blood (Võsa et al. 2018) and CD4+ T memory cells (Rainbow et al. 2017), the allele appears to have a negative effect on gene's expression in T cells directly in response to antigen stimulus, delaying activation of IL2RA in the CRISPR study (Simeonov et al. 2017). Deletion of the entire enhancer harbouring the SNP and partially blocking IL-2 with antibody resulted in polarization of T cells towards proinflammatory Th17 cells, which the authors surmise would be the mechanism behind inflammatory phenotypes, such as Crohn's disease and psoriasis.

The *IL2RA* gene encodes a subunit of the CD25 receptor for interleukin 2, which resides in another AD GWAS locus - 4q27. While we do not prioritize *IL2* as a likely candidate gene for that locus, the signalling pathway in which the genes are involved is worth a closer look when investigating targets for AD susceptibility. IL-2 signalling both promotes T cell growth during the early phase of the immune response and cessation of effector T cell responses at the end of the process (Liao et al. 2013). The first mechanism involves a positive feedback loop, whereby naive CD4+ T cells are activated towards differentiation to Th1 and Th2 cells by IL-2 binding to IL2R which then induces IL-2 production and expression of IL2R by Th1 and Th2 cells. Dysregulation in either of the nodes in the IL2-IL2RA loop could increase the propensity to AD. The second mechanism involves a negative feedback loop whereby IL-2 promotes the T regulatory cell response which suppresses the effector T cell response initiated by the first mechanism, as well as Th17 differentiation through IL-2 sequestering (Busse et al. 2010). Missense and frameshift mutations in *IL2RA* mimic Immunodysregulation polyendocrinopathy enteropathy X-linked (IPEX) syndrome which is one of the monogenic diseases illuminating aspects of AD phenotype (Eyerich et al. 2018) due to featuring eczematous skin and immune dysregulation (Goudy et al. 2013).

Locus 10q21.2

Limited breadth of evidence contributes to gene prioritization at this locus, where we evaluate 39 genes within the 3 Mbp interval around index SNP (Dataset S1). While the score of the top ranked gene, *ADO* (2-aminoethanethiol dioxygenase), is 615 which makes up 61% of the top 10 gene cumulative score (Table 1), it is prioritized solely on the basis of association with the biggest number of individual variants in the whole blood(Bonder et al. 2017) and monocyte(Fairfax et al. 2014) eQTL studies rather than formal colocalization test as well as variants overlapping promoter regions interacting with putative enhancers in a variety of blood immune cells and associating with cardiac health phenotypes in GWAS Catalog (Dataset S2). The second and third ranked genes: *ZNF365* (zinc finger protein 365) and *EGR2* (early growth response 2) possess comparably lower scores of 101 and 90, i.e., each contributing 9-10% of cumulative score for top 10 ranked genes. Previous gene prioritization in Paternoster et al. (2015) supported *ZNF365* as the causal gene since lead SNP and credible set variants span the gene's intronic region, where an IgE GWAS signal was previously fine-mapped to (Pino-Yanes et al. 2015).

ADO encodes a thiol oxidase which catalyses the conversion of amino-terminal cysteine to cysteine sulfinic acid and shows homology to plant cystein oxidases involved in hypoxia response. In human cells, it has been shown to participate in signalling in the oxygen-regulated N-degron pathway of protein degradation as an enzymatic oxygen sensor (Masson et al. 2019).

ZNF365 (zinc finger protein 365) encodes multiple isoforms that have been variously linked to regulation of neurogenesis, maintenance of genome stability and uric acid secretion (Breuza et al. 2016). The signal in Crohn's disease GWAS locus has been fine-mapped to a nonsynonymous variant within *ZNF365* (Haritunians et al. 2011) and (based on position) the gene has been associated with several divergent phenotypes in the GWAS Catalog, ranging from breast-related, neural to chronic inflammatory disease and allergy.

Based on evidence derived solely from literature review, the third top candidate, *EGR2* appears most directly related to AD phenotype. EGR2 regulates the balance between clonal expansion and differentiation of CD4+ and CD8+ T cells (Miao et al. 2017), tipping it towards the former. Furthermore, EGR2 has been found to be a key repressor of Th17 cell pro-inflammatory program via inhibition of BATF activation (Miao et al. 2013). Gene knockout mice show increased production of IL-17 cytokines by naïve CD4+ T cells, differentiation into Th17 cells and reduced propensity for chronic autoimmune response in the central nervous system (Gao et al. 2018; Miao et al. 2013).

Locus 11p13

This locus stands out as having one clearly prioritized target gene - *PRR5L* (score=598, 79% of top 10 cumulative score; Table 1), with its final score nine times higher than the second-ranked gene, *TRAF6* (score=65 - only 9%). In total, we considered 15 genes located in the 3 Mbp region around index SNP (Figure 1D & Dataset S1). We could not tease apart the target behind the secondary signal represented by lead SNP rs12295535, with the same genes prioritized as for the primary signal represented by

rs2592555. rs2592555, the top prioritized variant at the locus (score=246, Dataset S1), is situated in the intron of *proline rich 5 like (PRR5L)*. We also note that second best SNP rs7925585 (score=132) for the primary signal, also positioned within the intron of *PRR5L*, was independently prioritized for eczema in FINDOR analysis (Kichaev et al. 2019). Strong expression colocalization was detected with the coloc method (Figure 2, Table S3) in: eQTLGen - whole blood (PPH4 = 95%); TwinsUK – skin (PPH4 = 91%), LCL (PPH4 = 98%); CEDAR - CD4+ T cells (PPH4 = 98%) with protective AD allele associated with increased expression. The gene's role was also supported using the network method PrixFixe (Dataset S2) and strongest methylation signal detected through mQTL overlap with locus interval SNPs in whole blood in the GoDMC study (Min et al. 2020). *PRR5L* was proposed as the candidate gene in the EAGLE GWAS due to position of the lead SNP in *PRR5L's* intron and *PRR5L* eQTL overlap with the credible set variants (Paternoster et al. 2015). However, there was previously very little evidence for colocalization of these signals.

PRR5L is part of the rapamycin complex 2 (mTORC2) which responds to extrinsic stimuli through cytoskeleton re-organization and cell migration (Gan et al. 2012). PRR5L specifically plays a role in regulation of fibroblast migration, and decreased expression of the gene conferred by the risk allele is predicted to lead to increase in fibroblast migration.

Locus 11q13.1

No single strongly prioritized candidate gene was highlighted among the 165 genes at this locus (Dataset S1). The gene with the highest score of 336, *CTSW*, contributed 23% of the total score for top 10 ranked genes (Table 1), while the 2nd ranked and 3rd ranked *OVOL1* and *EFEMP2* scored 236 (16%) and 168 (11%), accordingly. CTSW (cathepsin W) is differentially expressed in the skin between eczema patients and controls (Sääf et al. 2008; Winge et al. 2011), and numerous eQTLs in naïve (Kasela et al. 2017; Schmiedel et al. 2018) and stimulated T cells (Schmiedel et al. 2018) – including Th1, Th2, Th17, Treg, Treg memory cells, macrophages (Nedelec et al. 2016), and whole blood (Bonder et al. 2017; Zhernakova et al. 2017) are overlapped by variants in the locus interval, including evidence for allele-specific expression in T (Chen et al. 2016; Raj et al. 2014) and NK cells (Ishigaki et al. 2017) (Dataset S2). However, unlike the second-ranked gene, *OVOL1* (Ovo like transcriptional repressor 1), there was no evidence of colocalization in the datasets where this could be tested.

OVOL1 eQTLs boast highly confident colocalization with the AD signal in lymphocytes in GTEx (PPH4 = 99.9%; p-value = 2 x 10^{-18}; Table S3) as well as TwinsUK using both coloc (PPH4 = 99.9%) and TWAS (p-value = 8 x 10^{-18}) methods, with the AD risk allele correlated with downregulation of the gene. While *OVOL1* is not known to be differentially expressed in eczema, it shows differential expression between epidermal stem cells and differentiated keratinocytes with the index variant shown to lie in an enhancer contacting the gene's promoter (Rinaldi et al. 2016; Rubin et al. 2017) (Dataset S2). The third top candidate, *EFEMP2* (EGF-containing fibulin extracellular matrix protein 2) shows weak colocalization (PPH4 = 58%) in CD15+ granulocytes in CEDAR (Table S3), and independent variant lookups in the locus reveal eQTL overlap in whole blood (Bonder et al. 2017; Zhernakova et al. 2017), neutrophils (Chen et al. 2016), monocytes (Ishigaki et al. 2017), naive T cells (Kasela et al. 2017), macrophages (Nedelec et al. 2016) – similar pattern to that of *CTSW*. Likewise, *EFEMP2* is upregulated in keratinocytes versus epidermal stem cells (Rinaldi et al. 2016) but also in eczema patients (Sääf et al. 2008; Winge et al. 2011) (Dataset S2).

Prioritization of *OVOL1* as the candidate gene at the locus in Paternoster et al. (2015) relied on location of the index SNP rs10791824 within the intron of *OVOL1*, indirect evidence for regulatory role in AD-relevant cell types and mouse knockout phenotype. The mouse mutants are characterized by hyper-proliferation of keratinocytes, hair shaft abnormalities, delayed epithelial barrier formation and kidney cysts (Bult et al. 2008; Nair et al. 2006). The pipeline results supporting the rs10791824 variant as the most likely causal SNP (rank 1 with score of 968, Dataset S1) lend further support for potential importance of *OVOL1* at this locus. The variant shows high posterior probability (0.75-1) of being the causal SNP behind the eQTL and GWAS colocalization signal in all the datasets tested (Table S3), and likewise through fine-mapping in Finemap, JAM and Paintor programs (Dataset S2). Its role within an enhancer is supported by Roadmap classification (Roadmap Epigenomics Consortium et al. 2015), Blueprint hQTLs for K4ME1 and K27AC (Chen et al. 2016; Pelikan et al. 2018; Rinaldi et al. 2016) in blood and epidermal cell types, interaction with OVOL1 promoter in stem cell and B lymphocyte 3C data (Freire-Pritchett et al. 2017; Mifsud et al. 2015), localization within a FAIRE-seq peak in a study of epidermal disruption (Lander et al. 2017), overlap with CTCF motif ChIP-Seq peaks in LCL (Rao et al. 2014; Ziebarth et al. 2012), computational enhancer prediction models (Wang et al. 2018a) and moderate fitness consequences (Gulko et al. 2015). Additional lookups revealed the variant to be bound by lymphocyte-specific transcription factors, such as BATF and IRF4 in ENCODE Chip-Seq (Meno et al. 2018).

Literature evidence shows the first-ranked *CTSW* to be a papain family cysteine proteinase found exclusively inside the endoplasmic reticulum of natural killer and cytotoxic T-cells, yet not essential for cytotoxicity (Stoeckle et al. 2009). The gene is upregulated by IL-2, which together with IL2 receptor may be prioritized as candidate genes for AD GWAS loci 4q27 and 10p15.1, respectively. Second-ranked *OVOL1* induces upregulation of filaggrin and loricrin via aryl hydrocarbon receptor activation in human keratinocytes and through binding to c-myc promoter (Hirano et al. 2017; Tsuji et al. 2017), which provides a direct link to known atopic dermatitis gene targets. Further, nuclear translocation of OVOL1 is reduced in AD skin and normal skin treated with IL-4 (a cytokine in the Th2 pathway active in AD) providing more links of the gene to the disease. Finally, third-ranked *EFEMP2* is a member of the fibulin family of extracellular matrix proteins whose mutations cause collagen mis-assembly in humans and mice, especially affecting the skin (cutis laxa) and cardiovascular system (Papke et al. 2015).

Locus 11q13.5

Two genes are equally highly prioritized at this locus, out of 40 genes in the 3 Mbp interval around the index SNP (Dataset S1). They are *LRRC32* and *EMSY* (previously known as *C11orf30*) which score 545 and 521, correspondingly and together make up 84% of the top 10 genes at the locus (Table 1) – for comparison, the third ranked gene (*THAP12)* had a score of only 47. The 1st ranked gene *LRRC32* (leucine rich repeat containing 32) possesses the strongest colocalization evidence, albeit in one tissue – eQTLGen whole blood at 98% posterior probability of colocalization (Table S3), EMSY displays weaker colocalization signal in Blueprint's monocytes at 89%. In both cases, the AD risk allele confers reduced expression of the genes. Both genes have been shown as targets of differential enhancer interaction between naive T helper 17 cells and T regulatory cells (Mumbach et al. 2017) and individual variant eQTL lookups in whole blood - LRRC32 of healthy subjects (Bonder et al. 2017), while EMSY of patients with RA (Walsh et al. 2016a) (Dataset S2). However, only LRRC32 is linked to allele-specific expression QTLs in monocytes (Chen et al. 2016) and active enhancers in keratinocytes(Rinaldi et al. 2016). On the other hand, EMSY shows conflicting differential expression results in the skin of eczema patients: it is upregulated in homozygous filaggrin mutation AD patients in Winge *et al.* (2011)(Winge et al. 2011)(Winge et al. 2011), while downregulated in compound heterozygote FLG AD patients in Cole *et al.* (2014).

No evidence was available to prioritize either *LRRC32* or *EMSY* as the candidate gene in Paternoster et al. (2015), and all the credible set SNPs including the index SNP spanned the intergenic region between the two genes. Since then, both genes have been the focus of experimental validation (Manz et al. 2016),(Elias et al. 2019) which agrees with possible non-exclusive roles of *LRRC32* and *EMSY* in the immune T cells and skin, respectively. GARP's (protein encoded by *LRRC32*) role in AD and inflammatory disease in general has received convincing experimental support (Nousbeck and Irvine 2016). GARP is a cell surface receptor present on activated T regulatory cells, which is bound by latent transforming growth factor-β (TGF-β). Six rare missense mutations have been identified in AD patients in a targeted resequencing study with predicted coding changes affecting protein folding and post-translational modification (Manz et al. 2016). Characterization of one of the substitutions, the A407T variant, revealed reduced expression of the protein on the cell surface along with TGF-β latency-associated protein, and consequently reduced conversion of naïve T cells into T regulatory cells in the carriers. This agrees with GARP's role in promoting Treg activity established via shRNA and antibody knockdowns (Metelli et al. 2018), acting through its effect on TGF-β maturation and activation. More recently, GARP's expression has been shown to be downregulated in another allergic disease: eosinophilic esophagitis and upregulated in oesophageal epithelial cell lines on treatment with IL-13, supporting a putative role in regulating Th2 response (Kottyan et al. 2016).

While data available to us does not provide any colocalization evidence for significant role in the skin, possibly due to low expression range and hence low power for eQTL detection, common to transcriptional regulators, EMSY has recently been characterized as a potent regulator of skin barrier formation (Elias et al. 2019). siRNA knockdown of the gene in skin organotypic culture leads to increased *FLG* expression and enhanced barrier function, whereas overexpression in primary human keratinocytes leads to reduction in mRNA and protein markers of barrier formation. Lastly, EMSY has been shown to activate TSLP and CCL5 expression in eosinophilic esophagitis; both genes are known to contribute to inflammatory response also in atopic dermatitis (Al-Shobaili et al. 2017; Ewald et al. 2015; Ferreira et al. 2017; Fortugno et al. 2012; Hirota et al. 2012; Soumelis et al. 2002; Suárez-Fariñas et al. 2011; Zhu et al. 2018).

Locus 11q24.3

Amongst the 14 genes within the 3 Mbp window at this locus (Dataset S1), the pipeline prioritized *ETS1* with the score of 298 (75% of cumulative top 10 score, Table 1), in contrast to the scores of only 35 (9%) and 18 (5%) for the 2nd (*FLI1*) and 3rd (*APLP2)* ranked genes. We find modest colocalization support for *ETS1* (ETS proto-oncogene 1, transcription factor) in whole blood in eQTLGen (PPH4 = 48%, Table S3) and stimulated monocytes (PPH4 = 75%) with the risk allele correlating with increase in gene expression. Individual lookups linked variants in the locus interval to eQTLs in the whole blood (Bonder et al.

2017; Zhernakova et al. 2017), CD4+ T cells (Ishigaki et al. 2017) and neutrophils (Naranbhai et al. 2015) and promoter-enhancer interactions in embryonic stem cells for *ETS1* (Freire-Pritchett et al. 2017) (Dataset S2). In addition, *ETS1* showed consistent differential expression in AD patients regardless of *FLG* genotype. Weak colocalization evidence exists for *FLI1* (Fli-1 proto-oncogene, ETS transcription factor) in in Blueprint monocytes and neutrophils (54% and 46%, respectively, Dataset S2) suggesting increased expression to be associated with the AD risk allele, and individual eQTLs overlap variants in LD with focal SNP in monocytes *FLI1* (Ishigaki et al. 2017), whereas and in LCL and CD34+ hematopoietic cells (Mifsud et al. 2015) for promoter-enhancer interactions.

Initial candidate gene suggestion in Paternoster et al. (2015) was for *ETS1* due to its proximity (141 kbp downstream of *ETS1*) to the fine-mapped index SNP rs7127307 and prolific immunity-related functions of this transcription factor. Our analysis likewise highlights rs7127307 as being the likely causal variant at this locus (score=310, Dataset S1) through finemapping runs in Finemap, JAM and Paintor with posterior probability of 1, 0.7 and 0.31, respectively. The variant also shows the highest posterior probability of being the causal SNP for colocalization with *ETS1* eQTLs in stimulated monocytes and is situated in conserved genomic regulatory block of enhancers active only in immune cell types and fetal adrenal gland (Harmston et al. 2017).

ETS1 has been implicated in a wide variety of functions, where it was shown to act as co-activator; however in its role in immune dysregulation, it acts as a negative regulator of inflammatory Th17 cell responses and B cell differentiation but also a positive regulator of Th1 polarity (Grenningloh et al. 2005) acting through downregulation of the IL-6 pathway (Moisan et al. 2007) – specifically, a signalling subunit for the IL-6 receptor complex gp130 protein (see also locus 1q21.3). This hypothesized role was further confirmed by increased phosphorylation of STAT3 (see also locus 17q21.2) further downstream in the Th17 pathway and increased IL-17 and IL-22 production in *Ets1*-deficient CD4+ T cells. *ETS1* gene expression levels are decreased both in atopic dermatitis and skin autoimmune disease - SLE, and *Ets1* knockout mouse phenotype mimics aspects of molecular phenotypes of both diseases (Garrett-Sinha et al. 2016; Lee et al. 2019). Putative role of ETS1 in AD is also underlined by reduced IL-2 levels further affecting Th17/Treg imbalance (Mouly et al. 2010) and dampened expression of skin barrier genes: filaggrin, claudin-1 and loricrin in *Ets1*-deficient CD4+ T cell mouse model. On the other hand, overexpression of *ETS1* in keratinocytes of stratified epithelium in a mouse model (Nagarajan et al. 2010) resulted in profound abnormalities in the dermis driven by increased keratinocyte proliferation but reduced terminal differentiation: hyperplasticity, reduced granular layer, parakeratosis, compact stratum corneum and particularly interestingly for AD, increased permeability.

The 11q24 locus has been independently linked to other inflammatory diseases, amongst others, general atopy (Ferreira et al. 2017), RA (Galan et al. 2013), SLE (Bentham et al. 2015), psoriasis, IBD and celiac disease (Milano et al. 2016). Most of the variants map in the 3' region of the *ETS1,* but some are placed in the region between *ETS1* and *FLI1*, so *FLI1,* another transcription factors in the ETS family, should not be dismissed as a candidate gene. *FLI1* regulates collagen deposition by repressing collagen type 1 gene and is constitutively epigenetically silenced in systemic sclerosis (scleroderma) (Noda et al. 2014), which is key to recapitulating the disease symptoms, which centre on fibrosis of the skin and organs and Th2/Th17-skewed immune activation (Saigusa et al. 2017).

Locus 12q15

At this locus (identified in the MAGENTA gene-set analysis of the GWAS), our pipeline strongly prioritizes an unexpected target – *MDM1* (score=728, 70% of the top 10 cumulative score, Table 1) rather than the cytokine *IL22* immediately neighbouring the index SNP (score=99, ranked 2nd) or the immune modulator *IFNG*, whose assumed involvement led to the identification of this locus in the GWAS (score=57, ranked 3rd). Altogether 34 genes were considered at the locus, within the 3 Mbp window of the lead SNP rs2227483 (Dataset S1), and 3 out of 4 closest genes are involved in immune signalling (*IL22, IFNG and IL26*). However, the top prioritized gene is the fourth-closest gene, positioned 100 kbp away - *MDM1* (Mdm1 nuclear protein). Hundreds of individual significant whole blood (Bonder et al. 2017; Lloyd-Jones et al. 2017; Walsh et al. 2016a; Zhernakova et al. 2017) and immune cell subtypes (Chen et al. 2016; Fairfax et al. 2014; Fairfax et al. 2012; Ishigaki et al. 2017; Lappalainen et al. 2013; Raj et al. 2014; Schmiedel et al. 2018) eQTL lookups among interval variants, promoter-enhancer interactions target *MDM1* (Javierre et al. 2016a; Mifsud et al. 2015), and only promoter-enhancer interaction evidence (Javierre et al. 2016a; Mifsud et al. 2015; Rinaldi et al. 2016) is present for *IL22* (Dataset S2); similarly there is promoter-enhancer interaction evidence for *IFNG* but with additional prioritization by PrixFixe network method and modest colocalization support in activated monocytes (PPH4= 69%, direction of effect not reported) (Kim-Hellmuth et al. 2017).

MDM1 shows enhanced expression in multi-ciliated epithelial cells, where it localizes to centriole barrel and binds microtubules (Van de Mark et al. 2015). Naturally occurring mouse knockdown in the gene exhibits age-related retinal degeneration

(Chang et al. 2008). On the other hand, gene functional definitions and pathway positions rule in favour of the 2nd and 3rd ranked *IL22* (interleukin 22) and *IFNG* (interferon gamma, *IFN-γ*) as being more credible targets to influence AD phenotype. The expression of the Th22 cytokine IL-22 and IL-22$^+$CD8$^+$ T-cell frequency in adult skin lesions is positively correlated with AD disease severity characterized by increased epidermal hyperplasia and skin barrier impairment (Kim et al. 2016; Nograles et al. 2009; Suárez-Fariñas et al. 2011). IL-22 along with other cytokines in the family induces cell proliferation, migration and drives the expression of powerful inflammatory and antimicrobial molecules in keratinocytes, while at the same prevents their terminal differentiation (Nograles et al. 2008), in line with reduced expression of genes essential for cornified envelope formation, such as filaggrin. Improvement in AD severity in patients treated with IL-22 antibody (fezakinumab) gives credence to the idea that *IL-22* is the causal gene at the locus (Guttman-Yassky et al. 2018). In addition, *IFNG* could also be a plausible candidate gene, given the paramount role of this cytokine in innate and adaptive immunity. Similarly to IL-22, its levels positively correlate with SCORAD scores in intrinsic AD patients (Suárez-Fariñas et al. 2013). As a Th1 cytokine, IFN-γ plays an important function in intrinsic AD showing higher Th1 skewing (Kabashima-Kubo et al. 2012). IFN-γ production by Th1 cells in the dermis promotes apoptosis of keratinocytes which leads to formation of lesions (Akdis et al. 1999). At the same time, release of IFN-γ-inducible chemokines by apoptotic keratinocytes further aggravates T cell infiltration of the dermis in a positive feedback loop resulting in increased keratinocyte apoptosis and inflammation (Klunker et al. 2003). On the other end of the endotype spectrum, particularly low expression of IFN-γ and its receptor was found among a subset of AD patients susceptible to viral skin infection eczema herpeticum (Leung et al. 2011).

Locus 14q13.2

Out of 70 genes considered as causal at this locus (Figure 1E & Dataset S1), we find 2 of them to have comparably high scores - *Protein phosphatase 2 regulatory subunit B''Gamma* (*PPP2R3C*) with the score of 996 (31% of top 10 cumulative score, Table 1), and *KIAA0391* with the score of 814 (25% of top 10 cumulative score).

Three (PP2R3C, KIAA0391, *FAM177A1*) out of the four top-ranking genes at this locus display partial co-expression. The top-ranked candidate gene *PPP2R3C* shows colocalization (Figure 2, Table S3) in sun-exposed (PPH4 = 94%) and unexposed skin (PPH4 = 95%, *p*-value = 2 x 10^{-7}), whole blood in GTEx (PPH4 = 97%, *p*-value = 2 x 10^{-7}), CD15+ granulocytes (PPH4 = 96%) and colon (PPH4 = 96%) in CEDAR, and neutrophils (PPH4 = 93%) in Blueprint. The next best gene, *KIAA0391* recapitulates the colocalization in the skin in GTEx (sun exposed PPH4 = 97%, unexposed PPH4 = 96%), and LCL and individual immune cell types: LCL from TwinsUK (PPH4 = 94%, *p*-value = 3 x 10^{-9}), CD8+ T cells (PPH4 = 97%) and CD14+ monocytes from CEDAR cohort (PPH4 = 60%, *p*-value = 1 x 10^{-4}), in addition to the spleen in GTEx (PPH4 = 95% and *p*-value = 2 x 10^{-7}). Moreover, we find hundreds of individual variants in LD with the index SNP to overlap blood and skin eQTLs for those genes (Dataset S2). The orchestrated expression of these genes is underscored by their differential expression in atopic dermatitis skin: *PPP2R3C* is upregulated regardless of *FLG* genotype (Rodríguez et al. 2014; Winge et al. 2011) and *KIAA0391* is strongly downregulated in homozygous *FLG* mutation AD patients (Winge et al. 2011). Comparing that with our colocalization results, we see tissue-specific regulation for *PPP2R3C*, upregulation in the skin (in line with the differential expression results) but downregulation in the blood associated with the AD risk allele. For *KIAA0391*, the downregulated expression seen in AD patients is at odds with the eQTL result, where the AD risk allele is associated with increased expression; this conflict could indicate that *KIAA0391* is not an AD susceptibility gene. The original GWAS annotation (Paternoster et al. 2015) also suggested *PPP2R3C, KIAA0391* and *FAM177A1* as plausible causal genes, with the 47 credible interval SNPs scattered throughout the three genes and the lead SNP mapping to an intron within *PPP2R3C*; strong colocalization with TwinsUK microarray eQTLs in that paper was found only for *KIAA0391*.

Considering gene function, *PPP2R3C* is the most viable candidate. Targeted B-cell mouse knockout mutants show profound abnormalities in humoral immune response: reduced B cell proliferation, maturation, abnormal activation and small spleen (Xing et al. 2005). Similarly, loss of *PPP2R3C* in T cells results in atrophy of the thymus, decreased thymocyte abundance, especially of CD4+ and CD8+ double-positive thymocytes (Xing et al. 2008). Variants at the locus related to pleiotropic phenotype of chronic inflammatory diseases (AS, CD, psoriasis, primary sclerosing cholangitis, UC) (Ellinghaus et al. 2016) map to intron positions within *PPP2R3C*. Next, linking the gene's mutant phenotype established in mouse to human GWAS, it has been reported that decrease in lymphocyte counts (Astle et al. 2016) significantly correlates with the risk allele at rs2038255, the index variant in AD GWAS and our pipeline top prioritized variant (score=389, Dataset S1). *PPP2R3C* encodes a regulatory subunit of protein phosphatase 2A, known as G5PR, which associates with phosphatases PP2A, PP5, GANP protein and represses JNK and IKKβ (inhibitor of NF-κB) phosphorylation (Chiang et al. 2010). It is involved in regulating antigen-based B-cell and early T cell selection in the thymus promoting thymocyte and B cell survival. G5PR becomes upregulated in activated B cells and prevents B-cell receptor-mediated activation-induced cell death in B cells through suppression of late-phase JNK activation (Mahmudul et al. 2006). Overexpression results in the increase of production of non-specific B cells after immunization and

generation of autoantibodies in non-stimulated mice (Kitabatake et al. 2012). Therefore, *PPP2R3C* upregulation in the skin could be a contributing factor to autoimmune activation seen in a subset of severe AD patients and particularly directed against epidermal proteins (Altrichter et al. 2008).

KIAA0391 in contrast does not appear to be so directly functionally linked to the AD phenotype. It encodes a component of mitochondrial RNase P complex which catalyses the last step in pre-tRNA maturation process: removal of the tRNA 5' leader sequence (Li et al. 2015b).

Locus 14q32.32

Accounting for 55% of the cumulative score at the locus, *TNF receptor associated factor 3 (TRAF3)* is clearly prioritized among the 59 genes positioned within 3 Mbp of the index SNP at the locus (Figure 1F & Dataset S1). While TRAF3's score is 848, the second-ranked AMN scores only 281 (18%) (Table 1). *TRAF3* is the only gene at the locus with direct colocalization evidence (Table S3): in the whole blood in eQTLGen (PPH4 = 93%) and with lower confidence (PPH4 = 85%) in CD4+ T cells in Blueprint. In addition, many locus interval SNPs are possibly situated within an enhancer interacting the gene's promoter in human embryonic stem cells (Freire-Pritchett et al. 2017), whole blood (Javierre et al. 2016b), CD34+ hematopoietic cells and lymphoblastoid cell lines (Mifsud et al. 2015), naïve T regulatory cells and T helper 17 cells (Mumbach et al. 2017) and epidermal stem cells along with keratinocytes (Rinaldi et al. 2016) as shown by Hi-C data. In AD GWAS, risk alleles correlate with upregulated expression of TRAF3 and in IBD there is increased expression of the gene in inflamed intestinal mucosa (Shen et al. 2013). However, changes in expression have not been consistently observed in AD lesions relative to healthy skin (Dataset S2).

The original EAGLE GWAS annotation also presents *TRAF3* as the candidate gene at the locus due to the location of the index SNP, pathway enrichment in MAGENTA gene set analysis and mouse knockout phenotype, but no eQTL evidence (Paternoster et al. 2015). Two out of the 3 top prioritized SNPs at the locus (1st ranked rs79589176 and 3rd ranked rs12880641) as well as index SNP (rs7146581) are intronic, situated within the *TRAF3* gene, whereas the remaining top 3 SNP (2nd ranked rs71421262) is 2 kbp 5' upstream of *TRAF3*. *TRAF3*'s role in signal transduction in immunity is well-established, with early studies describing a serious imbalance in T cell composition in mouse model knockouts which eventually leads to their perinatal death (Xu et al. 1996). TRAF3 is a repressor of CD40- and B cell-activating factor-mediated signalling and limits homeostatic B cell survival (Bishop et al. 2018). *TRAF3* is also related to possible candidates at two other GWAS loci (*TRAF6* at 11p13 and *IL6R* at 1q21.3). TRAF6 positively regulates MAPK signalling and production of inflammatory cytokines and chemokines, whereas TRAF3 needs to be degradatively ubiqutinated during MyD88-dependent Toll-like receptor signalling to activate the JNK and p38 MAPK cascade (Tseng et al. 2010). TRAF3 exerts a negative effect on Th17-based inflammation by sequestering IL-17R, however, normally this is prevented by competitive binding of TRAF3 by NRD1. This allows formation of IL17R-Act1-TRAF6 complex and subsequent propagation of IL-17-induced signal down through MAPK and NF-κB pathways leading to production of pro-inflammatory molecules, including cytokine IL-6, whose receptor is prioritized in the AD GWAS at locus 1q21.3 (Ma et al. 2017).

Locus 16p13.13

Out of 143 considered genes at the locus within the 3 Mbp interval of index SNP (Dataset S1), two top genes are prioritized equally strongly– *DEXI* (score=376, Table 1) and *CLEC16A* (score=364). Their combined score contributes to 67% of the cumulative top 10 gene locus score and the third ranked gene *RMI2* contributes only a further 10% (score=108). Colocalization evidence at the locus is limited and prioritizes *DEXI* (dexamethasone-induced protein) and *CLEC16A* (C-type lectin domain containing 16A) in CEDAR's rectum tissue based on TWAS and modest support by coloc (PPH4= 73%), respectively. The protective AD allele confers increased expression of both genes. *DEXI* and *CLEC16A* are precisely the two genes with correlated gene expression across cell types that have been championed as the top candidate genes at this locus in association studies of inflammatory and autoimmune disease, such as T1D, MS, alopecia areata, SLE, asthma (Milano et al. 2016) (Dataset S2). Risk alleles of autoimmune disease-linked variants (T1D, MS) in the locus are associated with lower *DEXI* expression in monocytes, thymus and LCLs (Davison et al. 2012a), which mirrors the effect of protective AD allele conferring higher *DEXI* expression. *CLEC16A* is differentially expressed in eczema: it is highly downregulated in *FLG* mutant eczema skin patients relative to healthy controls (Winge et al. 2011). However, comparison of lesional vs non-lesional skin in AD patients reveals small upregulation of the gene(Rodríguez et al. 2014).

DEXI represents a novel candidate gene at this locus. The original AD GWAS annotation in the EAGLE paper (Paternoster et al. 2015) presented *CLEC16A* as the candidate gene at the locus due to location of the two SNPs from the fine-mapped credible set within the gene's intron and binding of STAT3 (see locus 17q21.2) in mammary epithelial cells as well as top RegulomeDB

score at the lead SNP (Boyle et al. 2012). We too prioritize the index SNP rs2041733 (ranked 1st, score of 102; Dataset S1) thanks to strong fine-mapping evidence in Finemap and JAM (Dataset S2) but also some evidence from eQTL colocalization (posterior probability of being a causal variant > 0.5). Regulatory function of the SNP is supported by overlap with a strong caQTL affecting regulatory DNA accessibility(Maurano et al. 2015) across Roadmap cells and strong hQTL for K4ME1 in monocytes (Chen et al. 2016) and skin cells (Rinaldi et al. 2016).

Direct looping interaction from lead SNP-harbouring *CLEC16A* intron to *DEXI* promoter has been shown with 3C in monocyte-like cell line, lung epithelium and LCL, suggesting that *CLEC16A* intron contains a *DEXI* enhancer (Davison et al. 2012b). Nevertheless, these variants are situated within intron 19 of *CLEC16A,* about 29 kbp away from the top AD SNPs (intron 23) so they could well be tagging an independent GWAS signal, as 3 independent signals at the locus have been suggested in MS (Zuvich et al. 2011). CRISPR-Cas9-disrupted *DEXI* expression precipitates early onset of diabetes in T1D mouse model and it has been postulated that DEXI impacts T1D risk through changes to host metabolites and microbiome (Davison et al. 2018). Specific targeting of beta cells in pancreas to knock down DEXI revealed reduction in interferon β expression followed by lower production of inflammatory chemokines on exposure to synthetic dsRNA and reverse in the case of DEX1 overexpression, pointing to roles in antiviral immune response (Dos Santos et al. 2019).

CLEC16A is a much better characterized gene with multifarious posited functions related to autophagy and secretion. Initially, it was identified as an endosomal protein promoting murine mitophagy by interacting with the Nrdp1 E3 ubiquitin ligase, in turn a binding partner of Parkin, the key regulator of mitophagy (Soleimanpour et al. 2014). Loss of *CLEC16A* in that context results in inefficient elimination of abnormal mitochondria through autophagy and predisposes to inflammation (Pandey et al. 2018). CLEC16A also likely negatively regulates starvation-induced autophagy (Tam et al. 2017). Next, knockdown of the gene in mice led to halving of the number of B cells and elevated IgM levels (Li et al. 2015a). CLEC16A functions also impact other lymphocytes: CLEC16A limits NK cytotoxicity (Pandey et al. 2019; Pandey et al. 2018) and fine-tunes thymic T cell selection by regulating thymic epithelial autophagy (Schuster et al. 2015). In general, the gene's expression has a negative impact on cell surface receptor expression and cytokine (such as interferon-γ) and chemokine release. Unlike in AD, where atopic dermatitis risk correlates with low expression of *CLEC16A* in the skin tissue, patients with autoimmune MS display increased *CLEC16A* expression in monocytic and dendritic cells (Van Luijn et al. 2015). Of importance in MS, CLEC16A positively regulates HLA-II antigen presentation pathway through participation in late endosomal biogenesis and trafficking via binding members of the dynein complex (Van Luijn et al. 2015).

Locus 17q21.2

Our pipeline was not able to provide much evidence for a single candidate gene at this locus, out of 147 genes within the 3 Mbp window of index SNP (Dataset S1). The top ranked gene *DHX58* scored only 254 (32% of the top 10 cumulative score, Table 1), while the 2nd, 3rd and 4th ranked *STAT3*, *RAB5C* and *CAVIN1* 101, 100 and 94, in that order, made up a further 37% of the cumulative score. Tentative eQTL-GWAS colocalization evidence is only present for *STAT3* (signal transducer and activator of transcription 3) at PPH4= 50% in Sun-exposed skin in GTEx (Table S3) and for the 4th-ranked *CAVIN1* (caveolae associated protein 1) gene, similarly in GTEX Sun-exposed skin (68%) but also in TwinsUK LCL (55%). In both cases, the risk allele is predicted to lead to decrease in gene expression. However, it is *DHX58* (DExH-box helicase 58) that occupies the first position in the ranking due to multiple individual eQTLs overlapping the locus interval: in whole blood (Bonder et al. 2017; Lloyd-Jones et al. 2017), allele-specific expression in monocytes (Fairfax et al. 2014) and T cells (Chen et al. 2016; Kasela et al. 2017), in the skin (Fagny et al. 2017), B cells and LCL (Ishigaki et al. 2017; Lappalainen et al. 2013), NK cells (Ishigaki et al. 2017), Tfh cells, Treg memory cells (Schmiedel et al. 2018) (Dataset S2). Fewer significant eQTLs in a smaller number of tissues were found for *STAT3*: whole blood (Lloyd-Jones et al. 2017), naive and induced monocytes (Fairfax et al. 2014), CD8+ T cells, NK cells(Ishigaki et al. 2017). Again, slightly different profile is seen for *RAB5C* (Ras-related protein Rab-5C), with eQTLs in: whole blood (Bonder et al. 2017), B cells, CD4+ and CD8+ T cells, monocytes (Ishigaki et al. 2017), macrophages (Nedelec et al. 2016), Tfh, Th17, Th2 cells (Schmiedel et al. 2018). Lastly, variants in the GWAS locus interval are also associated with the expression of *CAVIN1* (caveolae associated protein 1) in the whole blood (Bonder et al. 2017), CD4+ T cells (Ishigaki et al. 2017), and Th1 cells (Schmiedel et al. 2018). *CAVIN1* is the only gene of the four, where we could find evidence of differential expression in AD patients: strong downregulation in lesional skin of patients with FLG mutation relative to healthy controls (Winge et al. 2011).

The original Paternoster et al. (2015) post-GWAS annotation focused on *STAT3* since the lead SNP is located in the intron of the gene, and further support was provided by eQTL lookups in relevant tissues, evidence for regulatory function of SNPs in the credible set interval and background knowledge on STAT3 function. The index SNP rs12951971 comes up top in our ranking too

(score of 126, Dataset S1) as evidenced by high posterior probability of causality in Finemap and JAM runs and high effect mQTL mapping to STAT3. The second best SNP, rs4796791 (score=44) and also with intronic location in *STAT3*, is too supported by Finemap runs, overlaps a sentinel variant in a MS GWAS(Beecham et al. 2013) and strong effect STAT3 eQTLs in whole blood(Lloyd-Jones et al. 2017), monocytes(Raj et al. 2014) and mQTL in whole blood.

The top prioritized hit *DHX58* encodes the LGP2 protein is involved in regulating innate immune response to virus infection (Venkataraman et al. 2007). LGP2 negatively regulates IFN during two early stages in the induction of interferon production (Vitour and Meurs 2007) but has also a positive role in antiviral response through facilitating viral dsRNA recognition (Satoh et al. 2010). *STAT3*, our second ranked gene is a ubiquitous regulator and signal transducer in JAK-STAT signalling pathway, with many functions in immunity, including cancer and inflammatory skin disease, such as psoriasis and eczema (Calautti et al. 2018). Consequently, here we focus on summarising established associations of STAT3 with AD.

First of all, STAT3 is activated downstream of cytokines important for AD: IL-4, IL-13, IL-22, IL-23, IL-12, IL-6, IL-10 and IL-31 (Fridman et al. 2011; Lee et al. 2012). One example of activation of STAT3 by cytokines would be in terminal keratinocyte differentiation in normal skin barrier formation, where the IL-4/IL-13-JAK-STAT3 pathway has been shown to be the master regulator. STAT3 signalling is thought to contribute to TSLP-dependent downregulation of filaggrin and anti-microbial peptides in keratinocytes in AD (Cabanillas and Novak 2016; Kim et al. 2015; Lee et al. 2016). JAK-STAT3 signalling also plays a role in mediating pruritogenic effect of IL-31 in eczema (Furue et al. 2018). Other relevant functions of STAT3 in related diseases include: promotion of dermal fibrosis in scleroderma through increased collagen and fibronectin deposition in the extracellular matrix (Pedroza et al. 2018) and skewing towards the IL-23/IL-17 axis in psoriasis through STAT3-dependent induction of the IL-23 receptor, RORα, RORγ and IL-17 expression (Calautti et al. 2018; Hillmer et al. 2016; Miyoshi et al. 2011). Indeed, STAT3 is activated in lesions of psoriatic patients and a mouse mutant with keratinocyte-specific constitutive expression of activated STAT3 serves as a model of psoriasis (Sano et al. 2005).

On the other hand, our eQTL and colocalization analysis links the AD risk allele to reduced expression of *STAT3*. Loss-of-function mutations in *STAT3* result in autosomal dominant hyper-IgE syndrome (HIES) featuring high IgE levels and eczema-like skin lesions plagued by recurrent infections arising due to Th1/Th2 imbalance similar to seen in AD (Boos et al. 2014; Schimke et al. 2010). However, HIES subjects entirely lack IL-17-producing Th17 cells (Milner et al. 2008). Counter-intuitively, STAT3 serum levels and gene expression are elevated in early-onset paediatric eczema (Amano et al.) relative to late-onset adult eczema cases and correlate with epidermal barrier function (Brunner et al. 2018). This could partially be due to immune activation of early-onset acute eczema resembling that of psoriasis in terms of Th17 polarization (Brunner et al. 2018; Esaki et al. 2016).

The third-best prioritized gene, *RAB5C*, is primarily involved in controlling endocytosis, formation of adhesion foci and cell motility (Chen et al. 2014; Ulrich et al. 2005). The fourth-ranked gene *CAVIN1* is highly expressed in fibroblasts, adipocytes, epithelial and endothelial cells where it is necessary for formation of caveolae, specialized invaginations of the plasma membrane with roles in signal transduction, lipid transport and clathrin-independent endocytosis (Hill et al. 2008; Liu et al. 2008). Mice knockouts in the gene suffer from lipodystrophy and altered lung morphology (Ni et al. 2018), while frame-shift mutations in humans cause congenital generalized lipodystrophy type 4 with muscle rippling (Rajab et al. 2010). CAVIN1 participates in membrane repair mechanism by binding cholesterol exposed during injury and acting as a nucleation site for forming a membrane patch (Zhu et al. 2011). Of interest in AD, expression of *CAVIN1* has been linked to asthma and chronic inflammatory respiratory disease for its antifibrotic role as well as maintaining E-catherin-based cell-cell adhesion and therefore airway epithelium integrity which is diminished in scledorema patients and asthmatics (Hackett et al. 2013; Royce and Le Saux 2014), respectively. There is also evidence of role of post-translational modification of CAVIN1 on its role in asthma, with dephosphorylated CAVIN1 seemingly controlling the release of IL-33 and Th2 response in the challenge phase of mouse asthma model (Ni et al. 2018).

Locus 17q25.3

The 3 Mbp region around the lead SNP encompassed 41 genes which were included in our analysis (Dataset S1). The lead SNP is positioned within an intron of *PGS1*, the top prioritized gene (score=205, 46% of top 10 total score; Table 1), followed by the gene's neighbours: *DNAH17* (score=73) and *SOCS3* (score=52) which contributed 16% and 12% to the top 10 total score in the ranking. Colocalization with *PGS1* (Phosphatidylglycerophosphate synthase 1) eQTLs was detected in the skin (PPH4 = 95%) and fibroblasts (PPH4 = 98%) in GTEx, and suggestively so in LCL (PPH4 = 69%) in the TwinsUK cohort (Table S3), with the AD risk allele correlating with reduced expression. In addition, 385 significant eQTLs in the locus were also found by individual

variant lookups (Dataset S2). The two other top gene hits are differentially expressed in eczema patients: *DNAH17* (dynein axonemal heavy chain 17) is 2-6x downregulated (Cole et al. 2014; Winge et al. 2011) and *SOCS3* (suppressor of cytokine signaling 3) upregulated (Ewald et al. 2015). While there are 110 eQTLs associated with *DNAH17* in the region (Bonder et al. 2017; Fairfax et al. 2014; Zhernakova et al. 2017), only 27 (Ishigaki et al. 2017; Schmiedel et al. 2018) were detected for *SOCS3* despite chromatin conformation capture evidence for interactions between variants in the region and gene's promoter (Freire-Pritchett et al. 2017; Mifsud et al. 2015; Rinaldi et al. 2016), and numerous overlapping mQTLs.

The EAGLE GWAS annotation (Paternoster et al. 2015) postulated that *SOCS3* is the causal gene at the locus due to MAGENTA gene set enrichment results, the gene's regulation of cytokine signalling, case-control SNP haplotype association and differential expression in AD.

Despite eQTL-GWAS colocalization signal observed for *PGS1*, any possible link to AD is not immediately apparent for this enzyme catalysing the first step in production of cardiolipin (Kawasaki et al. 1999), an integral lipid component of the inner mitochondrial membrane (Shen et al. 2015). However, anticardiolipin antibodies are present in child patients with extrinsic AD (Szakos et al. 2004; Wenzel and Bieber 2004). The antibodies are reported also commonly in autoimmune diseases, e.g. in antiphospholipid syndrome (APS) or SLE. *DNAH17* is predicted to encode a chain of dynein motor protein implicated in cilia assembly and motility(Zariwala et al. 2007).

Only the third-ranked gene – *SOCS3*, despite low evidence for prioritization, appears to be directly involved in AD-related cellular processes. SOCS3 exerts a significant negative regulatory role in IL-1, IL-6, IL-12 and IL-23 cytokine signal transduction, among other regulatory functions relating to cytokines, hormones and growth factors (Yin et al. 2015). Loss of SOCS3 has severe consequences, with deletion resulting in embryonic lethality. Increased *SOCS3* expression in the gut has been observed in IBD patients where it correlated with inflammation severity (Li et al. 2010; Miyanaka et al. 2007). Similarly, for asthma and atopic dermatitis, *SOCS3* expression in CD3+ T cells positively correlates with disease pathology. Constitutive expression of *Socs3* in murine T cells increased Th2 skewing and enhanced airway hypersensitivity (Seki et al. 2003), and the reverse in conditional knockout mutants in T cells (Kinjyo et al. 2006). Keratinocyte-specific knockout of *Socs3* results in abnormal wound healing characterized by hyperproliferative epidermis and neutrophil infiltration (Zhu et al. 2008).

Locus 19p13.2

Our pipeline provides limited evidence for the top 3 prioritized genes at this locus, out of the 30 genes positioned within the 3 Mbp window around index SNP and included in the analysis (Dataset S1). The intergenic index SNP at the locus is situated between *ACTL9* (ranked 1st, score 115 and 41% of top 10 cumulative total; Table 1) and *ADAMTS10* (ranked 2nd, score 57 and 20% of top 10 cumulative total). The third ranked MAP2K7 scored only 34, 12% of top 10 cumulative total. While *ACTL9* (actin like 9) is promoted as the top hit, it is only due to numerous promoter-enhancer interactions (Freire-Pritchett et al. 2017; Javierre et al. 2016a; Mifsud et al. 2015) and mQTLs linked to this gene (Dataset S2). Second ranked *ADAMTS10* (ADAM metallopeptidase with thrombospondin type 1 motif 10) is downregulated in the skin of homozygous *FLG* mutation AD patients (Winge et al. 2011) and individual look-ups show 37 eQTL hits in the locus interval in whole blood, monocytes and T cells (Bonder et al. 2017; Gutierrez-Arcelus et al. 2015; Ishigaki et al. 2017; Zhernakova et al. 2017) . Third ranked gene, *MAP2K7* (mitogen-activated protein kinase 7) is the only gene with colocalization evidence at the locus (Table S3). Posterior probability of colocalization is 90% in CD4+ T cells in CEDAR with risk allele associated with reduction in expression, and the gene is also downregulated in homozygous and heterozygous *FLG* mutation AD patients (Winge et al. 2011), as well as on differentiation from epidermal stem cells to keratinocytes(Rinaldi et al. 2016) (Dataset S2). No clear candidate gene was presented in the Paternoster et al. (2015) AD GWAS annotation.

ACTL9 encodes an uncharacterized protein from the family of actin-related proteins. *ADAMTS10* belongs to a family of 19 secreted zinc-dependent metalloproteinases that participate in connective tissue remodelling. Murine knockouts in the gene show multiple abnormalities, relating to ciliary body, epidermal-dermal junction and skeletal muscle morphology (Bult et al. 2008; Mularczyk et al. 2018). In humans, mutations in the gene have been mapped to autosomal recessive Weill-Marchesani Syndrome (Dagoneau et al. 2004), characterized by generalized mesodermal dysplasia affecting bones, eyes and resulting in pachyderma (Faivre et al. 2003). Molecular characterization revealed that ADAMTS10 specifically helps in formation of fibrillin microfibrils in the extracellular matrix (Kutz et al. 2011) and thus promotes focal adhesions and epithelial cell-cell junction formation (Cain et al. 2016).

MAP2K7 is a kinase in the environmental stress-activated protein kinase/c-Jun N-terminal kinases (SAP/JNK) signalling pathway and relays signal in the JNK pathway triggered by proinflammatory cytokines, specifically IL1-α (Foltz et al. 1998; Tournier et al. 2001), crucial in skin inflammation and whose expression positively correlates with AD symptoms (Foltz et al. 1998). In dendritic cells within tumour microenvironment, MAP2K7 is positioned downstream of TGF-β (Min et al. 2012), which upregulates the expression of miR-27a that silences, among others, *MAP2K7*. This results in inhibition in expression of key pro-inflammatory cytokines and in parallel accumulation of T regulatory cells along with reduction in differentiation to Th1 and Th17 cells, suggesting dual role of JNK pathway kinases in regulating cytokine expression; however, the Treg/Th17 balance observed in *MAP2K7* knockouts is the direct opposite of the one seen in AD (Ma et al. 2014; Verhagen et al. 2006).

Locus 20q13.33

At this locus, we tested 109 potential candidate genes situated within the 3 Mbp interval around index SNP (Dataset S1). The three top-ranked genes are novel candidates which show consistent support in independent eQTL colocalization analyses in whole blood, immune cell types and skin (Table S3): *STMN3* (score=608, 27% of top 10 cumulative total; Table 1), *LIME1* (score=473, 21% of top 10 cumulative total), and *ARFRP1* (score=257, 12% of top 10 cumulative total).

STMN3 (stathmin 3) eQTLs from sun-exposed/unexposed skin (PPH4 = 99.2% in coloc and *p*-value of 2 x 10^{-8} in TWAS; protective allele correlating with increased expression; Table S3) and whole blood in GTEx (PPH4 = 98%, reduced expression), and Blueprint's monocytes (PPH4 = 91%, reduced expression) show strong evidence of colocalization with AD GWAS, and TwinsUK's skin shows slightly weaker evidence of colocalization (PPH4 = 87%, increased expression). In a similar way to *STMN3*, eQTLs in *LIME1* (Lck-interacting transmembrane adaptor 1) colocalize in GTEx sun-exposed/unexposed skin (PPH4 = 87% and PPH4 = 90%, protective allele correlating with increased expression), whole blood (PPH4 = 91%, increased expression), CEDAR CD4+ T cells (PPH4 = 92%, reduced expression), TwinsUK skin (PPH4 = 99.2%, increased expression) and eQTLGen whole blood (PPH4 = 89%, increased expression). Third ranked, *ARFRP1* (ADP ribosylation factor related protein 1) shows good evidence for colocalization in GTEx: whole blood (PPH4 = 88%, protective allele correlates with increased expression), fibroblasts (PPH4 = 83%, reduced expression) and in Blueprint neutrophils (PPH4 = 91%, increased expression).

This is the only locus, where we detected pQTL colocalization in the Sun *et al*. (2018) dataset – however, the posterior probability of colocalization for *TNFRSF6B* (TNF receptor superfamily member 6b, ranked 5th) was moderate at 72% (Table S3). The protective allele correlates with increased abundance of the protein product DcR3. Four additional genes showed eQTL colocalization in single tissues: *GMEB2* (glucocorticoid modulatory element binding protein 2) – ranked 4th, *SLC2A4RG* (SLC2A4 regulator) – ranked 7th, *UCKL1* (uridine-cytidine kinase 1 like 1) – ranked 13th and *RBBP8NL* (RBBP8 N-terminal like) – ranked 16th. Complex pattern of gene co-regulation at the locus does not make it easy to distil the likely GWAS target(s) but the three top prioritized genes along with *TNFRSF6B* offer the amplest, albeit differing across tissues, evidence of relationship with the AD protective variant. Similarly, the original GWAS analysis in Paternoster et al. (2015) did not provide a clear candidate gene for the locus, and mentions *TNFRSF6B*, *RTEL1*, *LIME1*, *SLC2A4RG* as potential targets.

STMN3 is a member of the stathmin-like family of proteins, which are involved in regulating microtubule dynamics and inhibit tubulin polymerization (Charbaut et al. 2001). STMN3, also known as SLIP, is especially intensively expressed in central nervous system and highly proliferative cancer cells, associated with neural cell growth (Zhang et al. 2015), as well as tumour cell survival and migration (Li et al. 2016; Ng et al. 2009; Zhang et al. 2015). Its knockdown in breast cancer cells causes loss of epithelial morphology, concomitant with reduced cadherin expression (Ng et al. 2009).

LIME1 is a poorly characterized transmembrane adaptor phosphoprotein that relays T cell receptor stimulation to downstream signalling pathways following its phosphorylation by the Src family kinases (Brdičková et al. 2003; Hur et al. 2003). The protein is highly and specifically expressed in nasal respiratory epithelium, followed by cytotoxic CD8+ T cells, helper CD4+ T cells, B-lymphocytes and NK cells (Fishilevich et al. 2016) where it moves to immunological synapse on contact with antigen-presenting cells and is later downregulated after T cell activation. Overexpression of the gene in T cells induces *IL-2* promoter activity (Hur et al. 2003), which connects the gene more closely to AD phenotype, because of the importance of Th2 response in AD development, further underlined by possible direct role for interleukin-2 (AD GWAS locus 4q27) and/or its receptor (AD GWAS locus 10p15.1) in mediating AD risk.

ARFRP1 is a small GTP-ase which is associated with the *trans*-Golgi, where it regulates intracellular protein trafficking, such as of E-catherin through the Golgi to plasma membrane and in particular, plays a key role in lipoprotein maturation and formation of lipid droplets in adipocytes (Hesse et al. 2014; Hommel et al. 2010; Shin et al. 2005; Zahn et al. 2008).

TNFRSF6B encodes a decoy protein DcR3 which binds to FAS ligand and LIGHT and neutralizes their action. Binding of DcR3 to FAS ligand, which is produced by activated T cells and NK cells and promotes cytotoxic killing of cells, antagonizes apoptosis of such cells (Pitti et al. 1998). In addition to inducing apoptosis, LIGHT promotes T cell activation and regulates airway

modelling in asthma patients (Doherty et al. 2011; Lin and Hsieh 2011). Independently of action on FASL and LIGHT, TNFRSF6B is thought to positively regulate dendritic cell differentiation and Th2 polarization. TNFRSF6B serum concentration is increased in inflammatory disease, including allergic disease: atopic dermatitis and asthma (Chen et al. 2004; Ellinghaus et al. 2013; Kowal et al. 2019; Lin and Hsieh 2011), which does not agree with the direction of effect seen in pQTLs, whereby a protective allele would lead to higher protein expression. A synonymous variant in the gene has been previously reported as a top SNP in another AD GWAS (Ellinghaus et al. 2013).

Validation of gene prioritization – network analysis

The STRING analysis revealed an extensive network that included 25 prioritized genes, centred on key immune regulators, such as STAT3, STAT6, SOCS3, IRF1, TRAF6. It included direct binding interactions between targets prioritized in the current AD GWAS and outside of it – between INPP5D and FCER1G as well as FCER1A, IL7R/STAT3 and TSLP, IL7R and TSLPR, IFNG and IFNGR1; and SOCS3 and IFNGR1. However, when it came to the genes directly taking part in establishing skin barrier, 2nd ranked gene at the epidermal differentiation locus - RPTN, was the only one shown to interact with the late cornified envelope genes.

SUPPLEMENTARY MATERIALS AND METHODS

Bayesian fine-mapping

As input data we used the association statistics from the AD GWAS in individuals of European ancestry, published in Paternoster et al. (2015). For LD structure, in Finemap and Paintor analysis, we used *r* correlations calculated from the 1000 Genomes (Auton et al. 2015) European reference (*n*=503), as it allowed inclusion of more high confidence SNP calls compared to UK Biobank (Bycroft et al. 2018) - 9,265,840 versus 8,391,826. UK Biobank panel (Bycroft et al. 2018) filtered for European ancestry (*n*=48,167) with standard QC applied (Mitchell et al. 2017) was used as LD reference panel in JAM analysis. This was because JAM requires a high number of individuals in the reference and removal of highly correlated SNPs for the genotype matrix to be invertible. To that end, we also pruned SNPs prior to feeding them to JAM. We used a threshold of $r^2 > 0.95$ in Priority Pruner (http://prioritypruner.sourceforge.net/) and set minor allele frequency (MAF) threshold at 0.05 and minimum SNP call rate of 0.9, while force selecting all the index SNPs.

Our fine-mapping integration protocol involved running all the 3 programs with all the SNPs within the interval of 10 kbp, 100 kbp, 500 kbp, 1 Mbp, 3 Mbp centred on the index SNP. We also used r^2 -based and D'-based haploblock intervals defined with the BigLD (Kim et al. 2018) and Gpart (Ah Kim et al. 2019) algorithms, respectively, in 1000 Genome EUR panel.

We ran our Finemap analysis using shotgun stochastic search, whereas for Paintor, we varied the algorithm from exact exhaustive search to MCMC when considering from up to 2 or max. 5 causal SNPs in the region, accordingly. Maximum number of algorithm iterations was set at 1000 in Paintor and 10 million in JAM. In order to permit analysis of a binary trait in JAM, linear mapping of log-odds ratios was performed (Dadaev et al. 2018) and the residual variance inverse gamma hyperpriors were set to "GaussianResidualVarianceInvGammaPrior_a" = 2, "GaussianResidualVarianceInvGammaPrior_b" = proportion of cases * (1-proportion of cases).

When comparing output of Finemap, Paintor and JAM we only considered top fine-mapped SNPs with Bayes Factor > 100 and posterior probability of being causal of at least 0.1.

Variant filtering

We confirmed that we captured all the SNPs within the broadly defined haploblock by re-defining the boundaries based on maximum 3 Mbp interval. Definition of our haploblock changed only in the case of 3 index SNPs: however in all cases there was a stretch of at least 100 kbp (rs61813875 with a very sharp LD decay and rs41293864 situated in the MHC region with complex LD), up to 350 kbp (rs77714197) of SNPs with no $r^2 > 0.2$ so we dismissed those as outliers and used the 1 Mbp interval-defined region in all cases.

Identification of key tissues and cell types

We ran SNPSea using recommended settings and used index SNPs as input. SNPsea considers genes in LD with the index SNPs and for each locus and cell-type combination, selects one gene which shows highest tissue-specificity of expression. The merged gene set across loci is then scored for its cell-type specificity and the resulting score compared to the ones obtained from the null distribution of results for random SNP sets matched on the number of genes in LD in order to obtain a permutation *p*-value.

Briefly, MAGMA gene-based analysis was done using SNPs in the locus interval to identify all the genes associated with our GWAS hits, and subsequently MAGMA gene-property analysis was applied to test tissue type specificity in expression of identified genes.

TwinsUK eQTL identification

We used genotype array data and RPKM -normalized expression in lymphoblastoid cell line (LCL) and skin tissue for females in the TwinsUK cohort (Buil et al. 2015). RPKM values were rank-transformed to normality using GenABEL (Aulchenko et al. 2007) R package before eQTL mapping. *cis*-eQTLs 1.5 Mbp upstream and downstream of TSS were identified using linear mixed model implemented in GEMMA (Zhou and Stephens 2012). We used age as covariate in the analysis involving all samples and centred relatedness matrix as random effects. PEER analysis was run to identify any additional hidden covariates not captured above (Stegle et al. 2012). eQTL associations were identified using the Wald test.

CEDAR eQTL re-identification

In the analysis involving the CEDAR cohort (e.g. Momozawa et al. 2018), we used the publicly available data: imputed genotypes and normalized gene expression values from blood and intestinal cell types ($CD4^+$ T lymphocytes, $CD8^+$ T lymphocytes, $CD19^+$ B lymphocytes, $CD14^+$ monocytes, $CD15^+$ granulocytes, platelets, ileum, colon, rectum) adjusted for 4 top PCs and covariates (sex, age, smoking status, batch). We used GEMMA's linear mixed model and Wald test to re-identify *cis*-eQTLs within 1.5 Mbp upstream and downstream of TSS.

Colocalization with coloc and TWAS

In addition, three-way colocalization of GWAS and whole blood molecular phenotypes: pQTLs and eQTLs was investigated with moloc (Giambartolomei et al. 2018) with default priors but brought no significant results.

In TWAS analysis, we used 100 permutations to conservatively calibrate the imputed gene expression association statistic conditional on the GWAS strength of association and used provided European1000 Genomes panel for LD reference. Any significant gene expression associations with AD were then post-processed to identify conditionally independent associations. In addition, coloc analysis was carried out, based on marginal TWAS weights with provided scripts.

In TWAS analysis, eQTL datasets from the eQTLGen consortium (Võsa et al. 2018), BLUEPRINT (Chen et al. 2016) across 3 cell types (monocytes, neutrophils and T cells) and GTEx ver. 7 (GTEx Consortium 2017) across 48 tissue types were downloaded and. The SMR method applies the Wald Ratio method systematically for all genes with an eQTL at $P<1x10^{-4}$, using lead eQTL as instrumental variables and eczema estimates as the outcome. The HEIDI (heterogeneity in dependent instruments) test was applied to filter out genetically predicted effects which may be attributed to heterogeneity in a region which may lead to spurious results.

Variant functional prediction

We also looked at variant overlap within different regulatory regions: insulator (Wang et al. 2015b), promoter-enhancer interactions (Burren et al. 2017),(Freire-Pritchett et al. 2017),(Gasperini et al. 2019),(Javierre et al. 2016b),(Mifsud et al. 2015),(Mumbach et al. 2017),(Rinaldi et al. 2016),(Rubin et al. 2017),(Wang et al. 2018a), regulatory non-coding RNAs(Chen et al. 2013),(Xie et al. 2014),(De Rie et al. 2017),(Bhattacharya et al. 2014),(Wang et al. 2018b), topologically associating domains (TADs) (Dixon et al. 2012),(ENCODE Project Consortium et al. 2007),(Freire-Pritchett et al. 2017),(Harmston et al. 2017),(Javierre et al. 2016b),(Rao et al. 2014), and CTCF binding sites (Ziebarth et al. 2012) culled from various publications, using giggle (Layer et al. 2018) search engine. Independently, we looked for overlap inside Roadmap regions classified as containing active chromatin state (states 1-8) (Roadmap Epigenomics Consortium et al. 2015) and FAIRE-Seq-determined regions of accessible chromatin in human epidermis during barrier maturation and disruption (Lander et al. 2017). Cell-type specific regulatory elements (Alasoo et al. 2018) were also annotated based on histone marks and chromatin state.

Independent lookups

We have also performed gene and variant lookups among published significant results from various eQTL (Alasoo et al. 2018; Andiappan et al. 2015; Battle et al. 2014; Bonder et al. 2017; Buil et al. 2015; Chen et al. 2016; Ding et al. 2010; Fagny et al. 2017; Fairfax et al. 2014; Fairfax et al. 2012; Gutierrez-Arcelus et al. 2013; Ishigaki et al. 2017; Kasela et al. 2017; Kim et al. 2014; Lappalainen et al. 2013; Lloyd-Jones et al. 2017; Naranbhai et al.

2015; Nedelec et al. 2016; Pageaud et al. 2018; Pala et al. 2017; Quach et al. 2016; Raj et al. 2014; Schmiedel et al. 2018; Thompson et al. 2016; Walsh et al. 2016b; Xia et al. 2011; Yao et al. 2017; Ye et al. 2014; Zhernakova et al. 2017), mQTL (including GoDMC results(Min et al. 2020))(Bonder et al. 2017; Gutierrez-Arcelus et al. 2013) pQTL (Emilsson et al. 2018; Suhre et al. 2017)., hQTL (Chen et al. 2016; Pelikan et al. 2018), caQTL (Maurano et al. 2015), where full GWAS results were not available, as well as differential expression (Cole et al. 2014; Ewald et al. 2015; Ghosh et al. 2015; Sääf et al. 2008; Winge et al. 2011), DNA methylation (Quraishi et al. 2015; Rodríguez et al. 2014) and proteome (Elias et al. 2017; Molin et al. 2015) comparisons between skin in AD patients and healthy controls (Dataset S2). We also interrogated the GWAS Catalog (Milano et al. 2016) (accessed on 11/01/2019) for any variants that have been identified as genome-wide significant in previous GWAS studies on related inflammatory conditions. We used the significance threshold defined in each paper, as it varied due to a number of comparisons made.

Generation of candidate gene and SNP rankings

Standardized results table

Results from individual analyses and lookups were summarized into tables (available in Dataset S1) with the following columns, with required values marked by *:

table id* Particular analysis/ experiment in the study

study id* Unique study which data was sourced from. See Supp Table 3 for references

index SNP* Index SNP representing a genetic locus harbouring AD GWAS hit

current SNP rsid ID of a SNP within the GWAS genetic locus

gene name Gene associated with current SNP or prioritized by other non SNP-based methods, such as DGE (differential gene expression).

FDR/p-value/posterior probability/score/beta A value indicating confidence in true association and/or magnitude of the effect. Note that results used in the analysis were preselected on being significant in the original analysis in the first place, with the exception of scores, which provided a continuous measure of variant deleteriousness.

significance threshold Value threshold for the result to be included in contributing to evidence score

effect allele Effect allele at a given SNP, when available

tissue Target tissue investigated in a given experiment

sample size Sample size of a given experiment

study type* Type of experiment (e.g. ChiP-Seq) or analysis (e.g. eQTL)

cis/trans Interaction in cis- or trans- (for QTLs)

evidence weight* Subjective prior belief in evidence strength, from 1 (highest) to 3 (lowest) – see main Methods

number of SNP significant values* Total number of unique variants in a given locus found among significant hits in the given analysis/experiment

number of gene significant values* Total number of unique genes in a given locus found among significant hits in the given analysis/experiment

n experiments* Number of analyses/ experiments in the study

Calculation of basic score (per gene or variant in a given experiment/analysis):

p is measure of result strength (in the order of preference: False Discovery Rate (FDR), p-value, posterior probability (PP), score, beta; p_{max} is the top lowest (for FDR or p-value) or top absolute highest (PP, score, beta) value present in the dataset

$b = 1 + \frac{\sqrt{-log_{10}(p)}}{\sqrt{-log_{10}(p_{max})}}$ when p refers to FDR or p-value

$b = 1 + \frac{p}{p_{max}}$ when p refers to PP, score or beta value

Calculation of total score (per gene or variant across all experiments/analyses):

gene ranking:

$$\sum_{all\ b} \frac{b * v}{\sqrt{n} * g * \sqrt{s}} * \sqrt{\frac{(t + i)}{2}}$$

variant ranking:

$$\sum_{all\ b} \frac{b * v}{\sqrt{n} * \sqrt{s}} * \sqrt{\frac{(t + i)}{2}}$$

v – evidence weight adjustment: *v* = 1 for evidence weight of 3 (lowest credibility), *v* = 2 for evidence weight of 2, *v* = 20 for evidence weight of 1 (highest credibility)

n – number of experiments in a given study

g – number of genes in a given interval in a given experiment/analysis

s – number of SNPs in a given interval in a given experiment/analysis

t – number of unique study types showing evidence for a given variant/gene

i – number of unique study IDs showing evidence for a given variant/gene

SUPPLEMENTARY FIGURE AND TABLE LEGENDS

Figure S1. Outline of pipeline used to prioritize candidate genes and variants based on AD GWAS.

1) *Left hand-side:* We considered all the genes positioned within a 3Mbp interval centred on index GWAS SNP. *Right hand-side:* We considered variants within the interval around top GWAS SNP defined as follows: consider all the SNPs within 1 Mbp interval around the index SNP and find the furthest SNP (blue circle) in either direction with r^2 >= 0.2 in 1000 Genomes EUR population – these variants define the boundaries of the locus interval (purple shading) within which all SNPs are considered. For locus interval length, we found it ranged from 28,133 bp to 915,373 bp, with median at 228,670 bp. The number of candidate SNPs contained within a locus interval varied from 93 to 10,710, with a median of 758 SNPs.

2) We assembled different types of datasets showing significant results for genes within the loci (*left panel*), both genes and SNPs (*middle panel*) and just SNPs (*right panel*). Tiles represent number of datasets in each category and are further coloured according to subjective evidence strength: red (highest): statistical tests based on full summary statistics, gray (middle): lookups among significant results in experimental studies, blue (low): predictive machine learning models.

3) We summarized the output of for each experiment/analysis in a set of standardized summary tables.

4) We calculated a final score which allowed ranking of all the considered genes and SNPs for a given locus, and prioritization of targets for downstream research.

Figure S2. Overview of contribution of different type of -omics evidence to gene and SNP ranking in all the AD GWAS loci. Each bar represents a number of individual studies in a given category contributing to the sum total of evidence.

Figure S3. Distribution of scores obtained in our model for candidate gene ranking at each locus (labelled with lead SNP).

Figure S4. Distribution of scores obtained in our model for causal SNP ranking at each locus (labelled with lead SNP).

Figure S5. Score by types of evidence in all the AD GWAS loci. Scores for top 3 ranked genes at each locus are shown partitioned by category of evidence – here including the top 10 categories contributing the highest proportion of total score at the top 10 ranked genes for all loci.

Figure S6. Highest-confidence interactions between locus top 3 prioritized genes and other AD genes. AD genes prioritized outside of our pipeline are depicted as olive rectangles. For GWAS AD gene legend, refer to Figure 1.

Table S2. Comparison of Enrichr-based significant enrichment testing results. Top 3 genes combined from across all the AD GWAS loci tested (Genes.top_3) versus other known AD genes (Genes.previously_known), against select ontologies.

Table S3. Colocalization results from coloc and TWAS methods (either base TWAS or TWAS-based coloc) on AD GWAS and eQTLs in tissues from a number of datasets. We report coloc results for genes with posterior probability of a shared causal eQTL and GWAS variant PPH4 > 0. 5 for any gene with at least one strong colocalization result of PPH4 > 0.9, while for TWAS we show genes with genome-wide significant and independent colocalization evidence in conditional and joint analysis. Gene rank at the given locus in our final GWAS gene prioritization model is also given.

Table S4. Results meeting the genome-wide significance threshold with no significant heterogeneity in HEIDI analysis for SMR test of GTEx, Blueprint and eQTLgen eQTL instruments against AD GWAS. Gene rank at the given locus in our final GWAS gene prioritization model is also given.

Fig. S1

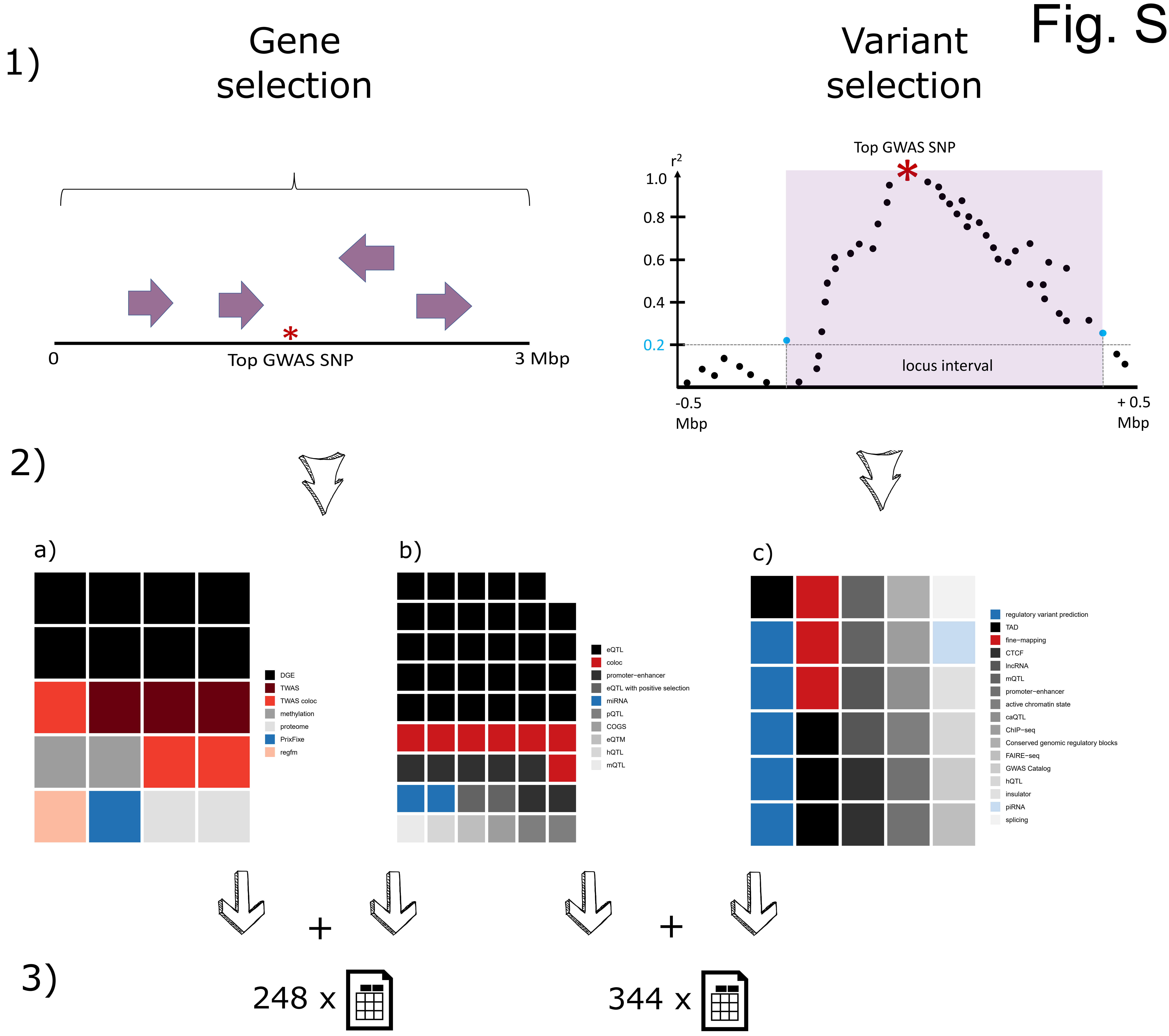

study id	table id	*n* experiments	locus	current SNP	gene name	FDR/p-value/posterior probability/score	study type	evidence weight	total SNP hits	total gene hits
Unique study which data was sourced from	Particular analysis/ experiment in the study	Number of analyses/ experiments in the study dataset	A genetic locus harbouring AD GWAS hit	rsid ID of a SNP within the GWAS genetic locus	Gene associated with current SNP or prioritized by other methods, such as DGE	A value indicating strength of evidence and/or magnitude of the effect	Type of experiment (e.g. ChIP-Seq) or analysis (e.g. eQTL)	Subjective prior belief in analysis strength, from 1 (highest) to 3 (lowest)	Total number of variants in a given locus found among significant hits in the given analysis/ experiment	Total number of genes in a given locus found among significant hits in the given analysis/experiment

4)

For each possible locus and gene/ variant combination, calculate final score:
~ proportional to result significance and effect
~ adjusted downwards based on n experiments, total SNP hits, total gene hits
~ adjusted upwards based on evidence weight and average number of studies & study types

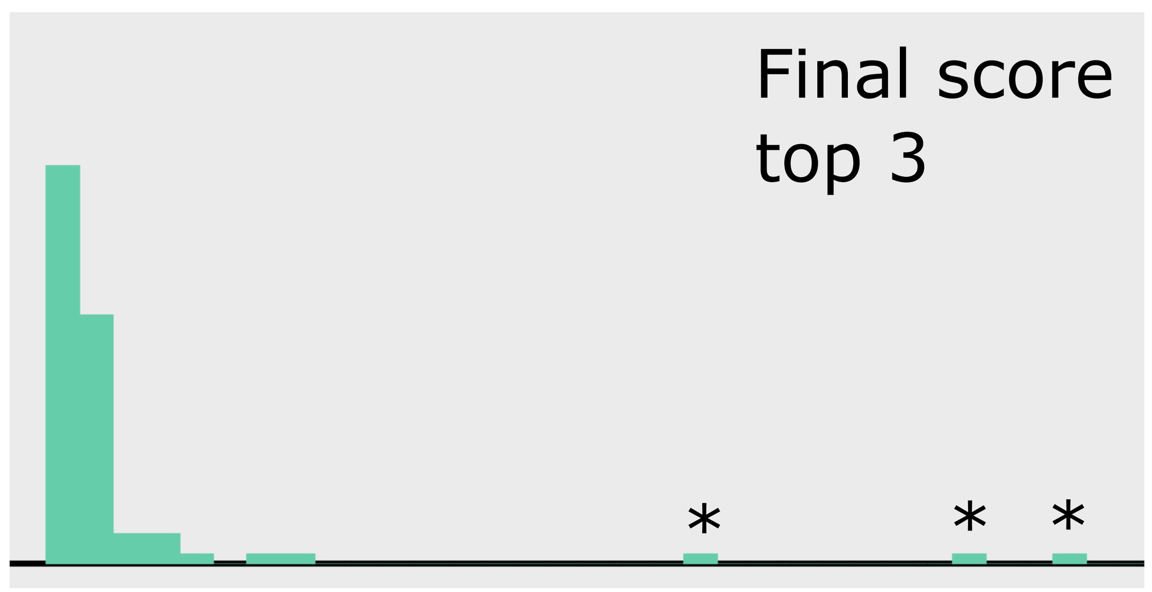

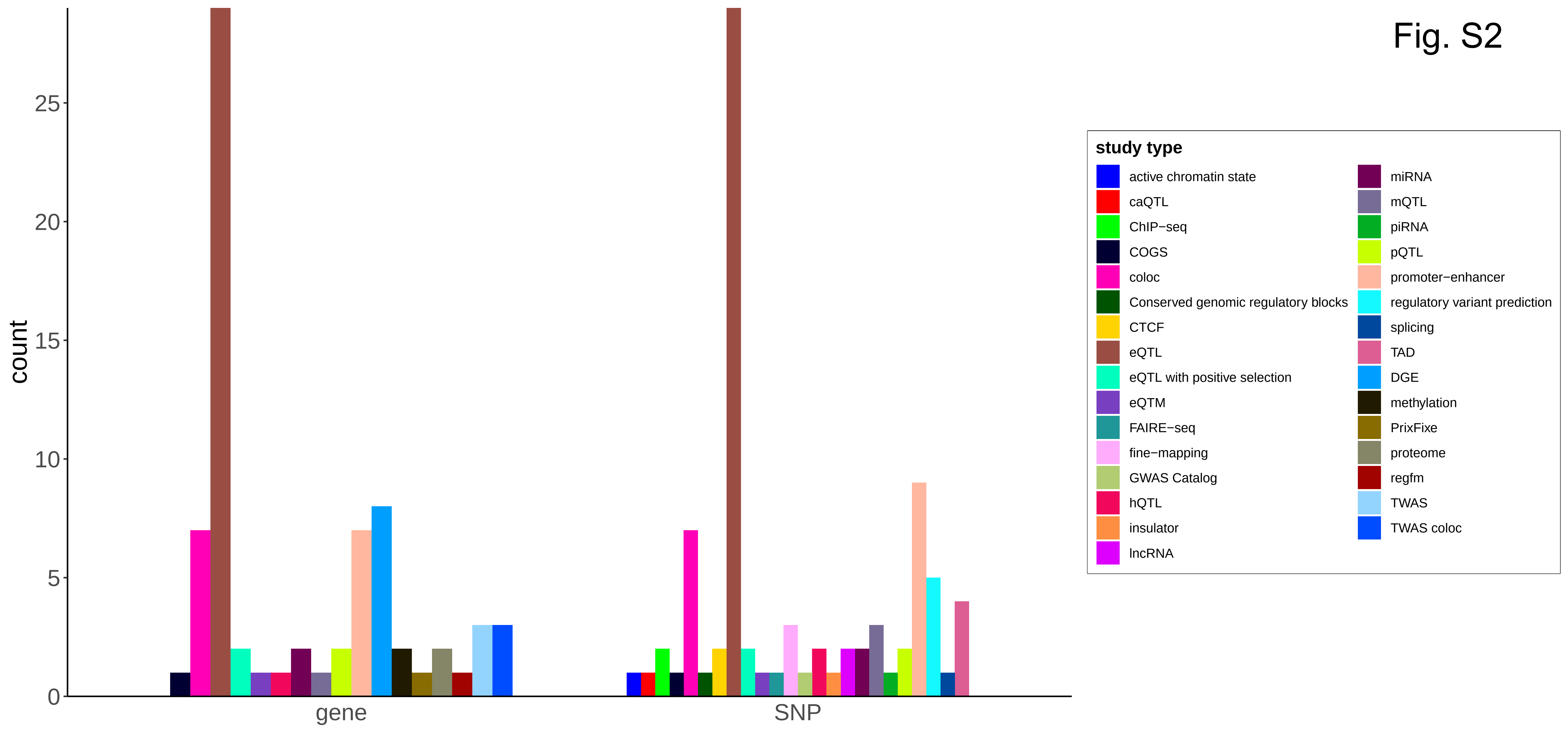

Fig. S2
count
0
5
10
15
20
25
gene
SNP
study type
active chromatin state
caQTL
ChIP−seq
COGS
coloc
Conserved genomic regulatory blocks
CTCF
eQTL
eQTL with positive selection
eQTM
FAIRE−seq
fine−mapping
GWAS Catalog
hQTL
insulator
lncRNA
miRNA
mQTL
piRNA
pQTL
promoter−enhancer
regulatory variant prediction
splicing
TAD
DGE
methylation
PrixFixe
proteome
regfm
TWAS
TWAS coloc

Fig. S3

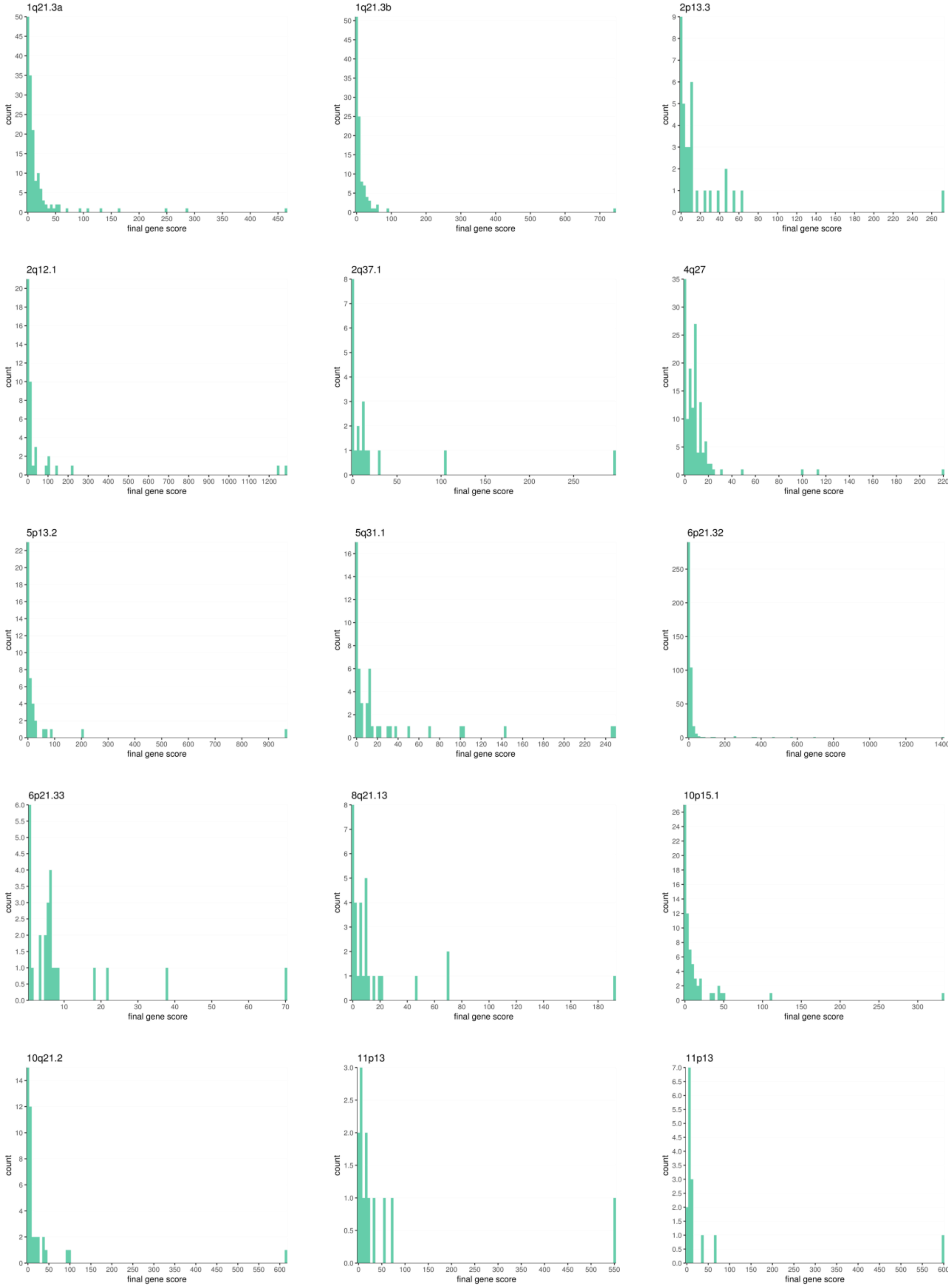

1q21.3a
1q21.3b
2p13.3
2q12.1
2q37.1
4q27
5p13.2
5q31.1
6p21.32
6p21.33
8q21.13
10p15.1
10q21.2
11p13
11p13
count
final gene score

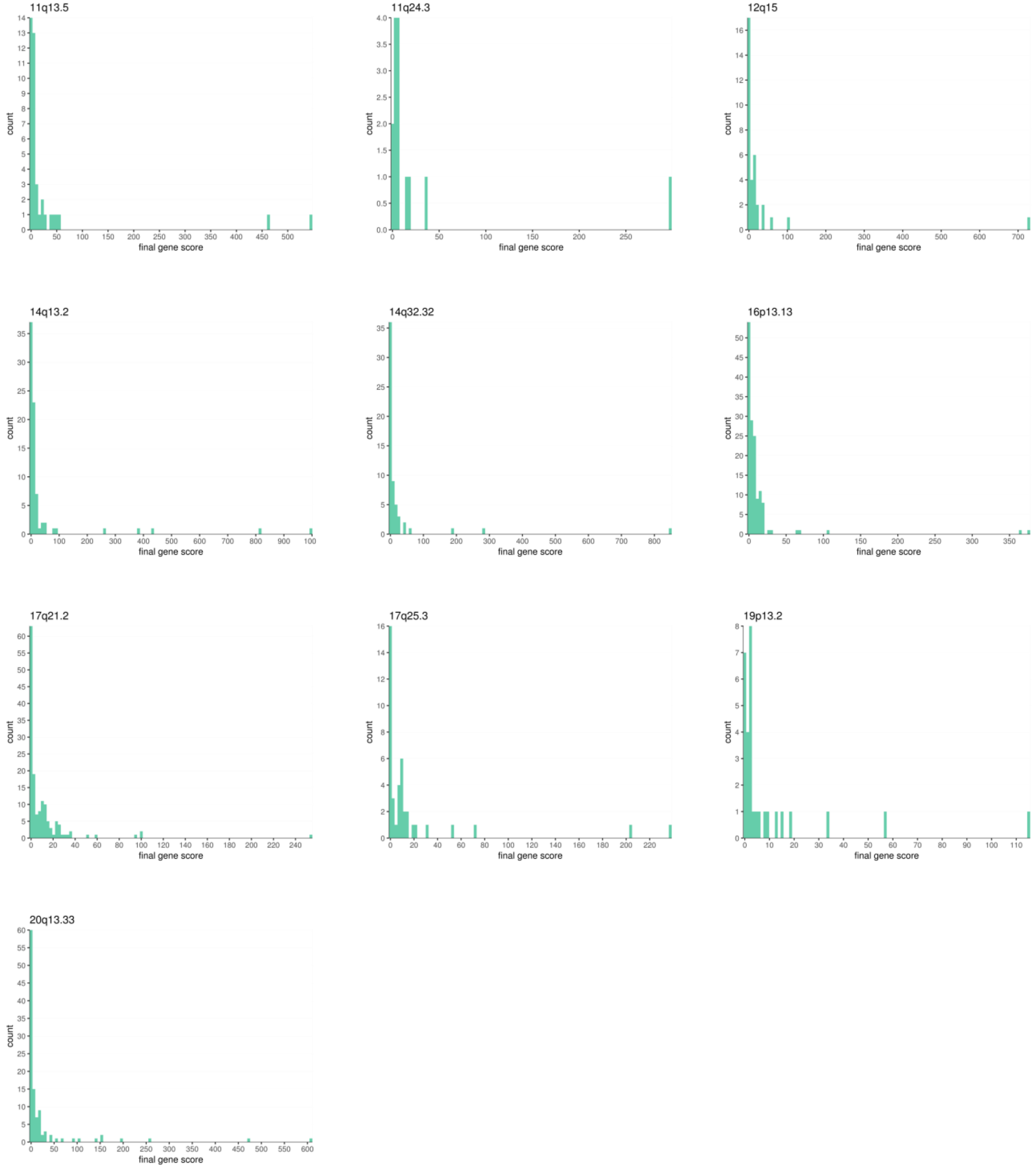
11q13.5
11q24.3
12q15
14q13.2
14q32.32
16p13.13
17q21.2
17q25.3
19p13.2
20q13.33
count
final gene score

Fig. S4

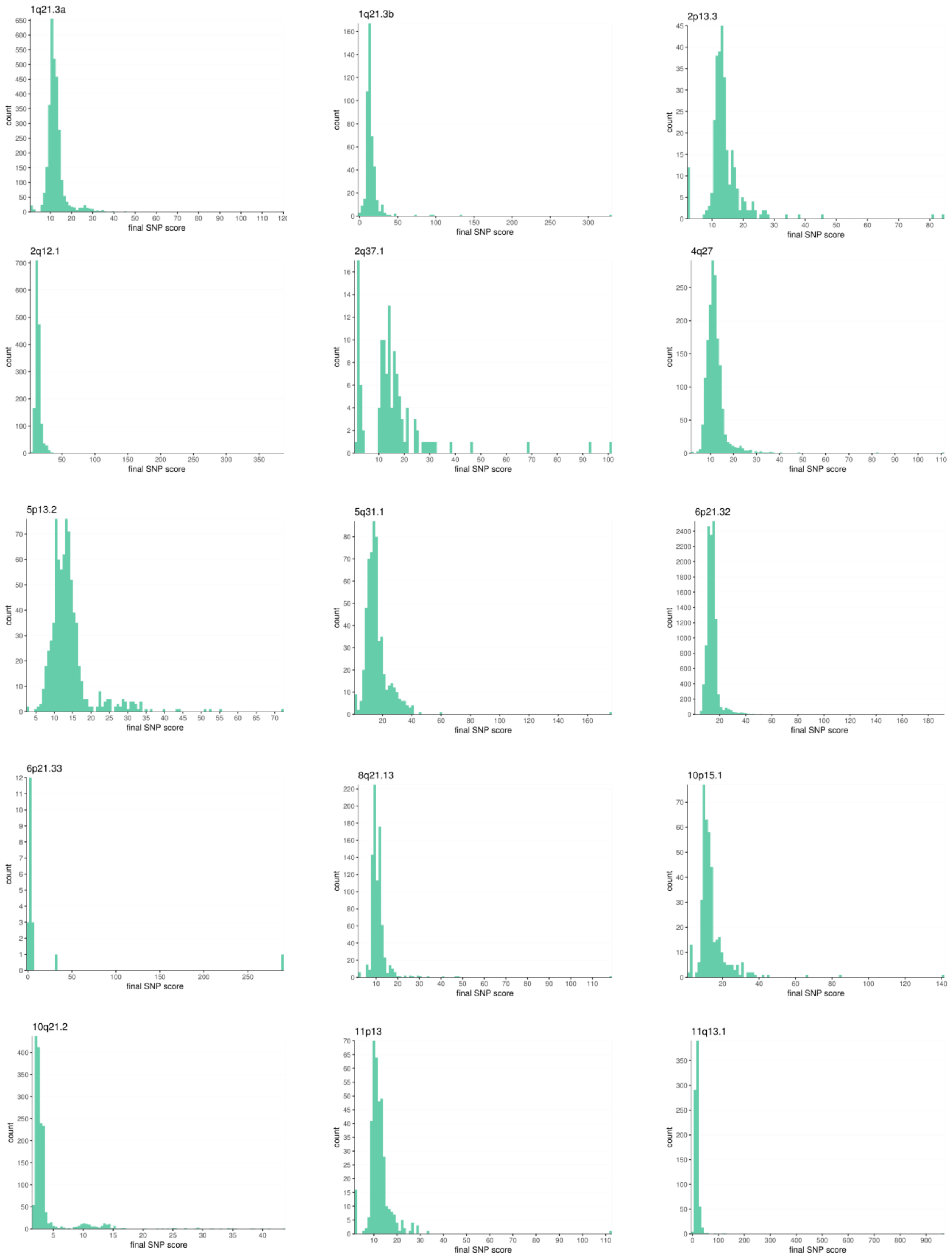

1q21.3a
1q21.3b
2p13.3
2q12.1
2q37.1
4q27
5p13.2
5q31.1
6p21.32
6p21.33
8q21.13
10p15.1
10q21.2
11p13
11q13.1
count
final SNP score

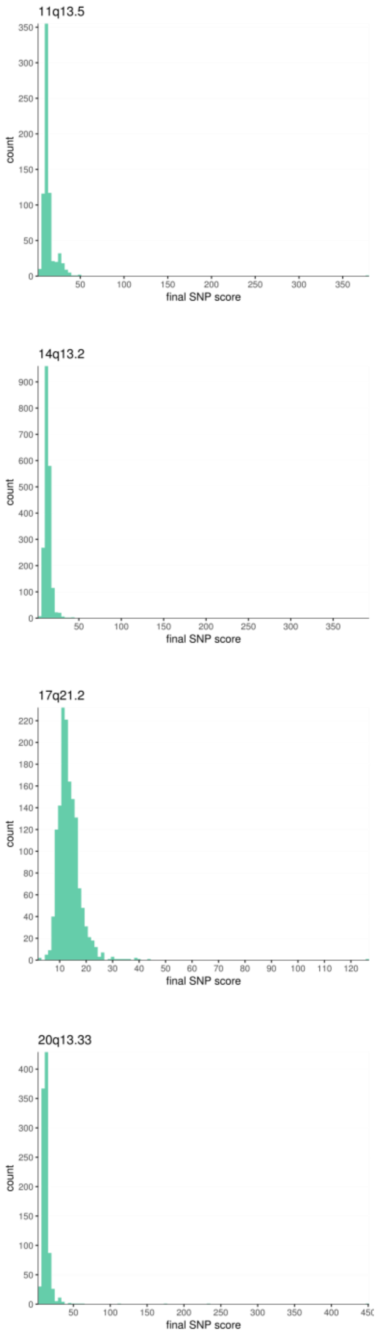
11q13.5
11q24.3
12q15
14q13.2
14q32.32
16p13.13
17q21.2
17q25.3
19p13.2
20q13.33
count
final SNP score

Fig. S5

	coloc	TWAS	Hi−C	eQTL	DGE	regfm	PrixFixe	mQTL	pQTL	hQTL
1q21.3a / rs61813875 HRNR			334	79				4		
1q21.3a / rs61813875 RPTN			279	0	4			2		
1q21.3a / rs61813875 CRNN		38	206	0	4			0		
1q21.3b / rs12730935 IL6R	320	70	83	109	19			6	52	
1q21.3b / rs12730935 UBE2Q1			22	46	8			6		
1q21.3b / rs12730935 ADAR			7	39	9			5		
2p13.3 / rs112111458 CD207	115	76	28	21				5		
2p13.3 / rs112111458 CLEC4F				60				2		
2p13.3 / rs112111458 VAX2			41		4			9		
2q12.1 / rs6419573 IL18R1	265	77	22	900	14			37	3	
2q12.1 / rs6419573 IL18RAP	215	186	41	685	18			26	4	
2q12.1 / rs6419573 IL1RL1	73		33	39	10			21	28	
2q37.1 / rs1057258 INPP5D	60		16	157	16		5	8		
2q37.1 / rs1057258 ATG16L1			72	1	13		3	14		
2q37.1 / rs1057258 RN7SL32P				25						
4q27 / rs6827756 KIAA1109			43	152	4		4	6		
4q27 / rs6827756 BBS12	78		8	19	3			0		
4q27 / rs6827756 TRPC3	57		21	8	3			7		
5p13.2 / rs10214237 IL7R			299	447	16		6	19	35	
5p13.2 / rs10214237 SPEF2			158	7	12		3	10		
5p13.2 / rs10214237 UGT3A2	19		2	8	9	48		0		
5q31.1a / rs12188917 SLC22A5		57		355	20			4		
5q31.1a / rs12188917 IRF1			46	116	39			11		
5q31.1a / rs12188917 RAD50	38	34	7	20	15					
5q31.1b / rs4705962 KIF3A	61		9	143	9			4		
5q31.1b / rs4705962 SLC22A5	71			28	17	115		3		
5q31.1b / rs4705962 PDLIM4	71		6	55				4		
6p21.32 / rs4713555 HLA−DRA	13		1186	63	15			5		4
6p21.32 / rs4713555 HLA−DQB1			112	422	23			6		10
6p21.32 / rs4713555 HLA−DRB1			65	389	5			5		6
6p21.33 / rs41293864 HSPA1B	114	28	1	8	10			3	0	0
6p21.33 / rs41293864 HCG27			19	107	12			4		5
6p21.33 / rs41293864 CSNK2B	51	65	4	7	20			1	0	0
8q21.13 / rs6473227 ZBTB10	66		10	31	10	58	4	4		
8q21.13 / rs6473227 TPD52			31	11	11			12		
8q21.13 / rs6473227 PAG1			6	3	15	42				
10p15.1 / rs6602364 IL2RA	103		118	60	7		5	18		
10p15.1 / rs6602364 RBM17	23	24	3	29	5		3	16		
10p15.1 / rs6602364 PFKFB3			11	9	8			17		
10q21.2 / rs2944542 ADO			132	406	11			32		
10q21.2 / rs2944542 ZNF365			54	13	9		4	17		
10q21.2 / rs2944542 EGR2			50	6	10			17		
11p13 / rs2592555 PRR5L	351		34	131	10		4	33		
11p13 / rs2592555 TRAF6	40		9	4	7			2		
11p13 / rs2592555 COMMD9			9	1	7		2	11		
11q13.1 / rs10791824 CTSW			6	279	22		6	3		
11q13.1 / rs10791824 OVOL1	115	67	10	15	5		4	3		11
11q13.1 / rs10791824 EFEMP2	9	26	13	76	27			3		2
11q13.5 / rs2212434 LRRC32	102		383	22	4			18		
11q13.5 / rs2212434 EMSY	72		232	94	14			29		
11q13.5 / rs2212434 THAP12			11	14	6			14		
11q24.3 / rs7127307 ETS1	110		24	35	26			78		
11q24.3 / rs7127307 FLI1	35									
11q24.3 / rs7127307 APLP2			5	4	9					
12q15 / rs2227483 MDM1			47	532	9			55		
12q15 / rs2227483 IL22			51	6	7		3	29		
12q15 / rs2227483 IFNG	40		6	0	5		5			
14q13.2 / rs2038255 PPP2R3C	277	104	18	485	28				3	3
14q13.2 / rs2038255 KIAA0391	309	195	27	223	13			10		2
14q13.2 / rs2038255 SRP54			60	307	35			1		2
14q32.32 / rs7146581 TRAF3	136		364	253	10		6	14		
14q32.32 / rs7146581 AMN			224	24	3		3	10		
14q32.32 / rs7146581 CDC42BPB			15	132	9		4	13		
16p13.13 / rs2041733 DEXI		29	55	213	9			15		
16p13.13 / rs2041733 CLEC16A	29		129	89	24		4	31		21
16p13.13 / rs2041733 RMI2			18	42	9			21		10
17q21.2 / rs12951971 DHX58			3	216	11			4		
17q21.2 / rs12951971 STAT3	45		9	27	5			4	5	
17q21.2 / rs12951971 RAB5C			4	62	4			1		
17q25.3 / rs11657987 PGS1			42	69	3			35		
17q25.3 / rs11657987 DNAH17			2	25	11			27		
17q25.3 / rs11657987 SOCS3			11	3	15			19		
19p13.2 / rs2918307 ACTL9			98					16		
19p13.2 / rs2918307 ADAMTS10			1	38	8			3		
19p13.2 / rs2918307 MAP2K7	15			2	13					
20q13.33 / rs4809219 STMN3	171	16		342	13			7		
20q13.33 / rs4809219 LIME1	246	112	11	59	10			5		
20q13.33 / rs4809219 ARFRP1	109	12	1	32	4					

score: 0, 100, 200, 300, 400, 500, 600, 700, 800, 900, 1000, 1100

Fig. S6

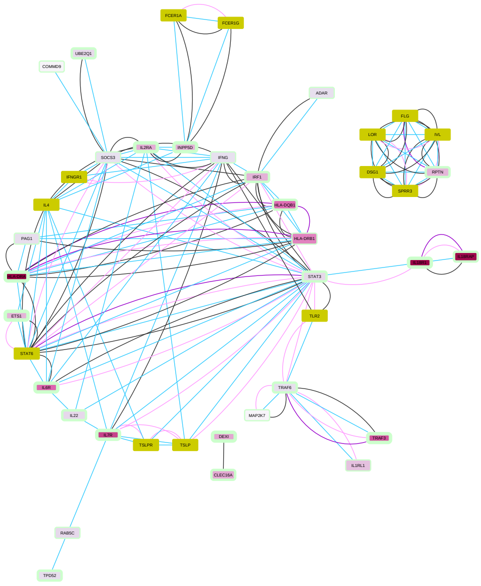
FCER1A
FCER1G
UBE2Q1
COMMD9
ADAR
FLG
LOR
IVL
DSG1
RPTN
SPRR3
IL2RA
INPP5D
SOCS3
IFNG
IRF1
IFNGR1
IL4
HLA-DQB1
PAG1
HLA-DRB1
IL18R1
IL18RAP
HLA-DRA
STAT3
ETS1
TLR2
STAT6
IL6R
TRAF6
IL22
MAP2K7
IL7R
TSLPR
TSLP
DEXI
TRAF3
IL1RL1
CLEC16A
RAB5C
TPD52

REFERENCES

Ah Kim S, Brossard M, Roshandel D, Paterson AD, Bull SB, Yoo YJ. gpart: human genome partitioning and visualization of high-density SNP data by identifying haplotype blocks. Bioinformatics. 2019; Available from: https://doi.org/10.1093/bioinformatics/btz308

Akdis CA, Akdis M, Simon D, Dibbert B, Weber M, Gratzl S, et al. T Cells and T Cell-Derived Cytokines as Pathogenic Factors in the Nonallergic Form of Atopic Dermatitis. J. Invest. Dermatol. 1999;113(4):628–34 Available from: http://www.sciencedirect.com/science/article/pii/S0022202X15406281

Al-mossawi H, Yager N, Taylor C, Lau E, Danielli S, Wit D, et al. Context-specific regulation of monocyte surface IL7R expression and soluble receptor secretion by a common autoimmune risk allele. bioRxiv. 2018; Available from: https://www.biorxiv.org/content/10.1101/262410v4

Al-Shobaili HA, Ahmed AA, Alnomair N. Molecular Genetic of Atopic Dermatitis : An Update. Int. J. Health Sci. (Qassim). 2017;10(1):91–115

Alasoo K, Rodrigues J, Mukhopadhyay S, Knights AJ, Mann AL, Kundu K, et al. Shared genetic effects on chromatin and gene expression indicate a role for enhancer priming in immune response. Nat. Genet. 2018. p. 424–31

Altrichter S, Kriehuber E, Moser J, Valenta R, Kopp T, Stingl G. Serum IgE Autoantibodies Target Keratinocytes in Patients with Atopic Dermatitis. J. Invest. Dermatol. Elsevier Masson SAS; 2008;128(9):2232–9 Available from: http://dx.doi.org/10.1038/jid.2008.80

Amano W, Nakajima S, Kunugi H, Numata Y. Atopic dermatitis and skin disease The Janus kinase inhibitor JTE-052 improves skin barrier function through suppressing signal transducer and activator of transcription 3 signaling. J. Allergy Clin. Immunol. Elsevier Ltd; 136(3):667-677.e7 Available from: http://dx.doi.org/10.1016/j.jaci.2015.03.051

Andiappan AK, Melchiotti R, Poh TY, Nah M, Puan KJ, Vigano E, et al. Genome-wide analysis of the genetic regulation of gene expression in human neutrophils. Nat. Commun. Nature Publishing Group; 2015;6:7971

Asimit JL, Rainbow DB, Fortune MD, Grinberg NF, Wicker LS, Wallace C. Stochastic search and joint fine-mapping increases accuracy and identifies previously unreported associations in immune-mediated diseases. Nat. Commun. Springer US; 2019;10(1) Available from: http://dx.doi.org/10.1038/s41467-019-11271-0

Astle WJ, Elding H, Jiang T, Allen D, Ruklisa D, Mann AL, et al. The allelic landscape of human blood cell trait variation and links to common complex disease. Cell. Elsevier; 2016;167(5):1415–29

Aulchenko YS, Ripke S, Isaacs A, van Duijn CM. GenABEL: an R library for genome-wide association analysis. Bioinformatics. 2007;23(10):1294–6

Auton A, Abecasis GR, Altshuler DM, Durbin RM, Bentley DR, Chakravarti A, et al. A global reference for human genetic variation. Nature. 2015;526(7571):68–74

Battle A, Mostafavi S, Zhu X, Potash JB, Weissman MM, McCormick C, et al. Characterizing the genetic basis of transcriptome diversity through RNA-sequencing of 922 individuals. Genome Res. 2014;24(1):14–24

Beecham AH, Patsopoulos NA, Xifara DK, Davis MF, Kemppinen A, Cotsapas C, et al. Analysis of immune-related loci identifies 48 new susceptibility variants for multiple sclerosis. Nat. Genet. 2013;45(11):1353–62

Bentham J, Morris DL, Cunninghame Graham DS, Pinder CL, Tombleson P, Behrens TW, et al. Genetic association analyses implicate aberrant regulation of innate and adaptive immunity genes in the pathogenesis of systemic lupus erythematosus. Nat. Genet. Nature Publishing Group; 2015;47(12):1457–64 Available

from: http://dx.doi.org/10.1038/ng.3434

Bhattacharya A, Ziebarth JD, Cui Y. PolymiRTS Database 3.0: Linking polymorphisms in microRNAs and their target sites with human diseases and biological pathways. Nucleic Acids Res. 2014;42(D1):86–91

Bishop GA, Stunz LL, Hostager BS. TRAF3 as a multifaceted regulator of B lymphocyte survival and activation. Front. Immunol. 2018;9(SEP):1–10

Bluhm A, Viceconte N, Li F, Rane G, Ritz S, Wang S, et al. ZBTB10 binds the telomeric variant repeat TTGGGG and interacts with TRF2. Nucleic Acids Res. Oxford University Press; 2019;47(4):1896–907

Boguniewicz M, Leung DYM. Atopic dermatitis: a disease of altered skin barrier and immune dysregulation. Immunol. Rev. 2011;242(1):233–46 Available from: https://onlinelibrary.wiley.com/doi/abs/10.1111/j.1600-065X.2011.01027.x

Bonder MJ, Luijk R, Zhernakova D V., Moed M, Deelen P, Vermaat M, et al. Disease variants alter transcription factor levels and methylation of their binding sites. Nat. Genet. 2017;49(1):131–8

Boos AC, Hagl B, Schlesinger A, Halm BE, Ballenberger N, Pinarci M, et al. Atopic dermatitis, STAT3- and DOCK8-hyper-IgE syndromes differ in IgE-based sensitization pattern. Allergy Eur. J. Allergy Clin. Immunol. 2014;69(7):943–53

Bowes J, Budu-Aggrey A, Huffmeier U, Uebe S, Steel K, Hebert HL, et al. Dense genotyping of immune-related susceptibility loci reveals new insights into the genetics of psoriatic arthritis. Nat. Commun. Nature Publishing Group; 2015;6:1–10 Available from: http://dx.doi.org/10.1038/ncomms7046

Boyle AP, Hong EL, Hariharan M, Cheng Y, Schaub MA, Kasowski M, et al. Annotation of functional variation in personal genomes using RegulomeDB. Genome Res. Cold Spring Harbor Lab; 2012;22(9):1790–7

Brdičková N, Brdička T, Angelisová P, Horváth O, Špička J, Hilgert I, et al. LIME: a new membrane Raft-associated adaptor protein involved in CD4 and CD8 coreceptor signaling. J. Exp. Med. Rockefeller University Press; 2003;198(10):1453–62

Breuza L, Poux S, Estreicher A, Famiglietti ML, Magrane M, Tognolli M, et al. The UniProtKB guide to the human proteome. Database. Oxford University Press; 2016;2016

Brunner PM, Israel A, Zhang N, Leonard A, Wen H. Early-onset pediatric atopic dermatitis is characterized by TH2/TH17/TH22-centered inflammation and lipid alterations. J. Allergy Clin. Immunol. Elsevier Ltd; 2018;141(6):2094–106 Available from: http://dx.doi.org/10.1016/j.jaci.2018.02.040

Buil A, Brown AA, Lappalainen T, Viñuela A, Davies MN, Zheng H-F, et al. Gene-gene and gene-environment interactions detected by transcriptome sequence analysis in twins. Nat. Genet. 2015;47(1):88–91

Bult CJ, Eppig JT, Kadin JA, Richardson JE, Blake JA, Group MGD. The Mouse Genome Database (MGD): mouse biology and model systems. Nucleic Acids Res. Oxford University Press; 2008;36(suppl_1):D724--D728

Burren OS, Rubio García A, Javierre BM, Rainbow DB, Cairns J, Cooper NJ, et al. Chromosome contacts in activated T cells identify autoimmune disease candidate genes. Genome Biol. Genome Biology; 2017;18(1):1–19

Busse D, De M, Hobiger K, Thurley K, Flossdorf M, Scheffold A. Competing feedback loops shape IL-2 signaling between helper and regulatory T lymphocytes in cellular microenvironments. Proc. Natl. Acad. Sci. 2010;107(7):3058–63

Bycroft C, Freeman C, Petkova D, Band G, Elliott LT, Sharp K, et al. The UK Biobank resource with deep phenotyping and genomic data. Nature. Springer US; 2018;562(7726):203–9 Available from:

http://www.nature.com/articles/s41586-018-0579-z

Cabanillas B, Novak N. Atopic dermatitis and filaggrin. Curr. Opin. Immunol. Elsevier Ltd; 2016;42(Figure 1):1–8 Available from: http://dx.doi.org/10.1016/j.coi.2016.05.002

Cain SA, Mularczyk EJ, Singh M, Massam-Wu T, Kielty CM. ADAMTS-10 and -6 differentially regulate cell-cell junctions and focal adhesions. Sci. Rep. Nature Publishing Group; 2016;6(May):1–15 Available from: http://dx.doi.org/10.1038/srep35956

Calautti E, Avalle L, Poli V. Psoriasis: A STAT3-Centric View. Int. J. Mol. Sci. MDPI; 2018;19(1):171 Available from: https://www.ncbi.nlm.nih.gov/pubmed/29316631

Chang B, Mandal MNA, Chavali VRM, Hawes NL, Khan NW, Hurd RE, et al. Age-related retinal degeneration (arrd2) in a novel mouse model due to a nonsense mutation in the Mdm1 gene. Hum. Mol. Genet. 2008;17(24):3929–41

Charbaut E, Curmi PA, Ozon S, Lachkar S, Redeker V, Sobel A. Stathmin Family Proteins Display Specific Molecular and Tubulin Binding Properties. J. Biol. Chem. 2001;276(19):16146–54

Chen L, Ge B, Casale FP, Vasquez L, Kwan T, Garrido-Martín D, et al. Genetic Drivers of Epigenetic and Transcriptional Variation in Human Immune Cells. Cell. 2016;167(5):1398-1414.e24

Chen PI, Schauer K, Kong C, Harding AR, Goud B, Stahl PD. Rab5 isoforms orchestrate a “division of labor” in the endocytic network; Rab5c modulates rac-mediated cell motility. PLoS One. 2014;9(2):1–9

Chen G, Wang Z, Wang D, Qiu C, Liu M, Chen X, et al. LncRNADisease: A database for long-non-coding RNA-associated diseases. Nucleic Acids Res. 2013;41(D1):983–6

Chen CC, Yang YH, Lin YT, Hsieh SL, Chiang B-L. Soluble decoy receptor 3: increased levels in atopic patients. J. Allergy Clin. Immunol. Elsevier Limited; 2004;114(1):195

Chiang C-WC-W, Liu W-K, Chiang C-WC-W, Chou C-K. Phosphorylation-dependent association of the G4-1/G5PR regulatory subunit with IKKβ negatively modulates NF-κB activation through recruitment of protein phosphatase 5. Biochem. J. 2010;433(1):187–96

Cole C, Kroboth K, Schurch NJ, Sandilands A, Sherstnev A, O’Regan GM, et al. Filaggrin-stratified transcriptomic analysis of pediatric skin identifies mechanistic pathways in patients with atopic dermatitis. J. Allergy Clin. Immunol. Elsevier Inc.; 2014;134(1):82–91 Available from: http://dx.doi.org/10.1016/j.jaci.2014.04.021

Console L, Scalise M, Tonazzi A, Giangregorio N, Indiveri C. Characterization of Exosomal SLC22A5 (OCTN2) carnitine transporter. Sci. Rep. Springer US; 2018;8(1):2–10 Available from: http://dx.doi.org/10.1038/s41598-018-22170-7

Dadaev T, Saunders EJ, Newcombe PJ, Anokian E, Leongamornlert DA, Brook MN, et al. Fine-mapping of prostate cancer susceptibility loci in a large meta-analysis identifies candidate causal variants. Nat. Commun. 2018;9(1):2256

Dagoneau N, Benoist-Lasselin C, Huber C, Faivre L, Mégarbané A, Alswaid A, et al. ADAMTS10 Mutations in Autosomal Recessive Weill-Marchesani Syndrome. Am. J. Hum. Genet. 2004;75(5):801–6 Available from: http://www.sciencedirect.com/science/article/pii/S0002929707627037

Davison LJ, Wallace C, Cooper JD, Cope NF, Wilson NK, Smyth DJ, et al. Long-range DNA looping and gene expression analyses identify DEXI as an autoimmune disease candidate gene. Hum. Mol. Genet. 2012a;21(2):322–33

Davison LJ, Wallace C, Cooper JD, Cope NF, Wilson NK, Smyth DJ, et al. Long-range DNA looping and gene expression analyses identify DEXI as an autoimmune disease candidate gene. Hum. Mol. Genet. 2012b;21(2):322–33

Davison LJ, Wallace MD, Preece C, Hughes K, Todd JA, Davies B, et al. Dexi disruption depletes gut microbial metabolites and accelerates autoimmune diabetes. bioRxiv. 2018;393421

Ding J, Gudjonsson JE, Liang L, Stuart PE, Li Y, Chen W, et al. Gene expression in skin and lymphoblastoid cells: Refined statistical method reveals extensive overlap in cis-eQTL signals. Am. J. Hum. Genet. 2010;87(6):779–89

Dixon JR, Selvaraj S, Yue F, Kim A, Li Y, Shen Y, et al. Topological domains in mammalian genomes identified by analysis of chromatin interactions. Nature. Nature Publishing Group; 2012;485(7398):376–80

Doherty TA, Soroosh P, Khorram N, Fukuyama S, Rosenthal P, Cho JY, et al. The tumor necrosis factor family member LIGHT is a target for asthmatic airway remodeling. Nat. Med. Nature Publishing Group; 2011;17(5):596

Driskell RR, Lichtenberger BM, Hoste E, Kretzschmar K, Simons BD, Charalambous M, et al. Distinct fibroblast lineages determine dermal architecture in skin development and repair. Nature. Nature Publishing Group; 2013;504(7479):277–81 Available from: http://dx.doi.org/10.1038/nature12783

Elias MS, Long HA, Newman CF, Wilson PA, West A, McGill PJ, et al. Proteomic analysis of filaggrin deficiency identifies molecular signatures characteristic of atopic eczema. J. Allergy Clin. Immunol. Elsevier Inc.; 2017;140(5):1299–309 Available from: https://doi.org/10.1016/j.jaci.2017.01.039

Elias MS, Wright SC, Remenyi J, Abbott JC, Bray SE, Cole C, et al. EMSY expression affects multiple components of the skin barrier with relevance to atopic dermatitis. J. Allergy Clin. Immunol. Elsevier Inc.; 2019;144(2):470–81 Available from: https://doi.org/10.1016/j.jaci.2019.05.024

Ellinghaus D, Baurecht H, Esparza-Gordillo J, Rodríguez E, Matanovic A, Marenholz I, et al. High-density genotyping study identifies four new susceptibility loci for atopic dermatitis. Nat. Genet. 2013;45(7):808–12

Ellinghaus D, Jostins L, Spain SL, Cortes A, Bethune J, Han B, et al. Analysis of five chronic inflammatory diseases identifies 27 new associations and highlights disease-specific patterns at shared loci. Nat. Genet. Nature Publishing Group; 2016;48(5):510

Emilsson V, Ilkov M, Lamb JR, Finkel N, Gudmundsson EF, Pitts R, et al. Co-regulatory networks of human serum proteins link genetics to disease. Science (80-.). 2018;361(6404):769–73 Available from: http://www.sciencemag.org/lookup/doi/10.1126/science.aaq1327

ENCODE Project Consortium T, Birney E, Stamatoyannopoulos JA, Dutta A, Guigó R, Gingeras TR, et al. Identification and analysis of functional elements in 1% of the human genome by the ENCODE pilot project. Nature. Nature Publishing Group; 2007;447(7146):799–816 Available from: https://www.nature.com/articles/nature05874#supplementary-information

Esaki H, Brunner PM, Renert-Yuval Y, Czarnowicki T, Huynh T, Tran G, et al. Early-onset pediatric atopic dermatitis is TH2 but also TH17 polarized in skin. J. Allergy Clin. Immunol. 2016;138(6):1639–51 Available from: http://www.sciencedirect.com/science/article/pii/S0091674916307953

Esparza-Gordillo J, Schaarschmidt H, Liang L, Cookson W, Bauerfeind A, Lee-Kirsch MA, et al. A functional IL-6 receptor (IL6R) variant is a risk factor for persistent atopic dermatitis. J. Allergy Clin. Immunol. 2013;132(2):371–7

Esparza-Gordillo J, Weidinger S, Fölster-Holst R, Bauerfeind A, Ruschendorf F, Patone G, et al. A common variant on chromosome 11q13 is associated with atopic dermatitis. Nat. Genet. 2009;41(5):596–601

Ewald DA, Malajian D, Krueger JG, Workman CT, Wang T, Tian S, et al. Meta-analysis derived atopic dermatitis (MADAD) transcriptome defines a robust AD signature highlighting the involvement of atherosclerosis and lipid metabolism pathways. BMC Med. Genomics. BMC Medical Genomics; 2015;8(60):1–15

Eyerich K, Brown SJ, White EP, Tanaka RJ, Bissonette R, Dhar S, et al. Human and computational models

of atopic dermatitis : A review and perspectives by an expert panel of the International Eczema Council. J. Allergy Clin. Immunol. Elsevier Inc.; 2018;143(1):36–45 Available from: https://doi.org/10.1016/j.jaci.2018.10.033

Fagny M, Paulson JN, Kuijjer ML, Sonawane AR, Chen C-Y, Lopes-Ramos CM, et al. Exploring regulation in tissues with eQTL networks. Proc. Natl. Acad. Sci. 2017;114(37):E7841–50 Available from: http://www.pnas.org/lookup/doi/10.1073/pnas.1707375114

Fairfax BP, Humburg P, Makino S, Naranbhai V, Wong D, Lau E, et al. Innate immune activity conditions the effect of regulatory variants upon monocyte gene expression. Science (80-.). 2014;343(6175)

Fairfax BP, Makino S, Radhakrishnan J, Plant K, Leslie S, Dilthey A, et al. Genetics of gene expression in primary immune cells identifies cell type-specific master regulators and roles of HLA alleles. Nat. Genet. Nature Publishing Group; 2012;44(5):502–10 Available from: http://dx.doi.org/10.1038/ng.2205

Faivre L, Dollfus H, Lyonnet S, Alembik Y, Mégarbané A, Samples J, et al. Clinical homogeneity and genetic heterogeneity in Weill–Marchesani syndrome. Am. J. Med. Genet. Part A. 2003;123A(2):204–7 Available from: https://onlinelibrary.wiley.com/doi/abs/10.1002/ajmg.a.20289

Fernandes S, Srivastava N, Sudan R, Middleton FA, Shergill AK, Ryan JC, et al. SHIP1 deficiency in inflammatory bowel disease is associated with severe Crohn's disease and peripheral T cell reduction. Front. Immunol. 2018;9(MAY):1–10

Ferreira MAR, Matheson MC, Tang CS, Granell R, Ang W, Hui J, et al. Genome-wide association analysis identifies 11 risk variants associated with the asthma with hay fever phenotype. J. Allergy Clin. Immunol. 2014;133(6):1564–71

Ferreira MA, Vonk JM, Baurecht H, Marenholz I, Tian C, Hoffman JD, et al. Shared genetic origin of asthma, hay fever and eczema elucidates allergic disease biology. Nat. Genet. 2017;49(12):1752–7

Fisher SA, Hampe J, Onnie CM, Daly MJ, Curley C, Purcell S, et al. Direct or indirect association in a complex disease: the role of SLC22A4 and SLC22A5 functional variants in Crohn disease. Hum. Mutat. 2006;27(8):778–85 Available from: https://onlinelibrary.wiley.com/doi/abs/10.1002/humu.20358

Fishilevich S, Zimmerman S, Kohn A, Iny Stein T, Olender T, Kolker E, et al. Genic insights from integrated human proteomics in GeneCards. Database. 2016;2016 Available from: https://doi.org/10.1093/database/baw030

Foltz IN, Gerl RE, Wieler JS, Luckach M, Salmon RA, Schrader JW. Human mitogen-activated protein kinase kinase 7 (MKK7) is a highly conserved c-Jun N-terminal kinase/stress-activated protein kinase (JNK/SAPK) activated by environmental stresses and physiological stimuli. J. Biol. Chem. ASBMB; 1998;273(15):9344–51

Fortugno P, Furio L, Teson M, Berretti M, El Hachem M, Zambruno G, et al. The 420K LEKTI variant alters LEKTI proteolytic activation and results in protease deregulation: implications for atopic dermatitis. Hum. Mol. Genet. 2012;21(19):4187–200 Available from: https://doi.org/10.1093/hmg/dds243

Freire-Pritchett P, Schoenfelder S, Várnai C, Wingett SW, Cairns J, Collier AJ, et al. Global reorganisation of cis-regulatory units upon lineage commitment of human embryonic stem cells. Elife. 2017;6:1–26

Fridman JS, Scherle PA, Collins R, Burn T, Neilan CL, Hertel D, et al. Preclinical evaluation of local JAK1 and JAK2 inhibition in cutaneous inflammation. J. Invest. Dermatol. Elsevier; 2011;131(9):1838–44

Furue M, Yamamura K, Kido-Nakahara M, Nakahara T, Fukui Y. Emerging role of interleukin-31 and interleukin-31 receptor in pruritus in atopic dermatitis. Allergy. 2018;73(1):29–36

Galan P, Visscher PM, Liao K, Martin J, Kawaguchi T, De Jager PL, et al. Genetics of rheumatoid arthritis contributes to biology and drug discovery. Nature. Nature Publishing Group; 2013;506(7488):376–81 Available from: http://dx.doi.org/10.1038/nature12873

Galarza-Muñoz G, Briggs FBS, Evsyukova I, Schott-Lerner G, Kennedy EM, Nyanhete T, et al. Human Epistatic Interaction Controls IL7R Splicing and Increases Multiple Sclerosis Risk. Cell. 2017;169(1):72-84.e13

Gan X, Wang J, Wang C, Sommer E, Kozasa T, Srinivasula S, et al. PRR5L degradation promotes mTORC2-mediated PKC-δ phosphorylation and cell migration downstream of Gα 12. Nat. Cell Biol. Nature Publishing Group; 2012;14(7):686–96 Available from: http://dx.doi.org/10.1038/ncb2507

Gao Y, Wang Y, Bouladoux NJ, Belkaid Y, Lazarevic V. Egr2 is required for the activation of the TH17 pathogenetic program. J. Immunol. American Association of Immunologists; 2018;200(1 Supplement):171.9--171.9 Available from: http://www.jimmunol.org/content/200/1_Supplement/171.9

Garbers C, Monhasery N, Aparicio-Siegmund S, Lokau J, Baran P, Nowell MA, et al. The interleukin-6 receptor Asp358Ala single nucleotide polymorphism rs2228145 confers increased proteolytic conversion rates by ADAM proteases. Biochim. Biophys. Acta - Mol. Basis Dis. Elsevier B.V.; 2014;1842(9):1485–94 Available from: http://dx.doi.org/10.1016/j.bbadis.2014.05.018

Garrett-Sinha LA, Kearly A, Satterthwaite AB. The Role of the Transcription Factor Ets1 in Lupus and Other Autoimmune Diseases. Crit. Rev. Immunol. 2016;36(6):485–510 Available from: https://www.ncbi.nlm.nih.gov/pubmed/28845756

Gasperini M, Hill AJ, Trapnell C, Ahituv N, Martin B, Kim S, et al. Genome-wide framework for mapping gene regulation via cellular genetic screens. Cell. 2019;176:1–14

Ghosh D, Ding L, Sivaprasad U, Geh E, Biagini J. Multiple Transcriptome Data Analysis Reveals Biologically Relevant Atopic Dermatitis Signature Genes and Pathways. PLoS One. 2015;10(12):e0144316–e0144316

Giambartolomei C, Zhenli Liu J, Zhang W, Hauberg M, Shi H, Boocock J, et al. A Bayesian framework for multiple trait colocalization from summary association statistics. Bioinformatics. 2018;34(15):2538–45

Gianfelici V, Messina M, Paoloni F, Peragine N, Lauretti A, Fedullo AL, et al. IL7R overexpression in adult acute lymphoblastic leukemia is associated to JAK/STAT pathway mutations and identifies patients who could benefit from targeted therapies. Leuk. Lymphoma. Taylor & Francis; 2018;60(3):829–32 Available from: https://doi.org/10.1080/10428194.2018.1499906

Giridhar PV, Bell SM, Sridharan A, Rajavelu P, Kitzmiller JA, Na C-L, et al. Airway Epithelial KIF3A Regulates Th2 Responses to Aeroallergens. J. Immunol. 2016;197(11):4228–39

Goudy K, Aydin D, Barzaghi F, Gambineri E, Vignoli M, Ciullini S, et al. Human IL2RA null mutation mediates immunodeficiency with lymphoproliferation and autoimmunity. Clin. Immunol. Elsevier Inc.; 2013;146(3):248–61 Available from: http://dx.doi.org/10.1016/j.clim.2013.01.004

Grenningloh R, Kang BY, Ho I-C. Ets-1, a functional cofactor of T-bet, is essential for Th1 inflammatory responses. J. Exp. Med. United States; 2005;201(4):615–26

Gros E, Petzold S, Maintz L, Bieber T, Novak N. Reduced IFN-γ receptor expression and attenuated IFN-γ response by dendritic cells in patients with atopic dermatitis. J. Allergy Clin. Immunol. 2011;128(5):1015–21 Available from: http://www.sciencedirect.com/science/article/pii/S0091674911009213

GTEx Consortium. Genetic effects on gene expression across human tissues. Nature. 2017;550(7675):204–13

Gueneau L, Fish RJ, Shamseldin HE, Voisin N, Tran Mau-Them F, Preiksaitiene E, et al. KIAA1109 Variants Are Associated with a Severe Disorder of Brain Development and Arthrogryposis. Am. J. Hum. Genet. 2018;102(1):116–32 Available from: http://www.sciencedirect.com/science/article/pii/S0002929717304913

Gulko B, Hubisz MJ, Gronau I, Siepel A. A method for calculating probabilities of fitness consequences for

point mutations across the human genome. Nat. Genet. Nature Publishing Group; 2015;47(3):276–83 Available from: http://dx.doi.org/10.1038/ng.3196

Gupta S, Deepti A, Deegan S, Lisbona F, Hetz C, Samali A. HSP72 protects cells from ER stress-induced apoptosis via enhancement of IRE1α-xbp1 signaling through a physical interaction. PLoS Biol. 2010;8(7)

Guryanova OA, Drazba JA, Frolova EI, Chumakov PM. Actin cytoskeleton remodeling by the alternatively spliced isoform of PDLIM4/RIL protein. J. Biol. Chem. ASBMB; 2011;286(30):26849–59

Gutierrez-Arcelus M, Lappalainen T, Montgomery SB, Buil A, Ongen H, Yurovsky A, et al. Passive and active DNA methylation and the interplay with genetic variation in gene regulation. Elife. 2013;2013(2):1–18

Gutierrez-Arcelus M, Ongen H, Lappalainen T, Montgomery SB, Buil A, Yurovsky A, et al. Tissue-Specific Effects of Genetic and Epigenetic Variation on Gene Regulation and Splicing. PLoS Genet. 2015;11(1):1–25

Guttman-Yassky E, Brunner PM, Neumann AU, Khattri S, Pavel AB, Malik K, et al. Efficacy and safety of fezakinumab (an IL-22 monoclonal antibody) in adults with moderate-to-severe atopic dermatitis inadequately controlled by conventional treatments: A randomized, double-blind, phase 2a trial. J. Am. Acad. Dermatol. Elsevier Inc; 2018;78(5):872-881.e6 Available from: https://doi.org/10.1016/j.jaad.2018.01.016

Hackett TL, De Bruin HG, Shaheen F, Van Den Berge M, Van Oosterhout AJ, Postma DS, et al. Caveolin-1 controls airway epithelial barrier function implications for asthma. Am. J. Respir. Cell Mol. Biol. 2013;49(4):662–71

Haritunians T, Jones MR, McGovern DPB, Shih DQ, Barrett RJ, Derkowski C, et al. Variants in ZNF365 isoform D are associated with Crohn’s disease. Gut. BMJ Publishing Group; 2011;60(8):1060–7 Available from: https://gut.bmj.com/content/60/8/1060

Harmston N, Ing-Simmons E, Tan G, Perry M, Merkenschlager M, Lenhard B. Topologically associating domains are ancient features that coincide with Metazoan clusters of extreme noncoding conservation. Nat. Commun. Springer US; 2017;8(1) Available from: http://dx.doi.org/10.1038/s41467-017-00524-5

Hedl M, Zheng S, Abraham C. The IL18RAP Region Disease Polymorphism Decreases IL-18RAP/IL-18R1/IL-1R1 Expression and Signaling through Innate Receptor-Initiated Pathways. J. Immunol. 2014;192(12):5924–32

Hesse D, Radloff K, Jaschke A, Lagerpusch M, Chung B, Tailleux A, et al. Hepatic trans-Golgi action coordinated by the GTPase ARFRP1 is crucial for lipoprotein lipidation and assembly. J. Lipid Res. The American Society for Biochemistry and Molecular Biology; 2014;55(1):41–52 Available from: https://www.ncbi.nlm.nih.gov/pubmed/24186947

Hill MM, Bastiani M, Luetterforst R, Kirkham M, Kirkham A, Nixon SJ, et al. PTRF-Cavin, a Conserved Cytoplasmic Protein Required for Caveola Formation and Function. Cell. 2008;132(1):113–24

Hillmer EJ, Zhang H, Li HS, Watowich SS. STAT3 signaling in immunity. Cytokine Growth Factor Rev. Elsevier Ltd; 2016;31:1–15 Available from: http://dx.doi.org/10.1016/j.cytogfr.2016.05.001

Hirano A, Goto M, Mitsui T, Hashimoto-Hachiya A, Tsuji G, Furue M. Antioxidant Artemisia princeps Extract Enhances the Expression of Filaggrin and Loricrin via the AHR/OVOL1 Pathway. Int. J. Mol. Sci. 2017;18(9) Available from: http://www.mdpi.com/1422-0067/18/9/1948

Hirota T, Takahashi A, Kubo M, Tsunoda T, Tomita K, Sakashita M, et al. Genome-wide association study identifies eight new susceptibility loci for atopic dermatitis in the Japanese population. Nat. Genet. Nature Publishing Group; 2012;44(11):1222–6 Available from: http://dx.doi.org/10.1038/ng.2438

Hoffjan S, Stemmler S. On the role of the epidermal differentiation complex in ichthyosis vulgaris, atopic

dermatitis and psoriasis. Br. J. Dermatol. England; 2007;157(3):441–9

Hommel A, Hesse D, Völker W, Jaschke A, Moser M, Engel T, et al. The ARF-Like GTPase ARFRP1 Is Essential for Lipid Droplet Growth and Is Involved in the Regulation of Lipolysis. Mol. Cell. Biol. American Society for Microbiology Journals; 2010;30(5):1231–42 Available from: https://mcb.asm.org/content/30/5/1231

Hur EM, Son M, Lee O-H, Choi YB, Park C, Lee H, et al. LIME, a Novel Transmembrane Adaptor Protein, Associates with p56 lck and Mediates T Cell Activation. J. Exp. Med. 2003;198(10):1463–73

Inshaw JRJ, Cutler AJ, Burren OS, Stefana MI, Todd JA. Approaches and advances in the genetic causes of autoimmune disease and their implications. Nat. Immunol. Springer US; 2018;19(7):674–84 Available from: http://dx.doi.org/10.1038/s41590-018-0129-8

Ishigaki K, Kochi Y, Suzuki A, Tsuchida Y, Tsuchiya H, Sumitomo S, et al. Polygenic burdens on cell-specific pathways underlie the risk of rheumatoid arthritis. Nat. Genet. Nature Publishing Group; 2017;49(7):1120–5 Available from: http://dx.doi.org/10.1038/ng.3885

Ishigaki K, Kochi Y, Yamamoto K. Genetics of human autoimmunity: From genetic information to functional insights. Clin. Immunol. Elsevier Inc.; 2018;186:9–13 Available from: https://doi.org/10.1016/j.clim.2017.08.017

Javierre BM, Sewitz S, Cairns J, Wingett SW, Várnai C, Thiecke MJ, et al. Lineage-Specific Genome Architecture Links Enhancers and Non-coding Disease Variants to Target Gene Promoters. Cell. 2016a;167(5):1369-1384.e19

Javierre BM, Sewitz S, Cairns J, Wingett SW, Várnai C, Thiecke MJ, et al. Lineage-Specific Genome Architecture Links Enhancers and Non-coding Disease Variants to Target Gene Promoters. Cell. 2016b;167(5):1369-1384.e19

Johansson E, Biagini Myers JM, Martin LJ, He H, Ryan P, LeMasters GK, et al. Identification of two early life eczema and non-eczema phenotypes with high risk for asthma development. Clin. Exp. Allergy. 2019;49(6):829–37

Kabashima-Kubo R, Nakamura M, Sakabe J ichi, Sugita K, Hino R, Mori T, et al. A group of atopic dermatitis without IgE elevation or barrier impairment shows a high Th1 frequency: Possible immunological state of the intrinsic type. J. Dermatol. Sci. Japanese Society for Investigative Dermatology; 2012;67(1):37–43 Available from: http://dx.doi.org/10.1016/j.jdermsci.2012.04.004

Kalamaha K, Reis E, Newton S, Roche C, Julson J, Fernandes H, et al. Atopic dermatitis: a review of evolving targeted therapies. Expert Rev. Clin. Immunol. Taylor & Francis; 2019;15(3):275–88 Available from: https://doi.org/10.1080/1744666X.2019.1560267

Kasela S, Kisand K, Tserel L, Kaleviste E, Remm A, Fischer K, et al. Pathogenic implications for autoimmune mechanisms derived by comparative eQTL analysis of CD4+versus CD8+T cells. PLoS Genet. 2017;13(3)

Kawasaki K, Kuge O, Chang S, Heacock PN, Rho M, Suzuki K, et al. Isolation of a chinese hamster ovary (CHO) cDNA encoding phosphatidylglycerophosphate (PGP) synthase, expression of which corrects the mitochondrial abnormalities of a PGP synthase-defective mutant of CHO-K1 cells. J. Biol. Chem. 1999;274(3):1828–34

Kichaev G, Bhatia G, Loh P, Gazal S, Burch K, Freund MK, et al. Leveraging Polygenic Functional Enrichment to Improve GWAS Power. Am. J. Hum. Genet. ElsevierCompany.; 2019;1–11 Available from: https://doi.org/10.1016/j.ajhg.2018.11.008

Kim-Hellmuth S, Bechheim M, Pütz B, Mohammadi P, Nédélec Y, Giangreco N, et al. Genetic regulatory effects modified by immune activation contribute to autoimmune disease associations. Nat. Commun.

Springer US; 2017;8(1) Available from: http://dx.doi.org/10.1038/s41467-017-00366-1

Kim JH, Bae HC, Ko NY, Lee SH, Jeong SH, Lee H, et al. Thymic stromal lymphopoietin downregulates filaggrin expression by signal transducer and activator of transcription 3 (STAT3) and extracellular signal-regulated kinase (ERK) phosphorylation in keratinocytes. J. Allergy Clin. Immunol. Elsevier; 2015;136(1):205–8

Kim S, Becker J, Bechheim M, Kaiser V, Noursadeghi M, Fricker N, et al. Characterizing the genetic basis of innate immune response in TLR4-activated human monocytes. Nat. Commun. 2014;5(5236):1–7

Kim JH, Cha JY, Cheong HS, Park JS, Jang AS, Uh ST, et al. KIF3A, a cilia structural gene on chromosome 5q31, and its polymorphisms show an association with aspirin hypersensitivity in asthma. J. Clin. Immunol. 2011;31(1):112–21

Kim SA, Cho CS, Kim SR, Bull SB, Yoo YJ. A new haplotype block detection method for dense genome sequencing data based on interval graph modeling of clusters of highly correlated SNPs. Bioinformatics. 2018;34(3):388–97

Kim JE, Kim JS, Cho DH, Park HJ. Molecular mechanisms of cutaneous inflammatory disorder: Atopic dermatitis. Int. J. Mol. Sci. 2016;17(8)

Kinjyo I, Inoue H, Hamano S, Fukuyama S, Yoshimura T, Koga K, et al. Loss of SOCS3 in T helper cells resulted in reduced immune responses and hyperproduction of interleukin 10 and transforming growth factor-beta 1. J. Exp. Med. 2006;203(4):1021–31

Kitabatake M, Toda T, Kuwahara K, Igarashi H, Ohtsuji M, Tsurui H, et al. Transgenic Overexpression of G5PR That Is Normally Augmented in Centrocytes Impairs the Enrichment of High-Affinity Antigen-Specific B Cells, Increases Peritoneal B-1a Cells, and Induces Autoimmunity in Aged Female Mice. J. Immunol. 2012;189(3):1193–201

Klunker S, Trautmann A, Akdis M, Verhagen J, Schmid-Grendelmeier P, Blaser K, et al. A second step of chemotaxis after transendothelial migration: keratinocytes undergoing apoptosis release IFN-gamma-inducible protein 10, monokine induced by IFN-gamma, and IFN-gamma-inducible alpha-chemoattractant for T cell chemotaxis toward epidermis in. J. Immunol. Am Assoc Immnol; 2003;171(2):1078–84

Kottyan LC, D’mello RJ, Miller D, Maddox A, Stucke E, Rochman M, et al. 11q13 Is an Allergic Risk-Locus That Increases Eoe Risk and Increases LRRC32 Expression. J. Allergy Clin. Immunol. Elsevier Science Ltd.; 2016;137(2):AB279

Kowal K, Pampuch A, Sacharzewska E, Kowal-Bielecka O. Serum concentration of TNFSF14/LIGHT receptors DcR3 and HVEM in asthma patients. J. Allergy Clin. Immunol. Elsevier Limited; 2019;143(2):AB8

Kutz WE, Wang LW, Bader HL, Majors AK, Iwata K, Traboulsi EI, et al. ADAMTS10 protein interacts with fibrillin-1 and promotes its deposition in extracellular matrix of cultured fibroblasts. J. Biol. Chem. 2011;286(19):17156–67

Lander JM, Supp DM, He H, Martin LJ, Chen X, Weirauch MT, et al. Analysis of chromatin accessibility in human epidermis identifies putative barrier dysfunction-sensing enhancers. PLoS One. 2017;12(9):1–22 Available from: http://dx.doi.org/10.1371/journal.pone.0184500

Lappalainen T, Sammeth M, Friedländer MR, ’T Hoen PAC, Monlong J, Rivas MA, et al. Transcriptome and genome sequencing uncovers functional variation in humans. Nature. 2013;501(7468):506–11

Layer RM, Pedersen BS, Disera T, Marth GT, Gertz J, Quinlan AR. GIGGLE: a search engine for large-scale integrated genome analysis. Nat. Methods. 2018;15(2):123–6

Lee C-H, Hong C-H, Yu W-T, Chuang H-Y, Huang S-K, Chen G-S, et al. Mechanistic correlations between two itch biomarkers, cytokine interleukin-31 and neuropeptide β-endorphin, via STAT3/calcium axis in

atopic dermatitis. Br. J. Dermatol. John Wiley & Sons, Ltd (10.1111); 2012;167(4):794–803 Available from: https://doi.org/10.1111/j.1365-2133.2012.11047.x

Lee C-G, Kwon H-K, Kang H, Kim Y, Nam JH, Won YH, et al. Ets1 suppresses atopic dermatitis by suppressing pathogenic T cell responses. JCI Insight. 2019;4(5)

Lee H, Ryu WI, Kim HJ, Bae HC, Ryu HJ, Shin JJ, et al. TSLP Down-Regulates S100A7 and ß-Defensin 2 Via the JAK2/STAT3-Dependent Mechanism. J. Invest. Dermatol. The Authors; 2016;136(12):2427–35 Available from: http://dx.doi.org/10.1016/j.jid.2016.07.027

Leung DYM, Gao PS, Grigoryev DN, Rafaels NM, Streib JE, Howell MD, et al. Human atopic dermatitis complicated by eczema herpeticum is associated with abnormalities in IFN-γ response. J. Allergy Clin. Immunol. Elsevier Ltd; 2011;127(4):965-973.e5 Available from: http://dx.doi.org/10.1016/j.jaci.2011.02.010

Li Y, de Haar C, Chen M, Deuring J, Gerrits MM, Smits R, et al. Disease-related expression of the IL6/STAT3/SOCS3 signalling pathway in ulcerative colitis and ulcerative colitis-related carcinogenesis. Gut. BMJ Publishing Group; 2010;59(2):227–35 Available from: https://gut.bmj.com/content/59/2/227

Li J, Jørgensen SF, Maggadottir SM, Bakay M, Warnatz K, Glessner J, et al. Association of CLEC16A with human common variable immunodeficiency disorder and role in murine B cells. Nat. Commun. 2015a;6

Li F, Liu X, Zhou W, Yang X, Shen Y. Auto-inhibitory mechanism of the human mitochondrial RNase P protein complex. Sci. Rep. Nature Publishing Group; 2015b;5:9878

Li L, Zhou D, Zheng Y, Xie W. Expression and functions of the STAT3-SCLIP pathway in chronic myeloid leukemia cells. Exp. Ther. Med. 2016;12(5):3381–6

Liao W, Lin J-X, Leonard WJ. Interleukin-2 at the Crossroads of Effector Responses, Tolerance, and Immunotherapy. Immunity. 2013;38(1):13–25 Available from: http://www.sciencedirect.com/science/article/pii/S1074761313000113

Lin WW, Hsieh SL. Decoy receptor 3: A pleiotropic immunomodulator and biomarker for inflammatory diseases, autoimmune diseases and cancer. Biochem. Pharmacol. Elsevier Inc.; 2011;81(7):838–47 Available from: http://dx.doi.org/10.1016/j.bcp.2011.01.011

Lin C-T, Huang W-N, Hsieh C-W, Chen Y-M, Chen D-Y, Hsieh T-Y, et al. Safety and effectiveness of tocilizumab in treating patients with rheumatoid arthritis – A three-year study in Taiwan. J. Microbiol. Immunol. Infect. 2019;52(1):141–50

Liu L, Brown D, McKee M, LeBrasseur NK, Yang D, Albrecht KH, et al. Deletion of Cavin/PTRF Causes Global Loss of Caveolae, Dyslipidemia, and Glucose Intolerance. Cell Metab. 2008;8(4):310–7

Lloyd-Jones LR, Holloway A, McRae A, Yang J, Small K, Zhao J, et al. The Genetic Architecture of Gene Expression in Peripheral Blood. Am. J. Hum. Genet. ElsevierCompany.; 2017;100(2):228–37 Available from: http://dx.doi.org/10.1016/j.ajhg.2016.12.008

Lorenz U. SHP-1 and SHP-2 in T cells: Two phosphatases functioning at many levels. Immunol. Rev. 2009;228(1):342–59

Van Luijn MM, Kreft KL, Jongsma ML, Mes SW, Wierenga-Wolf AF, Van Meurs M, et al. Multiple sclerosis-associated CLEC16A controls HLA class II expression via late endosome biogenesis. Brain. 2015;138(6):1531–47

Ma C, Lin W, Liu Z, Tang W, Gautam R, Li H, et al. NDR1 protein kinase promotes IL-17- and TNF-α-mediated inflammation by competitively binding TRAF3. EMBO Rep. 2017;18(4):586–602

Ma L, Xue HB, Guan XH, Shu CM, Wang F, Zhang JH, et al. The Imbalance of Th17 cells and CD4(+) CD25(high) Foxp3(+) Treg cells in patients with atopic dermatitis. J. Eur. Acad. Dermatology Venereol.

2014;28(8):1079–86

Mahmudul F, Ronny H, Igarashi H, Sakaguchi N. BCR-crosslinking induces a transcription of protein phosphatase component G5PR that is required for mature B-cell survival. 2006;340:338–46

Manz J, Rodrı E, Elsharawy A, Oesau E, Petersen B, Mayr G, et al. Targeted Resequencing and Functional Testing Identifies Low-Frequency Missense Variants in the Gene Encoding GARP as Significant Contributors to Atopic Dermatitis Risk. 2016;136

Van de Mark D, Kong D, Loncarek J, Stearns T. MDM1 is a microtubule-binding protein that negatively regulates centriole duplication. Mol. Biol. Cell. 2015;26(21):3788–802

Masson N, Keeley TP, Giuntoli B, White MD, Puerta ML, Perata P, et al. Conserved N-terminal cysteine dioxygenases transduce responses to hypoxia in animals and plants. Science (80-.). 2019;365(6448):65 LP – 69 Available from: http://science.sciencemag.org/content/365/6448/65.abstract

Maurano MT, Haugen E, Sandstrom R, Vierstra J, Shafer A, Kaul R, et al. Large-scale identification of sequence variants influencing human transcription factor occupancy in vivo. Nat. Genet. 2015;47(12):1393–401

Meno C, Okuda Y, Hatanaka H, Nakaki R, Ogasawara O, Ohta T, et al. ChIP-Atlas: a data-mining suite powered by full integration of public ChIP-seq data. EMBO Rep. 2018;19(12):e46255

Metelli A, Salem M, Wallace CH, Wu BX, Li A, Li X, et al. Immunoregulatory functions and the therapeutic implications of GARP-TGF-β in inflammation and cancer. J. Hematol. Oncol. Journal of Hematology & Oncology; 2018;11(1):1–11

Miao T, Raymond M, Bhullar P, Ghaffari E, Symonds ALJ, Meier UC, et al. Early Growth Response Gene-2 Controls IL-17 Expression and Th17 Differentiation by Negatively Regulating Batf. J. Immunol. American Association of Immunologists; 2013;190(1):58–65 Available from: http://www.jimmunol.org/content/190/1/58

Miao T, Symonds ALJ, Singh R, Symonds JD, Ogbe A, Omodho B, et al. Egr2 and 3 control adaptive immune responses by temporally uncoupling expansion from T cell differentiation. J. Exp. Med. 2017;214(6):1787–808

Mifsud B, Tavares-Cadete F, Young AN, Sugar R, Schoenfelder S, Ferreira L, et al. Mapping long-range promoter contacts in human cells with high-resolution capture Hi-C. Nat. Genet. Nature Publishing Group; 2015;47(6):598–606 Available from: http://dx.doi.org/10.1038/ng.3286

Milano A, McMahon A, Welter D, Bowler E, Hastings E, Cunningham F, et al. The new NHGRI-EBI Catalog of published genome-wide association studies (GWAS Catalog). Nucleic Acids Res. 2016;45(D1):D896–901 Available from: https://dx.doi.org/10.1093/nar/gkw1133

Milner JD, Brenchley JM, Laurence A, Freeman AF, Hill BJ, Elias KM, et al. Impaired TH17 cell differentiation in subjects with autosomal dominant hyper-IgE syndrome. Nature. 2008;452(7188):773–6

Min JL, Hemani G, Hannon E, Dekkers KF, Castillo-Fernandez J, Luijk R, et al. Genomic and phenomic insights from an atlas of genetic effects on DNA methylation. medRxiv. Cold Spring Harbor Laboratory Press; 2020; Available from: https://www.medrxiv.org/content/early/2020/09/03/2020.09.01.20180406

Min S, Li L, Zhang M, Zhang Y, Liang X, Xie Y, et al. TGF-β-associated miR-27a inhibits dendritic cell-mediated differentiation of Th1 and Th17 cells by TAB3, p38 MAPK, MAP2K4 and MAP2K7. Genes Immun. Macmillan Publishers Limited; 2012;13(8):621 Available from: https://doi.org/10.1038/gene.2012.45

Mitchell R, Hemani G, Dudding T, Paternoster L. UK Biobank Genetic Data: MRC-IEU Quality Control, Version 1. 2017; Available from: https://data.bris.ac.uk/datasets/3074krb6t2frj29yh2b03x3wxj/UK Biobank Genetic Data_MRC IEU Quality Control version

1.pdf%0Ahttps://data.bris.ac.uk/data/dataset/3074krb6t2frj29yh2b03x3wxj

Miyanaka Y, Ueno Y, Tanaka S, Yoshioka K, Hatakeyama T, Shimamoto M, et al. Clinical significance of mucosal suppressors of cytokine signaling 3 expression in ulcerative colitis. World J. Gastroenterol. Baishideng Publishing Group Inc; 2007;13(21):2939–44

Miyoshi K, Takaishi M, Nakajima K, Ikeda M, Kanda T, Iiyama T, et al. Stat3 as a Therapeutic Target for the Treatment of Psoriasis : A Clinical Feasibility Study with STA-21 , a Stat3 Inhibitor. J. Invest. Dermatol. Elsevier Masson SAS; 2011;131(1):108–17 Available from: http://dx.doi.org/10.1038/jid.2010.255

Moisan J, Grenningloh R, Bettelli E, Oukka M, Ho I-C. Ets-1 is a negative regulator of Th17 differentiation. J. Exp. Med. United States; 2007;204(12):2825–35

Molin S, Merl J, Dietrich KA, Regauer M, Flaig M, Letulé V, et al. The hand eczema proteome: Imbalance of epidermal barrier proteins. Br. J. Dermatol. 2015;172(4):994–1001

Momozawa Y, Dmitrieva J, Théâtre E, Deffontaine V, Rahmouni S, Charloteaux B, et al. IBD risk loci are enriched in multigenic regulatory modules encompassing putative causative genes. Nat. Commun. 2018;9(1):1–18

Mouly E, Chemin K, Nguyen HV, Chopin M, Mesnard L, Leite-de-Moraes M, et al. The Ets-1 transcription factor controls the development and function of natural regulatory T cells. J. Exp. Med. Rockefeller University Press; 2010;207(10):2113–25

Mularczyk EJ, Singh M, Godwin ARF, Galli F, Humphreys N, Adamson AD, et al. ADAMTS10-mediated tissue disruption in Weill–Marchesani syndrome. Hum. Mol. Genet. 2018;27(21):3675–87

Mumbach MR, Satpathy AT, Boyle EA, Dai C, Gowen BG, Cho SW, et al. Enhancer connectome in primary human cells identifies target genes of disease-associated DNA elements. Nat. Genet. Nature Publishing Group; 2017;49(11):1602–12 Available from: http://www.ncbi.nlm.nih.gov/pubmed/28945252%0Ahttp://www.pubmedcentral.nih.gov/articlerender.fcgi?artid=PMC5805393

Nagarajan P, Chin SS, Wang D, Liu S, Sinha S, Garrett-Sinha LA. Ets1 blocks terminal differentiation of keratinocytes and induces expression of matrix metalloproteases and innate immune mediators. J. Cell Sci. 2010;123(20):3566–75

Nair M, Teng A, Bilanchone V, Agrawal A, Li B, Dai X. Ovol1 regulates the growth arrest of embryonic epidermal progenitor cells and represses c-myc transcription. J. Cell Biol. 2006;173(2):253–64

Naranbhai V, Fairfax BP, Makino S, Humburg P, Wong D, Ng E, et al. Genomic modulators of gene expression in human neutrophils. Nat. Commun. Nature Publishing Group; 2015;6(May):1–13 Available from: http://dx.doi.org/10.1038/ncomms8545

Nedelec Y, Sanz J, Baharian G, Tung J, Yotova V, Barreiro LB, et al. Genetic Ancestry and Natural Selection Drive Population Differences in Immune Responses to Pathogens. Cell. 2016;167:657–69

Ng DCH, Lim CP, Lin BH, Zhang T, Cao X. SCG10-like protein (SCLIP) is a STAT3-interacting protein involved in maintaining epithelial morphology in MCF-7 breast cancer cells. Biochem. J. 2009;425(1):95–108

Nguyen NYN, Maxwell MJ, Ooms LM, Davies EM, Hilton AA, Collinge JE, et al. An ENU-induced mouse mutant of SHIP1 reveals a critical role of the stem cell isoform for suppression of macrophage activation. Blood. 2011;117(20):5362–71

Ni Y, Hao J, Hou X, Du W, Yu Y, Chen T, et al. Dephosphorylated polymerase I and transcript release factor prevents allergic asthma exacerbations by limiting IL-33 release. Front. Immunol. 2018;9(JUN)

Noda S, Asano Y, Nishimura S, Taniguchi T, Fujiu K, Manabe I, et al. Simultaneous downregulation of

KLF5 and Fli1 is a key feature underlying systemic sclerosis. Nat. Commun. Nature Publishing Group; 2014;5:1–17 Available from: http://dx.doi.org/10.1038/ncomms6797

Nograles KE, Zaba LC, Guttman-Yassky E, Fuentes-Duculan J, Suárez-Fariñas M, Cardinale I, et al. Th17 cytokines interleukin (IL)-17 and IL-22 modulate distinct inflammatory and keratinocyte-response pathways. Br. J. Dermatol. John Wiley & Sons, Ltd (10.1111); 2008;159(5):1092–102 Available from: https://doi.org/10.1111/j.1365-2133.2008.08769.x

Nograles KE, Zaba LC, Shemer A, Fuentes-Duculan J, Cardinale I, Kikuchi T, et al. IL-22–producing “T22” T cells account for upregulated IL-22 in atopic dermatitis despite reduced IL-17–producing TH17 T cells. J. Allergy Clin. Immunol. 2009;123(6):1244-1252.e2 Available from: http://www.sciencedirect.com/science/article/pii/S0091674909005521

Nousbeck J, Irvine AD. Atopic Dermatitis According to GARP : New Mechanistic Insights in Disease Pathogenesis. J. Invest. Dermatol. The Authors; 2016;136(12):2340–1 Available from: http://dx.doi.org/10.1016/j.jid.2016.08.020

Pageaud Y, Plass C, Assenov Y. Enrichment analysis with EpiAnnotator. Bioinformatics. 2018;34(10):1781–3

Pala M, Zappala Z, Marongiu M, Li X, Davis JR, Cusano R, et al. Population- and individual-specific regulatory variation in Sardinia. Nat. Genet. Nature Publishing Group, a division of; 2017;49(5):700–7 Available from: https://doi.org/10.1038/ng.3840

Pandey R, Bakay M, Hain HS, Strenkowski B, Elsaqa BZB, Roizen JD, et al. CLEC16A regulates splenocyte and NK cell function in part through MEK signaling. PLoS One. 2018;13(9):1–18 Available from: http://dx.doi.org/10.1371/journal.pone.0203952

Pandey R, Bakay M, Hain HS, Strenkowski B, Yermakova A, Kushner JA, et al. The Autoimmune Disorder Susceptibility Gene CLEC16A Restrains NK Cell Function in YTS NK Cell Line and Clec16a Knockout Mice. Front. Immunol. 2019;10:68

Papke CL, Tsunezumi J, Ringuette LJ, Nagaoka H, Terajima M, Yamashiro Y, et al. Loss of fibulin-4 disrupts collagen synthesis and maturation: Implications for pathology resulting from EFEMP2 mutations. Hum. Mol. Genet. 2015;24(20):5867–79

Paternoster L, Standl M, Chen C-M, Ramasamy A, Bønnelykke K, Duijts L, et al. Meta-analysis of genome-wide association studies identifies three new risk loci for atopic dermatitis. Nat. Genet. 2011;44(2):187–92 Available from: http://www.nature.com/doifinder/10.1038/ng.1017

Paternoster L, Standl M, Waage J, Baurecht H, Hotze M, Strachan DP, et al. Multi-ancestry genome-wide association study of 21,000 cases and 95,000 controls identifies new risk loci for atopic dermatitis. Nat. Genet. 2015;47(12):1449–56

Pedroza M, To S, Assassi S, Wu M, Tweardy D, Agarwal SK. Role of STAT3 in skin fibrosis and transforming growth factor beta signalling. Rheumatology. 2018;57(10):1838–50

Pelikan RC, Kelly JA, Fu Y, Lareau CA, Tessneer KL, Wiley GB, et al. Enhancer histone-QTLs are enriched on autoimmune risk haplotypes and influence gene expression within chromatin networks. Nat. Commun. Springer US; 2018;9(1) Available from: http://dx.doi.org/10.1038/s41467-018-05328-9

Pino-Yanes M, Gignoux CR, Galanter JM, Levin AM, Campbell CD, Eng C, et al. Genome-wide association study and admixture mapping reveal new loci associated with total IgE levels in Latinos. J. Allergy Clin. Immunol. 2015;135(6):1502–10 Available from: http://www.sciencedirect.com/science/article/pii/S0091674914015759

Pitti RM, Marsters SA, Lawrence DA, Roy M, Kischkel FC, Dowd P, et al. Genomic amplification of a decoy receptor for Fas ligand in lung and colon cancer. Nature. 1998;396(6712):699–703 Available from:

https://doi.org/10.1038/25387

Puel A, Ziegler SF, Buckley RH, Leonard WJ. Defective IL7R expression in T-B+NK+ severe combined immunodeficiency. Nat. Genet. 1998;20(4):394–7

Quach H, Rotival M, Pothlichet J, Kelso J, Albert ML, Quintana-murci L. Genetic Adaptation and Neandertal Admixture Shaped the Immune System of Human Populations. Cell. 2016;167:643–56

Quraishi BM, Zhang H, Everson TM, Ray M, Lockett GA, Holloway JW, et al. Identifying CpG sites associated with eczema via random forest screening of epigenome-scale DNA methylation. Clin. Epigenetics. Clinical Epigenetics; 2015;7(1):1–11 Available from: http://dx.doi.org/10.1186/s13148-015-0108-y

Rainbow DB, Pekalski M, Cutler AJ, Burren O. A rare IL2RA haplotype identifies SNP rs61839660 as causal for autoimmunity. bioRxiv. 2017;1–11 Available from: https://www.biorxiv.org/content/10.1101/108126v1.article-info

Raj T, Rothamel K, Mostafavi S, Ye C, Lee MMNM, Replogle JM, et al. Polarization of the effects of autoimmune and neurodegenerative risk alleles in leukocytes. Science (80-.). 2014;344(6183):519–23

Rajab A, Straub V, McCann LJ, Seelow D, Varon R, Barresi R, et al. Fatal cardiac arrhythmia and long-QT syndrome in a new form of congenital generalized lipodystrophy with muscle rippling (CGL4) due to PTRF-CAVIN mutations. PLoS Genet. 2010;6(3)

Rao SSP, Huntley MH, Durand NC, Stamenova EK, Bochkov ID, Robinson JT, et al. A 3D map of the human genome at kilobase resolution reveals principles of chromatin looping. Cell. Elsevier Inc.; 2014;159(7):1665–80 Available from: http://dx.doi.org/10.1016/j.cell.2014.11.021

De Rie D, Abugessaisa I, Alam T, Arner E, Arner P, Ashoor H, et al. An integrated expression atlas of miRNAs and their promoters in human and mouse. Nat. Biotechnol. 2017;35(9):872–8

Rinaldi L, Datta D, Serrat J, Morey L, Solanas G, Avgustinova A, et al. Dnmt3a and Dnmt3b Associate with Enhancers to Regulate Human Epidermal Stem Cell Homeostasis. Cell Stem Cell. 2016;19(4):491–501

Roadmap Epigenomics Consortium, Kundaje A, Meuleman W, Ernst J, Bilenky M, Yen A, et al. Integrative analysis of 111 reference human epigenomes. Nature. 2015;518(7539):317–29

Rodríguez E, Baurecht H, Wahn AF, Kretschmer A, Hotze M, Zeilinger S, et al. An integrated epigenetic and transcriptomic analysis reveals distinct tissue-specific patterns of DNA methylation associated with atopic dermatitis. J. Invest. Dermatol. 2014;134(7):1873–83

Rogers MF, Shihab HA, Mort M, Cooper DN, Gaunt TR, Campbell C. FATHMM-XF: Accurate prediction of pathogenic point mutations via extended features. Bioinformatics. 2018;34(3):511–3

Rosa M, Chignon A, Li Z, Boulanger M-C, Arsenault BJ, Bossé Y, et al. A Mendelian randomization study of IL6 signaling in cardiovascular diseases, immune-related disorders and longevity. npj Genomic Med. Springer US; 2019;4(1):1–10 Available from: http://dx.doi.org/10.1038/s41525-019-0097-4

Rosa F, Rameil P, Bedotto M, Ferrier P, Cauchy P, Imbert J. Identification of Two Novel Regulatory Elements in the IL2RA Gene Locus. J. Pulm. Respir. Med. 2018;8(2):1–11

Royce SG, Le Saux CJ. Role of caveolin-1 in asthma and chronic inflammatory respiratory diseases. Expert Rev. Respir. Med. Informa UK, Ltd.; 2014;8(3):339–47

Rubin AJ, Barajas BC, Furlan-magaril M, Lopez-pajares V, Mumbach MR, Howard I, et al. Lineage-specific dynamic and pre-established enhancer – promoter contacts cooperate in terminal differentiation. Nat. Genet. 2017;49(10):1522–8

Sääf AM, Tengvall-Linder M, Chang HY, Adler AS, Wahlgren CF, Scheynius A, et al. Global expresion profiling in atopic eczema reveals reciprocal expression of inflammatory and lipid genes. PLoS One.

2008;3(12)

Saigusa R, Asano Y, Nakamura K, Hirabayashi M, Miura S, Yamashita T, et al. Systemic Sclerosis Dermal Fibroblasts Suppress Th1 Cytokine Production via Galectin-9 Overproduction due to Fli1 Deficiency. J. Invest. Dermatol. The Authors; 2017;137(9):1850–9 Available from: http://dx.doi.org/10.1016/j.jid.2017.04.035

Sano S, Chan KS, Carbajal S, Clifford J, Peavey M, Kiguchi K, et al. Stat3 links activated keratinocytes and immunocytes required for development of psoriasis in a novel transgenic mouse model. Nat. Med. 2005;11(1):43–9 Available from: https://doi.org/10.1038/nm1162

Dos Santos RS, Marroqui L, Velayos T, Olazagoitia-Garmendia A, Jauregi-Miguel A, Castellanos-Rubio A, et al. DEXI, a candidate gene for type 1 diabetes, modulates rat and human pancreatic beta cell inflammation via regulation of the type I IFN/STAT signalling pathway. Diabetologia. Diabetologia; 2019;62(3):459–72

Satoh T, Kato H, Kumagai Y, Yoneyama M, Sato S, Matsushita K, et al. LGP2 is a positive regulator of RIG-I- and MDA5-mediated antiviral responses. Proc. Natl. Acad. Sci. 2010;107(4):1512–7

Schaarschmidt H, Ellinghaus D, Rodríguez E, Kretschmer A, Baurecht H, Lipinski S, et al. A genome-wide association study reveals 2 new susceptibility loci for atopic dermatitis. J. Allergy Clin. Immunol. 2015;136(3):802–6

Schimke LF, Sawalle-Belohradsky J, Roesler J, Wollenberg A, Rack A, Borte M, et al. Diagnostic approach to the hyper-IgE syndromes: Immunologic and clinical key findings to differentiate hyper-IgE syndromes from atopic dermatitis. J. Allergy Clin. Immunol. Elsevier Ltd; 2010;126(3):611-617.e1 Available from: http://dx.doi.org/10.1016/j.jaci.2010.06.029

Schmiedel BJ, Singh D, Madrigal A, Valdovino-Gonzalez AG, White BM, Zapardiel-Gonzalo J, et al. Impact of Genetic Polymorphisms on Human Immune Cell Gene Expression. Cell. 2018;1701–15 Available from: https://linkinghub.elsevier.com/retrieve/pii/S009286741831331X

Schuster C, Gerold KD, Schober K, Probst L, Boerner K, Kim MJ, et al. The Autoimmunity-Associated Gene CLEC16A Modulates Thymic Epithelial Cell Autophagy and Alters T Cell Selection. Immunity. Elsevier Inc.; 2015;42(5):942–52 Available from: http://dx.doi.org/10.1016/j.immuni.2015.04.011

Seki Y, Inoue H, Nagata N, Hayashi K, Fukuyama S, Komine O, et al. SOCS-3 regulates onset and maintenance of TH2- mediated allergic responses. Nat. Med. 2003;9(8):1047–54

Seo S, Baye LM, Schulz NP, Beck JS, Zhang Q, Slusarski DC, et al. BBS6, BBS10, and BBS12 form a complex with CCT/TRiC family chaperonins and mediate BBSome assembly. Proc. Natl. Acad. Sci. 2010;107(4):1488–93 Available from: http://www.pnas.org/cgi/doi/10.1073/pnas.0910268107

Shen J, Qiao Y, Ran Z, Wang T. Up-regulation and Pre-activation of TRAF3 and TRAF5 in Inflammatory Bowel Disease. Int. J. Med. Sci. 2013;10(2)

Shen Z, Ye C, Mccain K, Greenberg ML. The Role of Cardiolipin in Cardiovascular Health. Biomed Res. Int. 2015;2015(891707)

Shin H-W, Kobayashi H, Kitamura M, Waguri S, Suganuma T, Uchiyama Y, et al. Roles of ARFRP1 (ADP-ribosylation factor-related protein 1) in post-Golgi membrane trafficking. J. Cell Sci. The Company of Biologists Ltd; 2005;118(17):4039–48 Available from: http://jcs.biologists.org/content/118/17/4039

Simeonov DR, Gowen BG, Boontanrart M, Roth TL, Gagnon JD, Mumbach MR, et al. Discovery of stimulation-responsive immune enhancers with CRISPR activation. Nature. Nature Publishing Group; 2017;549(7670):111–5 Available from: http://dx.doi.org/10.1038/nature23875

Soleimanpour SA, Gupta A, Bakay M, Ferrari AM, Groff DN, Fadista J, et al. The diabetes susceptibility gene Clec16a regulates mitophagy. Cell. 2014;157(7):1577–90

Soskic B, Cano-Gamez E, Smyth DJ, Rowan WC, Nakic N, Esparza-Gordillo J, et al. Chromatin activity at GWAS loci identifies T cell states driving complex immune diseases. bioRxiv. 2019;566810 Available from: https://www.biorxiv.org/content/10.1101/566810v1.abstract

Soumelis V, Reche PA, Kanzler H, Yuan W, Edward G, Homey B, et al. Human epithelial cells trigger dendritic cell--mediated allergic inflammation by producing TSLP. Nat. Immunol. Nature Publishing Group; 2002;3(7):673

Stegle O, Parts L, Piipari M, Winn J, Durbin R. Using probabilistic estimation of expression residuals (PEER) to obtain increased power and interpretability of gene expression analyses. Nat. Protoc. 2012;7(3):500–7

Stevens ML, Zhang Z, Johansson E, Ray S, Jagpal A, Ruff BP, et al. Disease-associated KIF3A variants alter gene methylation and expression impacting skin barrier and atopic dermatitis risk. Nat. Commun. 2020;11(1):4092 Available from: https://doi.org/10.1038/s41467-020-17895-x

Stoeckle C, Gouttefangeas C, Hammer M, Weber E, Melms A, Tolosa E. Cathepsin W expressed exclusively in CD8+ T cells and NK cells, is secreted during target cell killing but is not essential for cytotoxicity in human CTLs. Exp. Hematol. 2009;37(2):266–75 Available from: http://www.sciencedirect.com/science/article/pii/S0301472X08004864

Suárez-Fariñas M, Dhingra N, Gittler J, Shemer A, Cardinale I, de Guzman Strong C, et al. Intrinsic atopic dermatitis shows similar TH2 and higher TH17 immune activation compared with extrinsic atopic dermatitis. J. Allergy Clin. Immunol. 2013;132(2):361–70 Available from: http://www.sciencedirect.com/science/article/pii/S0091674913006933

Suárez-Fariñas M, Tintle SJ, Shemer A, Chiricozzi A, Nograles K, Cardinale I, et al. Nonlesional atopic dermatitis skin is characterized by broad terminal differentiation defects and variable immune abnormalities. J. Allergy Clin. Immunol. 2011;127(4):954-964.e4 Available from: https://www.scopus.com/inward/record.uri?eid=2-s2.0-79953668221&doi=10.1016%2Fj.jaci.2010.12.1124&partnerID=40&md5=fee99dbf5e76ee8ecac59e444e101f2a

Suhre K, Arnold M, Bhagwat AM, Cotton RJ, Engelke R, Raffler J, et al. Connecting genetic risk to disease end points through the human blood plasma proteome. Nat. Commun. 2017;8

Szakos E, Lakos G, Aleksza M, Gyimesi E, Páll G, Fodor B, et al. Association between the occurrence of the anticardiolipin IgM and mite allergen-specific IgE antibodies in children with extrinsic type of atopic eczema/dermatitis syndrome. Allergy Eur. J. Allergy Clin. Immunol. 2004;59(2):164–7

Tam RCY, Li MWM, Gao YP, Pang YT, Yan S, Ge W, et al. Human CLEC16A regulates autophagy through modulating mTOR activity. Exp. Cell Res. Elsevier Inc.; 2017;352(2):304–12 Available from: http://dx.doi.org/10.1016/j.yexcr.2017.02.017

Thompson PJ, Macfarlan TS, Lorincz MC. Long Terminal Repeats: From Parasitic Elements to Building Blocks of the Transcriptional Regulatory Repertoire. Mol. Cell. Elsevier Inc.; 2016;62(5):766–76 Available from: http://dx.doi.org/10.1016/j.molcel.2016.03.029

Tokuhiro S, Yamada R, Chang X, Suzuki A, Kochi Y, Sawada T, et al. An intronic SNP in a RUNX1 binding site of SLC22A4, encoding an organic cation transporter, is associated with rheumatoid arthritis. Nat. Genet. 2003;35(4):341–8

Tournier C, Dong C, Turner TK, Jones SN, Flavell RA, Davis RJ. MKK7 is an essential component of the JNK signal transduction pathway activated by proinflammatory cytokines. Genes Dev. Cold Spring Harbor Lab; 2001;15(11):1419–26

Tseng P, Matsuzawa A, Zhang W, Mino T, Vignali DAA, Karin M. Different modes of ubiquitination of the adaptor TRAF3 selectively activate the expression of type I interferons and proinflammatory cytokines. Nat.

Immunol. 2010;11(1):70–5

Tsuji G, Hashimoto-hachiya A, Kiyomatsu-oda M, Takemura M, Ohno F, Ito T. Aryl hydrocarbon receptor activation restores filaggrin expression via OVOL1 in atopic dermatitis. Cell Death Dis. Nature Publishing Group; 2017;8(7):e2931-8 Available from: http://dx.doi.org/10.1038/cddis.2017.322

Ulrich F, Krieg M, Schötz EM, Link V, Castanon I, Schnabel V, et al. Wnt11 functions in gastrulation by controlling cell cohesion through Rab5c and E-Cadherin. Dev. Cell. 2005;9(4):555–64

Venkataraman T, Valdes M, Elsby R, Kakuta S, Caceres G, Saijo S, et al. Loss of DExD/H Box RNA Helicase LGP2 Manifests Disparate Antiviral Responses. J. Immunol. 2007;178(10):6444–55 Available from: http://www.jimmunol.org/cgi/doi/10.4049/jimmunol.178.10.6444

Verhagen J, Akdis M, Traidl-Hoffmann C, Schmid-Grendelmeier P, Hijnen D, Knol EF, et al. Absence of T-regulatory cell expression and function in atopic dermatitis skin. J. Allergy Clin. Immunol. 2006;117(1):176–83

Vicente CT, Edwards SL, Hillman KM, Kaufmann S, Mitchell H, Bain L, et al. Long-Range Modulation of PAG1 Expression by 8q21 Allergy Risk Variants. Am. J. Hum. Genet. The American Society of Human Genetics; 2015;97(2):329–36 Available from: http://dx.doi.org/10.1016/j.ajhg.2015.06.010

Vitour D, Meurs EF. Regulation of interferon production by RIG-I and LGP2: a lesson in self-control. Sci. STKE. 2007;2007(384):1–5

Võsa U, Claringbould A, Westra H, Bonder MJ, Zeng B, Kirsten H, et al. Unraveling the polygenic architecture of complex traits using blood eQTL meta- analysis. bioRxiv. 2018;1–57 Available from: https://www.biorxiv.org/content/10.1101/447367v1

Walsh AM, Whitaker JW, Huang CC, Cherkas Y, Lamberth SL, Brodmerkel C, et al. Integrative genomic deconvolution of rheumatoid arthritis GWAS loci into gene and cell type associations. Genome Biol. Genome Biology; 2016a;17:79 Available from: http://dx.doi.org/10.1186/s13059-016-0948-6

Walsh AM, Whitaker JW, Huang CC, Cherkas Y, Lamberth SL, Brodmerkel C, et al. Integrative genomic deconvolution of rheumatoid arthritis GWAS loci into gene and cell type associations. Genome Biol. Genome Biology; 2016b;17:79 Available from: http://dx.doi.org/10.1186/s13059-016-0948-6

Wang X, He L, Goggin SM, Saadat A, Wang L, Sinnott-armstrong N, et al. High-resolution genome-wide functional dissection of transcriptional regulatory regions and nucleotides in human. Nat. Commun. Springer US; 2018a; Available from: http://dx.doi.org/10.1038/s41467-018-07746-1

Wang X, Lopez OL, Sweet RA, Becker JT, Dekosky ST, Barmada MM, et al. Genetic determinants of disease progression in Alzheimer's disease. J. Alzheimer's Dis. 2015a;43(2):649–55

Wang J, Vicente-García C, Seruggia D, Moltó E, Fernandez-Miñán A, Neto A, et al. MIR retrotransposon sequences provide insulators to the human genome. Proc. Natl. Acad. Sci. 2015b;112(32):E4428–37 Available from: http://www.pnas.org/lookup/doi/10.1073/pnas.1507253112

Wang J, Zhang P, Lu Y, Li Y, Zheng Y, Kan Y, et al. piRBase: a comprehensive database of piRNA sequences. Nucleic Acids Res. Oxford University Press; 2018b;47(D1):175–80

Weidinger S, Willis-Owen SAG, Kamatani Y, Baurecht H, Morar N, Liang L, et al. A genome-wide association study of atopic dermatitis identifies loci with overlapping effects on asthma and psoriasis. Hum. Mol. Genet. 2013;22(23):4841–56 Available from: https://doi.org/10.1093/hmg/ddt317

Wenzel J, Bieber T. Anti-cardiolipin antibodies in atopic dermatitis. Allergy Eur. J. Allergy Clin. Immunol. 2004;59(2):162–3

Winge MCG, Hoppe T, Berne B, Vahlquist A, Nordenskjöld M, Bradley M, et al. Filaggrin genotype determines functional and molecular alterations in skin of patients with atopic dermatitis and ichthyosis

vulgaris. PLoS One. 2011;6(12):1–8

Wittmann M, Purwar R, Hartmann C, Gutzmer R, Werfel T. Human Keratinocytes Respond to Interleukin-18: Implication for the Course of Chronic Inflammatory Skin Diseases. J. Invest. Dermatol. Elsevier; 2005;124(6):1225–33 Available from: https://doi.org/10.1111/j.0022-202X.2005.23715.x

Xia K, Shabalin AA, Zou F, Wright FA, Xia K, Sullivan PF, et al. seeQTL: a searchable database for human eQTLs. Bioinformatics. 2011;28(3):451–2 Available from: https://dx.doi.org/10.1093/bioinformatics/btr678

Xie C, Yuan J, Li H, Li M, Zhao G, Bu D, et al. NONCODEv4: Exploring the world of long non-coding RNA genes. Nucleic Acids Res. 2014;42(D1):98–103

Xing Y, Igarashi H, Wang X, Sakaguchi N. Protein phosphatase subunit G5PR is needed for inhibition of B cell receptor–induced apoptosis. J. Exp. Med. 2005;202(5):707–19

Xing Y, Wang X, Igarashi H, Kawamoto H, Sakaguchi N. Protein phosphatase subunit G5PR that regulates the JNK-mediated apoptosis signal is essential for the survival of CD4 and CD8 double-positive thymocytes. Mol. Immunol. 2008;45(7):2028–37

Xu Y, Cheng G, Baltimore D. Targeted disruption of TRAF3 leads to postnatal lethality and defective T-dependent immune responses. Immunity. 1996;5(5):407–15

Yang CP, Li X, Wu Y, Shen Q, Zeng Y, Xiong Q, et al. Comprehensive integrative analyses identify GLT8D1 and CSNK2B as schizophrenia risk genes. Nat. Commun. Springer US; 2018;9(1) Available from: http://dx.doi.org/10.1038/s41467-018-03247-3

Yao C, Joehanes R, Johnson AD, Huan T, Liu C, Freedman JE, et al. Dynamic Role of trans Regulation of Gene Expression in Relation to Complex Traits. Am. J. Hum. Genet. ElsevierCompany.; 2017;100(4):571–80 Available from: http://dx.doi.org/10.1016/j.ajhg.2017.02.003

Ye CJ, Feng T, Kwon H, Raj T, Wilson M, Asinovski N, et al. Intersection of population variation and autoimmunity genetics in human T cell activation. Science (80-.). 2014;345(6202):1254665

Yin Y, Liu W, Dai Y. SOCS3 and its role in associated diseases. Hum. Immunol. American Society for Histocompatibility and Immunogenetics; 2015;76(10):775–80 Available from: http://dx.doi.org/10.1016/j.humimm.2015.09.037

Zahn C, Jaschke A, Weiske J, Hommel A, Hesse D, Augustin R, et al. ADP-ribosylation factor-like GTPase ARFRP1 is required for trans-Golgi to plasma membrane trafficking of E-cadherin. J. Biol. Chem. ASBMB; 2008;283(40):27179–88

Zariwala MA, Knowles MR, Omran H. Genetic Defects in Ciliary Structure and Function. Annu. Rev. Physiol. 2007;69:423–50

Zhang H, Luo XM. Control of commensal microbiota by the adaptive immune system. Gut Microbes. 2015;6(2):156–60

Zhang Y, Ni S, Huang B, Wang L, Zhang X, Li XX, et al. Overexpression of SCLIP promotes growth and motility in glioblastoma cells. Cancer Biol. Ther. 2015;16(1):97–105

Zhang J, Somani AK, Siminovitch KA. Roles of the SHP-1 tyrosine phosphatase in the negative regulation of cell signalling. Semin. Immunol. 2000;12(4):361–78

Zhernakova D V., Deelen P, Vermaat M, Van Iterson M, Van Galen M, Arindrarto W, et al. Identification of context-dependent expression quantitative trait loci in whole blood. Nat. Genet. 2017;49(1):139–45

Zhou X, Stephens M. Genome-wide efficient mixed-model analysis for association studies. Nat. Genet. Nature Publishing Group; 2012;44(7):821–4 Available from: http://dx.doi.org/10.1038/ng.2310

Zhu BM, Ishida Y, Robinson GW, Pacher-Zavisin M, Yoshimura A, Murphy PM, et al. SOCS3 negatively

regulates the gp130-STAT3 pathway in mouse skin wound healing. J. Invest. Dermatol. Elsevier Masson SAS; 2008;128(7):1821–9 Available from: http://dx.doi.org/10.1038/sj.jid.5701224

Zhu Z, Lee PH, Chaffin MD, Chung W, Loh PR, Lu Q, et al. A genome-wide cross-trait analysis from UK Biobank highlights the shared genetic architecture of asthma and allergic diseases. Nat. Genet. Springer US; 2018;50(6):857–64 Available from: http://dx.doi.org/10.1038/s41588-018-0121-0

Zhu H, Lin P, De G, Choi KH, Takeshima H, Weisleder N, et al. Polymerase transcriptase release factor (PTRF) anchors MG53 protein to cell injury site for initiation of membrane repair. J. Biol. Chem. 2011;286(15):12820–4

Ziebarth JD, Bhattacharya A, Cui Y. CTCFBSDB 2.0: a database for CTCF-binding sites and genome organization. Nucleic Acids Res. 2012;41(Database issue):D188-94 Available from: http://nar.oxfordjournals.org/content/early/2012/11/26/nar.gks1165.abstract

Zuvich RL, Bush WS, McCauley JL, Beecham AH, De Jager PL, the International Multiple Sclerosis Genetics Consortium, et al. Interrogating the complex role of chromosome 16p13.13 in multiple sclerosis susceptibility: independent genetic signals in the CIITA–CLEC16A–SOCS1 gene complex . Hum. Mol. Genet. 2011;20(17):3517–24 Available from: https://doi.org/10.1093/hmg/ddr250
